# Structure-Based Virtual Screening and Mechanistic Characterization of Methotrexate and Selinexor as Potent Anti-Melanogenic Agents via Multi-Pathway Suppression of MITF

**DOI:** 10.3390/cells15121070

**Published:** 2026-06-11

**Authors:** Zhongwei Zhang, Huiran Li, Zhonglan Shi, Xuan Bai, Peipei Yin, Lingguang Yang

**Affiliations:** 1 Jiangxi Key Laboratory of Natural Microbial Medicine Research, School of Pharmacy, Yichun University, Yichun 336000, China; zzw220825@126.com (Z.Z.); lihuiran0402@163.com (H.L.); shizhonglan856@gmail.com (Z.S.); 18720526739@163.com (X.B.); 2 Jiangxi Provincial Engineering Research Center for Recycling Technology of Traditional Chinese Medicine Herbal Residue, School of Pharmacy, Yichun University, Yichun 336000, China; 3 Yichun City Natural Medicines Bionic Delivery Technology Innovation Center, School of Pharmacy, Yichun University, Yichun 336000, China; 4 Yichun University Center for Innovative Technologies in Gene-Based Precision Therapeutics, School of Pharmacy, Yichun University, Yichun 336000, China

**Keywords:** tyrosinase inhibitor, virtual screening, melanogenesis, MITF, drug repurposing

## Abstract

**Highlights:**

**What are the main findings?**

- Structure-based virtual screening against a human tyrosinase homology model identified methotrexate and selinexor as potent suppressors of melanogenesis, achieving nanomolar efficacy in MNT-1 melanoma cells.
- They dismantle the melanogenic program through a coordinated multi-node attack on MITF: dual transcriptional blockade via the cAMP/PKA/CREB and Wnt/β-catenin pathways, accelerated proteasomal degradation through AKT/ERK activation, and reinforcement of the intracellular antioxidant defense system.

**What are the implications of the main findings?**

- Methotrexate and selinexor have well-documented systemic toxicities in their approved oncological indications. Therefore, their potential application in hyperpigmentation would require rigorous preclinical evaluation of topical formulations to ensure minimal systemic absorption and local tolerability.
- Convergent targeting of MITF at both the transcriptional and post-translational levels constitutes a robust anti-melanogenic strategy and provides a framework for next-generation depigmenting therapeutics.

**Abstract:**

Tyrosinase is a pivotal therapeutic target for hyperpigmentation disorders, yet current inhibitors frequently exhibit limited potency and suboptimal safety. Here, we employed structure-based virtual screening of an FDA-approved drug library against a refined human tyrosinase homology model, identifying methotrexate and selinexor as potent anti-melanogenic candidates. Both compounds markedly suppressed cellular tyrosinase activity and melanin synthesis (IC_50_ < 1 µM) in MNT-1 melanoma cells. Mechanistically, they orchestrate a multi-pronged downregulation of microphthalmia-associated transcription factor (MITF) by attenuating cAMP/PKA/CREB signaling, promoting β-catenin degradation, and accelerating MITF proteolysis via AKT/ERK activation. Additionally, they bolster the intracellular antioxidant defense system. These findings unveil a sophisticated regulatory network and suggest that with strict control of systemic exposure through optimized topical formulations, these FDA-approved agents could be further investigated as potential localized treatments for pigmentary disorders.

## 1. Introduction

Skin, the largest organ of the human body, acts as a primary barrier against environmental insults. Melanin, a heterogeneous biopolymer synthesized within melanocytes, plays a pivotal role in determining skin color and protecting against ultraviolet (UV)-induced DNA damage [1]. While essential for photoprotection, the dysregulation of melanin homeostasis is implicated in a spectrum of aesthetic and pathological conditions, ranging from common hyperpigmentation disorders like melasma and freckles to the progression and therapeutic resistance of malignant melanoma [2,3]. The increasing global prevalence of these pigmentary abnormalities has spurred significant demand for safe and efficacious depigmenting agents. Epidemiological surveys indicate that up to 40% of women in East Asian countries utilize skin-lightening products, underscoring the substantial clinical and commercial need for novel interventions [4]. To address this need, a comprehensive understanding of the molecular machinery governing melanin production is essential for identifying effective therapeutic targets.

**Figure 1:**
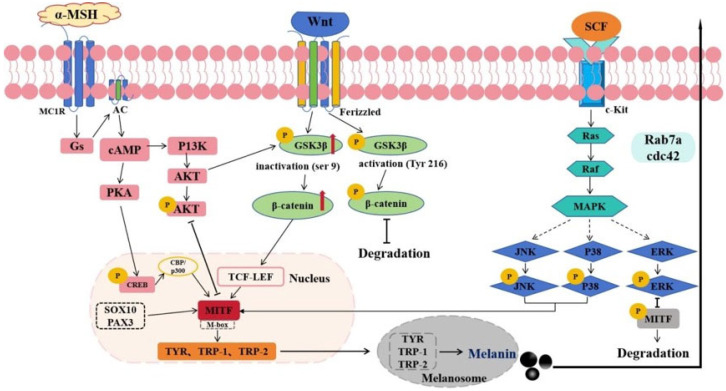
Multi-pathway regulatory network of melanogenesis. Schematic illustration of the major signaling cascades converging on microphthalmia-associated transcription factor (MITF), the master regulator of melanocyte pigmentation. Key pathways include the α-MSH/MC1R/cAMP/PKA/CREB axis, the Wnt/β-catenin pathway, and the MAPK/ERK and PI3K/AKT pathways, which collectively modulate MITF transcription, protein stability, and subsequent expression of melanogenic enzymes.

The biosynthesis of melanin is governed by a complex regulatory network centered on the microphthalmia-associated transcription factor (MITF). Acting as a master transcriptional regulator, MITF orchestrates the expression of key melanogenic enzymes, notably the rate-limiting enzyme tyrosinase (TYR), as well as tyrosinase-related protein 1 (TRP1) and 2 (TRP2/DCT) [5,6]. The expression and activity of MITF are exquisitely tuned by a convergence of upstream signaling cascades. The α-melanocyte-stimulating hormone (α-MSH)/cAMP/PKA/CREB axis serves as a primary transcriptional driver, while the Wnt/β-catenin pathway provides essential co-activator signals for MITF gene transcription [7,8]. Simultaneously, post-translational regulation is mediated by pathways such as MAPK/ERK and PI3K/AKT, which can promote the phosphorylation-dependent proteasomal degradation of MITF, thereby fine-tuning melanin output (Figure 1) [9]. Given its central position in this intricate web, MITF and its downstream effector tyrosinase have emerged as prime therapeutic targets for modulating pigmentation. As the key rate-limiting enzyme in this cascade, tyrosinase catalyzes the initial and rate-limiting steps of melanogenesis, converting L-tyrosine to L-DOPA quinone. Consequently, direct inhibition of its catalytic activity represents a cornerstone strategy for controlling hyperpigmentation [10].

However, the clinical and cosmetic application of many established tyrosinase inhibitors is hampered by significant drawbacks. On one hand, natural compounds, such as kojic acid and arbutin, often suffer from suboptimal potency and poor stability [11]. On the other hand, potent synthetic agents like hydroquinone are associated with severe adverse effects, including cytotoxicity, contact dermatitis, and mutagenicity [12]. Compounding this issue, a critical challenge in inhibitor development stems from the notable structural divergence between the commonly used mushroom (*Agaricus bisporus*) tyrosinase model and its human counterpart. This discrepancy often leads to promising in vitro results that fail to translate into cellular or clinical efficacy, underscoring the urgent need for more predictive and human-relevant screening strategies [13].

Recent advances in computer-aided drug design (CADD) offer a transformative approach to overcoming these limitations. Structure-based virtual screening (VS), in particular, enables the rapid, cost-effective, and high-throughput exploration of vast chemical spaces to identify lead candidates with high predicted affinity and selectivity for a specific target, such as the hsTYR active site [14,15]. The repurposing of FDA-approved drugs through VS further presents an attractive and expedited path to clinical translation by leveraging compounds with well-characterized safety and pharmacokinetic profiles [16]. The power of this strategy is exemplified by recent successes, such as the discovery of the antifungal drug amphotericin B as a potent hsTYR inhibitor through a similar integrated computational and experimental workflow [17]. Additionally, the integration of advanced protein structure prediction tools like AlphaFold2 enables more accurate modeling of hsTYR, enhancing the reliability of docking-based virtual screening campaigns [18]. Inspired by these advances, we sought to apply a similar integrated computational and experimental framework to discover new anti-melanogenic agents from FDA-approved drugs.

In this study, we employed an integrated strategy combining structure-based virtual screening of an FDA-approved drug library with comprehensive in vitro validation to identify novel anti-melanogenic agents. Our investigation aimed to: (i) identify and characterize potent tyrosinase inhibitors from a library of clinically approved drugs; and (ii) elucidate the underlying molecular mechanisms governing their inhibitory effects. We report the identification of methotrexate and selinexor as robust suppressors of melanogenesis that operate through a multi-faceted mechanism converging on MITF. We demonstrate that these agents not only directly inhibit tyrosinase activity, but also potently downregulate MITF through a dual attack: suppressing its transcription via the cAMP/PKA/CREB and Wnt/β-catenin pathways while concurrently accelerating its proteasomal degradation via AKT/ERK activation. Our findings unveil a complex mode of action for these repurposed drugs and provide a compelling rationale for their potential application in the treatment of pigmentary disorders.

## 2. Materials and Methods

### 2.1. Chemicals and Reagents

Methotrexate (purity ≥ 99%), selinexor (purity ≥ 99%), and dimethyl sulfoxide (DMSO) were purchased from MedChemExpress (Monmouth Junction, NJ, USA). Tucatinib, aprepitant, belumosudil, corilagin, and natamycin were obtained from Selleck Chemicals (Houston, TX, USA). Dulbecco’s modified Eagle medium (DMEM), fetal bovine serum (FBS), non-essential amino acids (NEAAs), and penicillin–streptomycin (P/S) were procured from Servicebio (Wuhan, China). 3-(4,5-Dimethylthiazol-2-yl)-2,5-diphenyltetrazolium bromide (MTT) and L-3,4-dihydroxyphenylalanine (L-DOPA) were from Yuanye Bio-Technology Co., Ltd. (Shanghai, China). Triton X-100 was also obtained from Yuanye. Antibodies for Western blotting are listed in Table S1. The MITF-M overexpression plasmid was constructed by Biorigin (Beijing, China). All other chemicals and reagents, unless specified, were of analytical grade and purchased from standard commercial sources.

### 2.2. Homology Modeling and Structure-Based Virtual Screening

A homology model of human tyrosinase (hsTYR) was constructed using the SWISS-MODEL server (https://swissmodel.expasy.org/ (accessed on 20 December 2025)) [19], employing the crystal structure of human tyrosinase-related protein 1 (TRP1, PDB ID: 2Y9W) as a template. The sequence of hsTYR (UniProt ID: P14679) was retrieved, and the predicted model was further refined using the Gromacs—2025.1 software package. Two copper ions were modeled into the active site by structural alignment with the abTYR crystal structure (PDB ID: 2Y9X) [20,21]. The complete model was solvated in a TIP3P water box, neutralized with 0.15 M NaCl, and subjected to energy minimization and a 100 ns molecular dynamics simulation under the CHARMM36 force field to obtain an equilibrated structure for docking.

A library of 4180 compounds, comprising 1604 FDA-approved drugs and 2576 plant-derived natural products, was compiled from the PubChem database (https://pubchem.ncbi.nlm.nih.gov/ (accessed on 20 December 2025)). All ligand structures were prepared using the LigPrep module in PyRx, which involved generating 3D conformations, protonation at pH 7.4, and energy minimization [22]. A grid box of 25 × 25 × 25 Å^3^ was centered on the binuclear copper active site of the refined hsTYR model. High-throughput molecular docking was performed using AutoDock Vina (version 1.2.5) within the PyRx platform [23,24]. For each compound, 24 binding poses were generated, and the conformation with the lowest binding affinity (kcal/mol) was selected for further analysis. Compounds were ranked by their predicted binding energy, and those with scores < −8.0 kcal/mol were considered as potential hits and subjected to further evaluation based on drug-likeness and commercial availability [18].

### 2.3. In Vitro Tyrosinase Activity

Inhibition of mushroom tyrosinase enzymatic activity was assessed using L-DOPA as the substrate. Mushroom tyrosinase was incubated with test compounds (0–100 µM) in 50 mM sodium phosphate buffer (pH 6.8). The reaction was initiated by adding L-DOPA (0.5 mM), and dopachrome formation was monitored spectrophotometrically at 475 nm using a Tecan Infinite M200 PRO microplate reader (Tecan, Männedorf, Switzerland). The assay was performed for 10 min at 37 °C, with absorbance readings taken every 30 s. The initial linear rate (first 5 min) was used for IC_50_ calculation. Kojic acid was used as a positive control reference inhibitor in all cell-free tyrosinase activity assays.

### 2.4. Cell Culture

MNT-1 human pigmented melanoma cells were purchased from Shanghai Bin-Sui Biotechnology Co., Ltd. (Shanghai, China). Cells were cultured in DMEM supplemented with 20% FBS, 1% NEAA, and 1% penicillin/streptomycin at 37 °C in a 5% CO_2_ humidified incubator. Cells from passages 5–20 were utilized.

### 2.5. Cell Viability Assay

Cytotoxicity was evaluated using the MTT assay. MNT-1 cells were treated with test compounds for 48 h. Formazan absorbance was measured at 570 nm [25]. IC_50_ values were calculated using GraphPad Prism 8.0.

### 2.6. Intracellular Tyrosinase Activity

Tyrosinase activity was assessed by measuring L-DOPA oxidation following a 48 h incubation of MNT-1 cells with sub-cytotoxic concentrations of the test compounds. Treated cells were lysed, and cell lysates were incubated with 0.5 mM L-DOPA. Dopachrome formation was monitored at 475 nm [17].

### 2.7. Melanin Content Quantification

Following 48 h of exposure to the test compounds, intracellular melanin was solubilized in 1 M NaOH/10% DMSO at 80 °C for 1 h. Absorbance was measured at 405 nm and normalized to protein concentration determined by the BCA protein assay kit (Biorigin, Beijing, China) [26].

### 2.8. Intracellular ROS Measurement

ROS levels were detected using a commercial DCFH-DA probe kit (Biorigin, Beijing, China). Following a 48 h treatment with the candidate drugs, cells were exposed to Rosup reagent (provided in the kit as a positive control stimulator) to induce a robust, reproducible baseline of intracellular ROS accumulation. The Rosup reagent serves as an oxidative stress inducer, enabling quantification of the antioxidant capacity of methotrexate and selinexor under standardized pro-oxidant conditions. Following treatment, cells were incubated with 10 µM DCFH-DA, and fluorescence intensity was quantified at 485/535 nm and normalized to protein content.

### 2.9. Determination of GSH, MDA, SOD, and CAT

MNT-1 cells were treated with test compounds for 48 h before collection. Levels of GSH, MDA, and activities of SOD and CAT were measured in cell lysates using commercial assay kits according to the manufacturers’ protocols. Total protein concentration in the lysates was determined via a BCA protein assay kit to allow for precise evaluation normalization. All colorimetric data were recorded using a Tecan Infinite M200 PRO microplate reader (Tecan, Männedorf, Switzerland).

### 2.10. Quantitative Real-Time PCR

Following 48 h of treatment with the indicated concentrations of methotrexate or selinexor, total RNA was extracted and reverse transcribed. qRT-PCR was performed using SYBR Green Master Mix with gene-specific primers for MITF, TYR, TRP1, TRP2, and β-actin (Table S2). Relative expression was determined using the 2^−ΔΔCt^ method.

### 2.11. Western Blot Analysis

After 48 h of treatment, cells were lysed using RIPA lysis buffer. The resulting protein lysates were separated by SDS-PAGE and electrotransferred onto PVDF membranes. The membranes were then blocked and probed with specific primary antibodies (Table S1), followed by HRP-conjugated secondary antibodies. Immunoreactive bands were visualized using enhanced chemiluminescence (ECL) and quantitatively analyzed using ImageJ software (https://imagej.net).

### 2.12. Immunofluorescence and Confocal Microscopy

Cells were fixed, permeabilized, and stained with anti-MITF primary antibody followed by Alexa Fluor 488-conjugated secondary antibody. Nuclei were counterstained with DAPI. Images were acquired using a Leica TCS SP8 confocal microscope (Leica Microsystems, Wetzlar, Germany), and nuclear MITF intensity was quantified with ImageJ.

### 2.13. MITF-M Overexpression

The MITF-M coding sequence (Table S3) was cloned into pcDNA3.1(+). MNT-1 cells were transiently transfected with the overexpression plasmid or empty vector using Lipofectamine 2000 (Biorigin, Beijing, China). Subsequent treatments were initiated 6 h post-transfection.

### 2.14. Statistical Analysis

Data are presented as mean ± SEM from at least three independent experiments. Statistical significance was determined using an unpaired two-tailed Student’s *t*-test for two-group comparisons or one-way ANOVA with Tukey’s post hoc test for multiple comparisons (* *p* < 0.05, ** *p* < 0.01). Analysis was performed using GraphPad Prism 8.0.

## 3. Results and Discussion

### 3.1. Docking-Based Virtual Screening and Hit Identification

A three-dimensional model of the hsTYR catalytic domain was built using the crystal structure of human tyrosinase-related protein 1 (TRP1; PDB: 2Y9W) as a template, which shares ~44% sequence identity with hsTYR in the catalytic core [17]. The binuclear copper site was accurately modeled by structural alignment with the high-resolution abTYR structure (PDB: 2Y9X), ensuring correct placement of the two copper ions (CuA, CuB) and their coordinating histidines (His180, His202, His211, His363, His367, His390). The model was solvated in a TIP3P water box, neutralized, and subjected to 100 ns molecular dynamics (MD) under the CHARMM36 force field to obtain an equilibrated, physiologically relevant conformation for docking. The protein backbone RMSD stabilized at ~2.1 Å after 50 ns, indicating a well-equilibrated model suitable for virtual screening.

A curated library of 4172 compounds (1604 FDA-approved drugs + 2568 plant-derived natural products) was docked into the binuclear copper active site of the equilibrated hsTYR model using AutoDock Vina. The docking grid (25 × 25 × 25 Å^3^) was centered on CuA/CuB, covering the entire catalytic pocket. For each ligand, 24 binding poses were generated and scored; the conformation with the lowest predicted binding free energy (ΔGbind) was selected. The screen yielded affinities ranging from 36.8 to −9.0 kcal/mol (Table S4).

**Figure 2:**
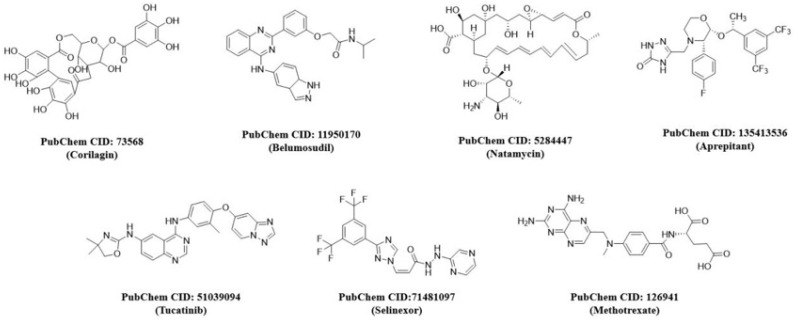
Two-dimensional (2D) chemical structures of FDA-approved drug candidates identified as potential tyrosinase inhibitors via structure-based virtual screening. The seven compounds—tucatinib, aprepitant, belumosudil, corilagin, methotrexate, natamycin, and selinexor—were prioritized based on binding energy (<−8.0 kcal/mol), drug-likeness, and commercial availability using PyRx with AutoDock Vina.

To prioritize genuine inhibitors, we applied a stringent ΔGbind cutoff of <−8.0 kcal/mol, a threshold previously shown to enrich for true tyrosinase inhibitors [18,24]. This identified seven candidates with predicted binding energies from −8.3 to −9.0 kcal/mol: tucatinib (−8.6), aprepitant (−8.4), belumosudil (−8.3), corilagin (−8.3), methotrexate (−9.0), natamycin (−8.4), and selinexor (−8.8) (Figure 2, Tables S4 and S5).

The binding energies of our top hits (−8.3 to −9.0 kcal/mol) were substantially more favorable than those reported for several known tyrosinase inhibitors identified by similar virtual screens. For example, Sabuakham et al. reported a docking score of −7.0 kcal/mol for a potent bis-thiourea inhibitor (compound 4, IC_50_ = 61.63 μM) [24], and Mahalapbutr et al. reported −10.1 kcal/mol for amphotericin B against hsTYR (experimental IC_50_ = 263.36 μM) [17]. The favorable predicted affinities of our hits, especially methotrexate and selinexor, suggest strong potential for potent inhibitory activity. Based on their high predicted binding affinities, good drug-likeness, and commercial availability, these seven compounds were prioritized for experimental validation.

### 3.2. Inhibitory Effects of Candidate Compounds on Mushroom Tyrosinase Activity

**Figure 3:**
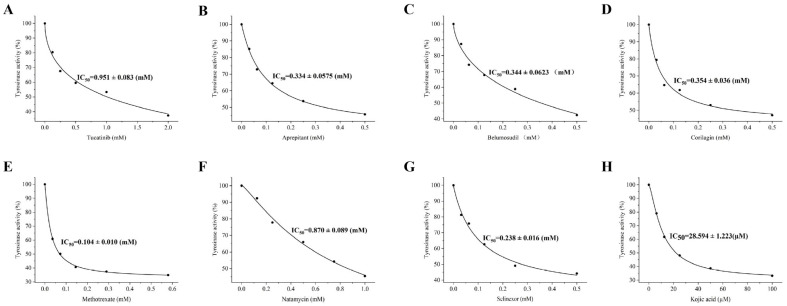
Inhibitory effects of candidate compounds on mushroom tyrosinase activity in vitro. Mushroom tyrosinase was incubated with increasing concentrations of (**A**) tucatinib, (**B**) aprepitant, (**C**) belumosudil, (**D**) corilagin, (**E**) methotrexate, (**F**) natamycin, (**G**) selinexor, and (**H**) kojic acid. Tyrosinase activity was measured using the L-DOPA oxidation assay. Methotrexate and selinexor reduced enzyme activity in a dose-dependent manner, whereas the remaining compounds exhibited only mild inhibition.

The inhibitory effects of the candidate compounds on mushroom tyrosinase were evaluated using L-DOPA as the substrate, with the corresponding dose–response curves illustrated in Figure 3. All tested molecules exhibited concentration-dependent inhibition of tyrosinase activity, with half-maximal inhibitory concentration (IC_50_) values ranging from 0.104 to 0.951 mM. Among the candidates, methotrexate demonstrated the most robust inhibitory potency, yielding an IC_50_ of 0.104 ± 0.011 mM. In contrast, tucatinib was identified as the least potent inhibitor, requiring concentrations approaching 1.0 mM to achieve 50% enzyme inhibition. Although these compounds manifested discernible anti-tyrosinase potential, their molar inhibitory efficacy remained significantly inferior to that of the reference standard, kojic acid (IC_50_ = 28.59 ± 1.22 μM).

This substantial difference in inhibitory efficacy suggests that these compounds do not act through a strong competitive mechanism similar to that of kojic acid. The nitrogen-containing heterocyclic or aromatic moieties within these molecules may non-specifically coordinate with the copper ions in the tyrosinase active site or engage in weak hydrophobic stacking interactions within the substrate-binding pocket, thereby requiring millimolar concentrations to achieve half-maximal inhibition. The observed variations in inhibitory potency among the different compounds (IC_50_ ranging from 0.104 to 0.951 mM) can be attributed to differences in molecular size, charge distribution, or aqueous solubility. For example, methotrexate contains multiple nitrogen atoms and an extended conjugated system, which may facilitate relatively better coordination with the copper centers, whereas tucatinib exhibits the weakest binding likely due to steric hindrance or a lack of suitable coordinating groups. Nevertheless, all tested compounds remain far less effective than the specific tyrosinase inhibitor kojic acid. Therefore, although these candidates demonstrate measurable anti-tyrosinase potential, their clinical applicability for the treatment of hyperpigmentation disorders is limited.

### 3.3. Cytotoxic Profiling and Safety Assessment in MNT-1 Melanoma Cells

**Figure 4:**
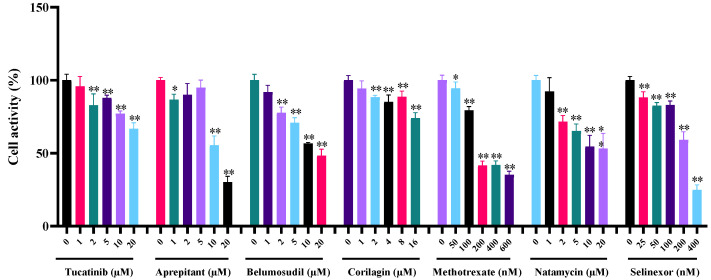
Cytotoxicity of candidate compounds in MNT-1 melanoma cells. Cells were treated with increasing concentrations of tucatinib, aprepitant, belumosudil, corilagin, methotrexate, natamycin, and selinexor for 48 h. Cell viability was assessed by the MTT assay. Methotrexate and selinexor exhibited significant cytotoxicity at relatively low concentrations, whereas the other compounds reduced viability only at higher concentrations. Data are shown as mean ± SEM (*n* = 3). * *p* < 0.05, ** *p* < 0.01 vs. untreated control.

To establish a safety baseline and define optimal dosing for efficacy assays, we evaluated the cytotoxic profiles of the seven hit compounds in MNT-1 human melanoma cells. The results revealed a striking divergence in potency: methotrexate and selinexor exhibited robust antiproliferative effects with IC_50_ values reaching the nanomolar (nM) range, underscoring their high sensitivity in this melanocytic model. In contrast, the remaining five candidates (tucatinib, aprepitant, belumosudil, corilagin, and natamycin) demonstrated significantly lower toxicity, with their inhibitory effects manifesting only at the micromolar (μM) level (Figure 4).

To decouple anti-melanogenic activity from generalized cytotoxicity, we strictly selected sub-cytotoxic concentrations for all subsequent experiments—specifically ≤1 μM for methotrexate and selinexor, and 1–20 μM for the other five compounds. Within these defined therapeutic windows, cell viability consistently remained above 85%, ensuring that any observed reduction in melanin synthesis is attributable to the targeted modulation of the melanogenic pathway rather than a secondary artifact of impaired cell fitness or compromised metabolic activity.

While methotrexate and selinexor exhibit cytotoxic effects at higher concentrations, all anti-melanogenic assessments in this study were conducted at sub-cytotoxic doses (cell viability maintained >90%; Figure 4), ensuring that the observed melanin suppression was not attributable to non-specific cell death. For methotrexate, the cytotoxic IC_50_ in MNT-1 cells is approximately 200 nM, while the highest concentration used in anti-melanogenic assays was 100 nM, yielding a therapeutic window of approximately twofold. Acknowledgedly, this window is modest when considering systemic administration. However, the proposed clinical application for these drugs in hyperpigmentation is topical formulation, which fundamentally alters their pharmacokinetic profile and toxicity risk. Topical methotrexate has been successfully used in dermatology for conditions such as psoriasis and mycosis fungoides, with studies demonstrating negligible systemic bioavailability and favorable local safety when applied to limited skin areas [27,28]. For selinexor, although no commercial topical formulation currently exists, its moderate lipophilicity and molecular weight of 443.3 Da suggest that encapsulation within nanoemulsions or solid lipid nanoparticles could facilitate preferential drug retention in the epidermis, as has been documented for analogous compounds [29,30]. Thus, the narrow therapeutic window under systemic exposure does not preclude topical application, as the effective concentration reaching the basal epidermis can be titrated by formulation design. Future studies will focus on optimizing nanoformulations—such as nanoemulsions, liposomes, or solid lipid nanoparticles—to enhance cutaneous bioavailability while further reducing any potential local irritation and systemic absorption.

### 3.4. Methotrexate and Selinexor Exhibit Superior Inhibition of Cellular Tyrosinase Activity and Melanin Production

**Figure 5:**
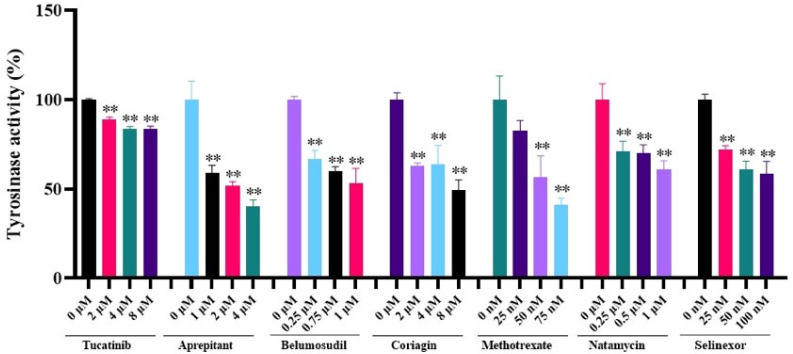
Inhibition of intracellular tyrosinase activity by candidate compounds in MNT-1 cells. Cells were treated with the indicated concentrations of tucatinib, aprepitant, belumosudil, corilagin, methotrexate, natamycin, and selinexor for 48 h. Tyrosinase activity was measured by L-DOPA oxidation. Methotrexate and selinexor dose-dependently reduced enzyme activity, while the other compounds showed only modest inhibition. Data are shown as mean ± SEM (*n* = 3). ** *p* < 0.01 vs. untreated control.

To functionally validate the computational predictions and evaluate the anti-melanogenic potential of the seven hit compounds, we assessed the intracellular tyrosinase activity and total melanin content in MNT-1 human melanoma cells following 48 h exposure to a range of sub-cytotoxic concentrations. All seven candidates elicited a concentration-dependent suppression of cellular tyrosinase activity, yet their inhibitory potencies diverged markedly. The five moderately active agents—tucatinib, aprepitant, belumosudil, corilagin, and natamycin—required micromolar concentrations to achieve appreciable enzymatic inhibition. In stark contrast, methotrexate and selinexor exhibited exceptionally potent activity at nanomolar concentrations, thereby emerging as the lead compounds of this screening effort (Figure 5).

**Figure 6:**
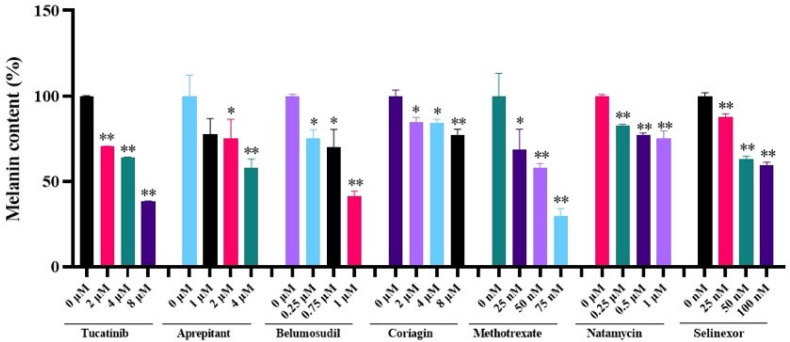
Suppression of melanin content by candidate compounds in MNT-1 cells. Cells were treated with the indicated concentrations of tucatinib, aprepitant, belumosudil, corilagin, methotrexate, natamycin, and selinexor for 48 h. Melanin content was quantified by NaOH lysis. Consistent with tyrosinase activity, methotrexate and selinexor most potently decreased melanin accumulation. Data are shown as mean ± SEM (*n* = 3). * *p* < 0.05, ** *p* < 0.01 vs. untreated control.

Both methotrexate and selinexor triggered robust, dose-dependent suppression of the melanogenic program within the nanomolar range. Treatment with methotrexate produced a dose-dependent decline in enzyme activity, with significant inhibition evident at concentrations as low as 50 nM. Selinexor displayed an even more pronounced inhibitory profile, achieving significant attenuation of tyrosinase function at 25 nM and thereby constituting the most potent inhibitor identified in this study (Figure 5). This nanomolar efficacy represents a 100- to 1000-fold improvement in potency relative to conventional reference compounds such as kojic acid and amphotericin B [17,24]. The pronounced enzymatic suppression translated directly into a marked reduction in intracellular melanin accumulation. At a concentration of 50 nM, methotrexate and selinexor diminished pigment levels to approximately 58.17% and 62.93%, respectively (Figure 6). The strong concordance between tyrosinase inhibition and decreased melanin content confirms that the observed anti-melanogenic effects arise from targeted disruption of the tyrosinase-driven pathway rather than from non-specific cytotoxicity.

These findings substantiate the predictive accuracy of our hsTYR-centered virtual screening strategy and underscore the considerable promise of drug repurposing. Methotrexate and selinexor achieved highly efficacious depigmentation at clinically relevant, low-dose exposures, surpassing the potency of existing inhibitors by several orders of magnitude. However, given their canonical roles as systemic cytotoxins, systemic delivery is precluded for aesthetic indications. Instead, these findings highlight their suitability exclusively for targeted, localized topical application where ultra-low nanomolar efficacy can minimize cutaneous or systemic adverse effects. Although the cellular assays confirm functional activity, the exceptional potency of these compounds at nanomolar concentrations suggests the involvement of additional regulatory mechanisms beyond direct engagement of the enzyme active site, a hypothesis that warrants further mechanistic investigation. Given their well-characterized safety profiles as FDA-approved drugs, methotrexate and selinexor represent highly promising leads for the development of next-generation therapeutics for hyperpigmentation disorders.

### 3.5. Modulation of Intracellular Redox Homeostasis by Methotrexate and Selinexor

Oxidative stress—an imbalance between reactive oxygen species (ROS) production and antioxidant capacity—drives melanogenesis [31]. Excessive ROS from UV, inflammation, or metabolic dysfunction activates the cAMP/PKA/CREB and MAPK pathways, upregulating MITF and promoting melanin synthesis [32]. Enhancing antioxidant defenses suppresses pigmentation, positioning redox modulation as a therapeutic strategy for hyperpigmentation. We therefore examined whether methotrexate and selinexor exert anti-melanogenic effects via redox alterations.

**Figure 7:**
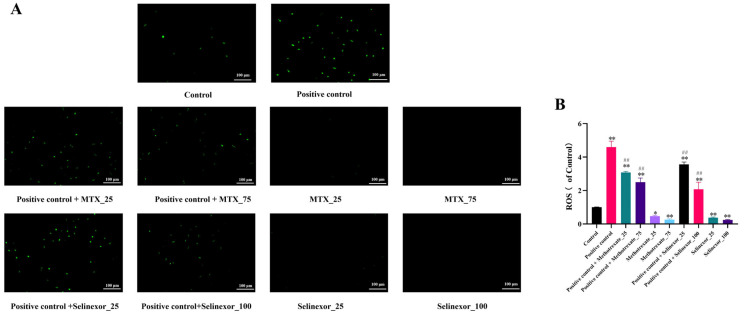
Reduction of intracellular reactive oxygen species (ROS) levels by methotrexate and selinexor in MNT-1 cells. Cells were treated with the indicated concentrations of methotrexate or selinexor for 48 h. ROS levels were measured using DCFH-DA fluorescence (**A**), and quantitative analysis was performed (**B**). Both methotrexate and selinexor significantly decreased ROS production in a concentration-dependent manner. Data are shown as mean ± SEM (*n* = 3). * *p* < 0.05, ** *p* < 0.01 vs. untreated control. ## *p* < 0.01 vs. Positive control.

Intracellular ROS in MNT-1 cells was measured using DCFH-DA after 48 h treatment. Both agents induced concentration-dependent reductions in basal ROS. At 75 nM, methotrexate reduced ROS to 28.88%. At 100 nM, selinexor reduced ROS to 23.96% (Figure 7). Fluorescence microscopy confirmed attenuated DCF staining, indicating a globally quiescent oxidative state. These antioxidant effects match or exceed those of N-acetylcysteine and ascorbic acid in melanocyte models [33].

**Figure 8:**
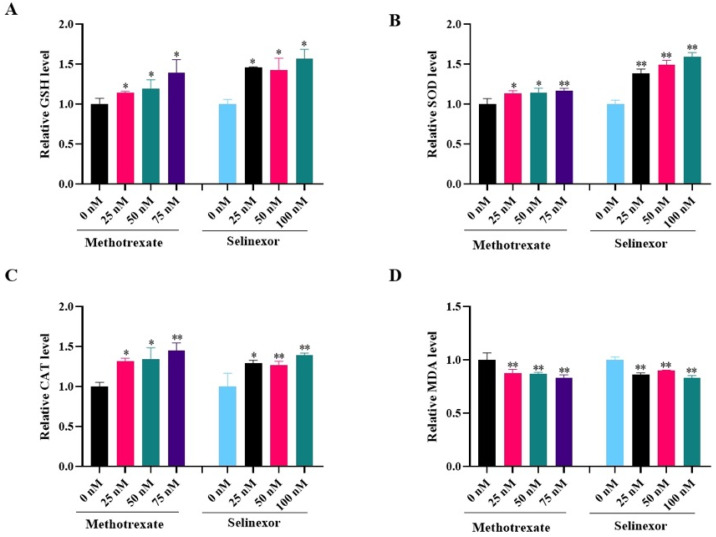
Enhancement of the intracellular antioxidant defense system by methotrexate and selinexor in MNT-1 cells. Cells were treated with increasing concentrations of methotrexate or selinexor for 48 h. (**A**) Levels of reduced glutathione (GSH). (**B**) Activity of superoxide dismutase (SOD). (**C**) Activity of catalase (CAT). (**D**) Levels of malondialdehyde (MDA). Methotrexate and selinexor significantly elevated GSH, SOD, and CAT while reducing MDA, indicating attenuation of oxidative stress and lipid peroxidation. Data are shown as mean ± SEM (*n* = 3). * *p* < 0.05, ** *p* < 0.01 vs. untreated control.

To elucidate mechanisms, we assessed key antioxidant defenses. Methotrexate dose-dependently elevated reduced glutathione (GSH) levels: 114.51%, 119.25%, and 139.00% at 25, 50, and 75 nM, respectively. Selinexor similarly increased GSH by 145.65%,142.88%, and 156.53% at 25, 50, and 100 nM (Figure 8A). GSH restoration is functionally significant because GSH depletion exacerbates melanogenesis via p38 MAPK activation.

Enzymatic activities of superoxide dismutase (SOD) and catalase (CAT) were also augmented. Methotrexate increased SOD activity by 113.17%, 114.66%, and 116.59% and boosted CAT activity up to 145.03% at 75 nM. Selinexor enhanced SOD and CAT activities by up to 159.13% and 139.56% at 100 nM (Figure 8B,C). This coordinated upregulation facilitates superoxide dismutation and hydrogen peroxide elimination, interrupting ROS-driven pro-melanogenic signaling.

Consistent with enhanced antioxidant capacity, malondialdehyde (MDA), a lipid peroxidation end-product and oxidative damage marker, was substantially reduced. Methotrexate (75 nM) decreased MDA to 83.50%. Selinexor (100 nM) reduced MDA to 83.36% (Figure 8D). This attenuation of oxidative membrane injury underscores functional relevance.

These findings align with evidence implicating oxidative stress in pathological pigmentation and the clinical benefit of antioxidants in melasma. Notably, methotrexate and selinexor surpass other tyrosinase inhibitors: amphotericin B does not alter ROS levels, and bis-thiourea derivatives lack explored antioxidant effects. The pronounced enhancement of the GSH–SOD–CAT axis distinguishes these agents, integrating direct tyrosinase inhibition with comprehensive redox remodeling.

Collectively, methotrexate and selinexor profoundly reconfigure the redox landscape of MNT-1 cells. By reducing basal ROS, bolstering GSH, augmenting SOD and CAT activities, and curbing lipid peroxidation, these compounds create a reductive cellular milieu less permissive to pro-pigmentation signaling. This global antioxidant effect complements direct tyrosinase suppression and MITF regulation, providing a molecular foundation for their exceptional anti-melanogenic potency.

### 3.6. Transcriptional Suppression of the MITF/TYR Axis Is a Primary Mechanism of Action

To elucidate the molecular basis for the potent anti-melanogenic activity of methotrexate and selinexor, we examined their effects on the transcriptional program governing melanin synthesis. Given that microphthalmia-associated transcription factor (MITF) is the master regulator of pigmentation, MNT-1 cells were treated with escalating concentrations of each compound for 48 h, and mRNA levels of MITF and its canonical downstream targets, TYR, TRP1, and TRP2, were quantified by qRT-PCR using β-actin as an internal control.

**Figure 9:**
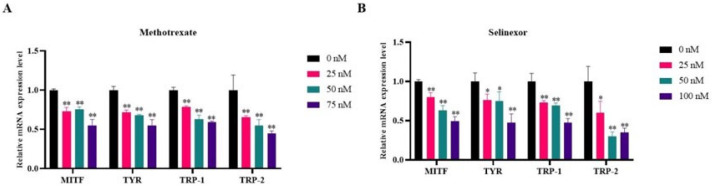
Downregulation of melanogenesis-related gene transcription by methotrexate and selinexor in MNT-1 cells. Cells were treated with increasing concentrations of (**A**) methotrexate or (**B**) selinexor for 48 h. mRNA levels of MITF, TYR, TRP1, and TRP2 were quantified by qRT-PCR and normalized to β-actin. Both drugs concentration-dependently suppressed the transcript levels of all four genes. Data are shown as mean ± SEM (*n* = 3). * *p* < 0.05, ** *p* < 0.01 vs. untreated control.

Both agents induced marked, concentration-dependent reductions in MITF transcript abundance. Methotrexate at 25, 50, and 75 nM suppressed MITF mRNA to 73.09%, 75.66%, and 55.00% (Figure 9A), respectively, whereas selinexor at 25, 50, and 100 nM suppressed MITF mRNA to 79.79%, 63.26%, and 49.45% (Figure 9B). Notably, the degree of MITF repression at nanomolar concentrations was comparable to that reported for conventional inhibitors such as arbutin at millimolar doses. This downregulation was accompanied by coordinate, dose-dependent declines in downstream targets. After treatment with methotrexate (25–75 nM), TYR mRNA was suppressed to 71.54%, 68.03%, and 54.58%; after treatment with selinexor (25–100 nM), TYR mRNA was suppressed to 76.00%, 75.07%, and 47.59%. TRP1 and TRP2 transcripts were similarly attenuated, with maximal suppression to 59.46% and 44.59% at 75 nM methotrexate, and to 47.46% and 34.77% at 100 nM selinexor. The parallel suppression of these four core melanogenic genes indicates that methotrexate and selinexor act at the apex of the transcriptional hierarchy, targeting MITF as the primary regulatory node rather than modulating each downstream effector independently.

**Figure 10:**
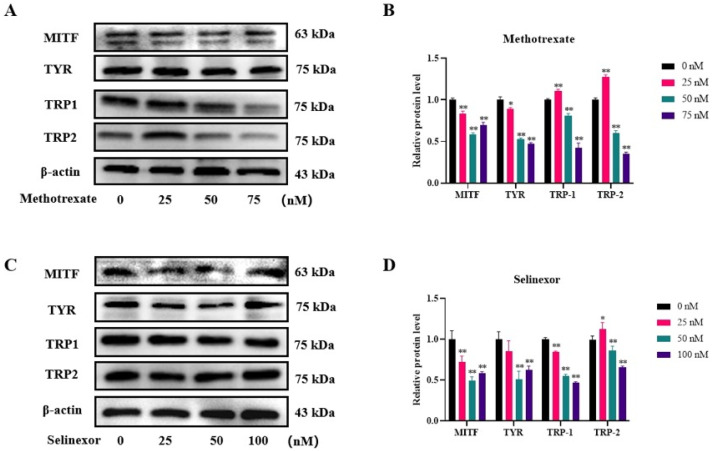
Suppression of key melanogenic protein expression by methotrexate and selinexor in MNT-1 cells. Cells were treated with increasing concentrations of methotrexate or selinexor for 48 h. Protein expression levels of MITF, TYR, TRP1, and TRP2 after methotrexate and selinexor treatment were assessed by Western blotting (**A**,**C**), and the densitometric quantification data are presented in (**B**,**D**). Consistent with mRNA data, both drugs dose-dependently reduced the protein expression of MITF and its downstream effectors. Data are shown as mean ± SEM (*n* = 3). * *p* < 0.05, ** *p* < 0.01 vs. untreated control.

Immunoblotting confirmed that transcriptional suppression translated to the protein level (Figure 10). Densitometric analysis revealed that methotrexate (25, 50, 75 nM) reduced MITF protein levels to 83.20%, 58.75%, and 70.08%, respectively, while selinexor (25, 50, 100 nM) reduced them by 72.27%, 49.81%, and 58.60%. TYR, TRP1, and TRP2 proteins exhibited parallel dose-dependent declines. The strong positive correlation between MITF mRNA and protein abundance indicates that transcriptional repression is the primary mechanism by which these agents dismantle the melanogenic program.

Collectively, these findings establish profound suppression of the MITF/TYR transcriptional axis as a central mechanism of action for methotrexate and selinexor. By potently downregulating MITF at both mRNA and protein levels, these compounds orchestrate a coordinated collapse of melanogenic gene expression, thereby depleting the key enzymes TYR, TRP1, and TRP2 required for melanin synthesis. This transcriptional mode of action operates in concert with the direct inhibition of tyrosinase catalytic activity and restoration of intracellular redox homeostasis, together accounting for the exceptional, multi-faceted anti-melanogenic efficacy of these repurposed FDA-approved agents.

### 3.7. Dual-Pathway Inhibition of MITF Transcription via CREB and β-Catenin

MITF transcription is cooperatively regulated by two principal signaling cascades: the cAMP/PKA/CREB axis and the Wnt/β-catenin pathway. α-MSH binding to MC1R activates cAMP/PKA, leading to CREB phosphorylation at Ser133 and recruitment of CBP/p300 to drive MITF expression [34]. Concurrently, the canonical Wnt pathway promotes MITF transcription via nuclear translocation of β-catenin, which forms a transcriptional complex with TCF/LEF at the MITF promoter [35]. Given that methotrexate and selinexor markedly reduce MITF mRNA and protein levels, we hypothesized that these agents interfere with one or both upstream cascades.

**Figure 11:**
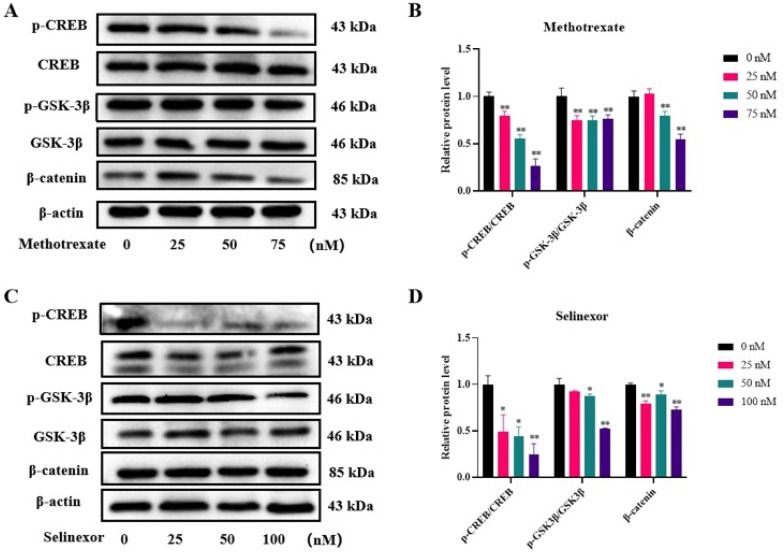
Inhibition of the cAMP/PKA/CREB and Wnt/β-catenin signaling pathways by methotrexate and selinexor in MNT-1 cells. Cells were treated with increasing concentrations of methotrexate or selinexor for 48 h. Protein expression levels of phosphorylated CREB (Ser133), total CREB, total β-catenin, phosphorylation of GSK3β (Ser9), and total GSK3β after methotrexate and selinexor treatment were assessed by Western blotting (**A**,**C**), and the densitometric quantification data are presented in (**B**,**D**). Western blot analysis revealed a dose-dependent reduction in phosphorylated CREB (Ser133) and total β-catenin, accompanied by decreased inhibitory phosphorylation of GSK3β (Ser9), indicating enhanced GSK3β activity and consequent β-catenin degradation. Total CREB and GSK3β levels remained unchanged. Data are shown as mean ± SEM (*n* = 3). * *p* < 0.05, ** *p* < 0.01 vs. untreated control.

To test this, MNT-1 cells were treated with methotrexate or selinexor for 48 h, and CREB phosphorylation at Ser133 was assessed using a phospho-specific antibody. Both compounds induced a concentration-dependent suppression of CREB phosphorylation. At 25, 50, and 75 nM, methotrexate reduced p-CREB levels to 79.78%, 55.38%, and 26.07%, respectively (Figure 11A); selinexor at 25, 50, and 100 nM reduced p-CREB by 48.77%, 44.25%, and 24.43% (Figure 11B). Total CREB protein remained unchanged, indicating that the decline in p-CREB stems from impaired phosphorylation rather than altered CREB expression or stability.

We next examined the impact on the Wnt/β-catenin pathway. GSK3β is a constitutively active kinase that phosphorylates β-catenin at Ser33, Ser37, and Thr41, targeting it for proteasomal degradation [36]. GSK3β activity is negatively regulated by phosphorylation at Ser9; thus, elevated p-GSK3β (Ser9) indicates GSK3β inactivation and β-catenin stabilization. Western blot analysis revealed that both methotrexate and selinexor induced a concentration-dependent decrease in inhibitory p-GSK3β (Ser9). Methotrexate at 75 nM reduced p-GSK3β (Ser9) to 76.16% (Figure 11A), while selinexor at 100 nM reduced it to 51.94% (Figure 11B); total GSK3β levels remained constant. Decreased p-GSK3β (Ser9) implies enhanced GSK3β activity, which should accelerate β-catenin phosphorylation and degradation. Indeed, total β-catenin abundance was significantly diminished: methotrexate at 75 nM reduced β-catenin to 51.94% (Figure 11A), and selinexor at 100 nM reduced it to 73.12% (Figure 11B). These data indicate that methotrexate and selinexor activate GSK3β by attenuating its inhibitory Ser9 phosphorylation, thereby promoting β-catenin turnover and depleting the nuclear pool of this essential MITF co-activator.

The dual inhibition of the cAMP/PKA/CREB and Wnt/β-catenin pathways provides a compelling mechanistic rationale for the profound downregulation of MITF. By concurrently attenuating CREB-driven activation and promoting β-catenin degradation, these agents dismantle the two principal signaling inputs that sustain MITF expression. Such a two-pronged strategy is inherently more robust and less susceptible to compensatory feedback than single-pathway intervention. This dual mechanism further distinguishes methotrexate and selinexor from most tyrosinase inhibitors, which target only a single signaling axis. For instance, bis-thiourea derivatives directly inhibit tyrosinase and chelate copper but have unexplored effects on upstream signaling. Natural products such as kojic acid and arbutin function primarily as direct tyrosinase inhibitors with minimal impact on MITF transcription. The capacity of methotrexate and selinexor to simultaneously engage multiple signaling nodes—CREB phosphorylation and GSK3β/β-catenin regulation—likely accounts for their exceptional nanomolar potency and enables more comprehensive suppression of the melanogenic program.

Collectively, these findings demonstrate that methotrexate and selinexor suppress MITF transcription via a dual-pathway mechanism: they inhibit CREB phosphorylation to block the cAMP/PKA/CREB axis while concurrently activating GSK3β to promote β-catenin degradation and restrain the Wnt/β-catenin pathway. This dual transcriptional blockade acts in concert with direct tyrosinase inhibition, restoration of redox homeostasis, and post-translational MITF degradation, together establishing the molecular foundation for the potent, multi-faceted anti-melanogenic activity of these repurposed drug candidates.

### 3.8. Post-Translational Regulation: AKT/ERK Activation Promotes MITF Degradation

In addition to transcriptional regulation, the stability of MITF protein is finely controlled by post-translational modifications. AKT and ERK phosphorylate distinct serine residues on MITF to generate a phospho-degron, which is recognized by the E3 ubiquitin ligase β-TrCP and then targets MITF for proteasomal degradation [37]. Given that the reduction in MITF protein levels induced by methotrexate and selinexor was greater than the reduction in MITF mRNA, we hypothesized that these two agents also accelerate MITF clearance via post-translational mechanisms.

**Figure 12:**
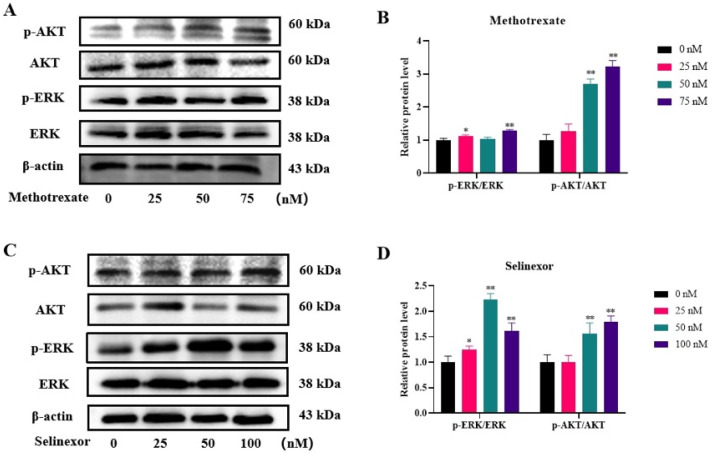
Activation of the AKT and ERK signaling pathways by methotrexate and selinexor in MNT-1 cells. Cells were treated with increasing concentrations of methotrexate or selinexor for 48 h. Protein expression levels of phosphorylated AKT (Ser473), total AKT, phosphorylation of ERK (Thr202/Tyr204), and total ERK after methotrexate and selinexor treatment were assessed by Western blotting (**A**,**C**), and the densitometric quantification data are presented in (**B**,**D**). Western blot analysis showed significant increases in phosphorylation of AKT (Ser473) and ERK (Thr202/Tyr204), while total MITF protein decreased concurrently. These results suggest that drug-induced AKT/ERK activation promotes proteasomal degradation of MITF. Data are shown as mean ± SEM (*n* = 3). * *p* < 0.05, ** *p* < 0.01 vs. untreated control.

To test this hypothesis, MNT-1 cells were treated with increasing concentrations of methotrexate or selinexor for 48 h, followed by Western blotting using antibodies that specifically recognize the activating phosphorylation sites p-AKT (Ser473) and p-ERK (Thr202/Tyr204); total AKT and total ERK served as loading controls (Figure 12). Both agents markedly enhanced AKT and ERK phosphorylation in a concentration-dependent manner. Methotrexate at 75 nM increased p-AKT (Ser473) levels to 321.69%, respectively, and elevated p-ERK levels to 128.36% (Figure 12A). Selinexor at 100 nM increased p-AKT and p-ERK to 170.95% and 161.57%, respectively (Figure 12B). Total AKT and total ERK levels remained unchanged under all treatment conditions, indicating that the observed effects resulted from enhanced phosphorylation rather than increased kinase expression.

AKT/ERK-driven MITF degradation has been reported for other anti-melanogenic agents, such as ginsenoside Re and amphotericin B [17,38]. The degrees of AKT/ERK activation and MITF degradation achieved by methotrexate and selinexor are comparable to, or even greater than, those observed with these reference compounds, underscoring the exceptional potency of methotrexate and selinexor. Of note, the activation of AKT and ERK by methotrexate and selinexor may be cell-context-dependent and likely diverges from their canonical anticancer mechanisms (DHFR inhibition for methotrexate; XPO1 inhibition for selinexor) [39,40]. The observed kinase activation may reflect off-target effects or compensatory stress responses triggered by metabolic disturbances or altered nucleocytoplasmic transport. Elucidating the upstream mediators will require future kinome profiling studies.

In summary, methotrexate and selinexor synergistically suppress MITF through a dual mechanism involving transcriptional inhibition and accelerated proteasomal degradation. By simultaneously reducing the synthesis of nascent MITF and promoting the clearance of pre-existing MITF, these two agents achieve rapid and sustained depletion of this master transcription factor, leading to the collapse of the melanogenic transcriptional network. This multi-pronged strategy—targeting MITF at both the levels of synthesis and stability—is inherently more effective than interventions aimed at a single node (e.g., transcription alone or enzymatic activity alone). The present findings establish AKT/ERK-mediated post-translational regulation as an integral component of the anti-melanogenic mechanism of methotrexate and selinexor. By activating AKT and ERK, these drugs enhance phosphorylation-dependent MITF degradation, an effect that acts in concert with transcriptional repression achieved through the cAMP/PKA/CREB and Wnt/β-catenin pathways. This dual attack on MITF production and persistence distinguishes methotrexate and selinexor from conventional tyrosinase inhibitors and provides a robust molecular foundation for their therapeutic potential in hyperpigmentation disorders.

### 3.9. MITF Overexpression Rescues Drug-Induced Inhibition, Confirming Its Central Role

The preceding sections have demonstrated that methotrexate and selinexor inhibit melanogenesis through a multifaceted mechanism that includes direct tyrosinase inhibition, restoration of redox homeostasis, dual suppression of the MITF/TYR transcriptional axis via CREB and β-catenin, and enhanced MITF proteasomal degradation through AKT/ERK signaling. Although MITF emerges as the central regulatory node targeted by these agents, it remains unclear whether MITF suppression is the primary cause of their anti-melanogenic effects or a secondary downstream consequence. To obtain definitive genetic evidence that MITF is the principal functional target, we performed rescue experiments by overexpressing the melanocyte-specific MITF-M isoform in MNT-1 cells and assessing whether enforced MITF expression could override methotrexate- and selinexor-induced inhibition.

**Figure 13:**
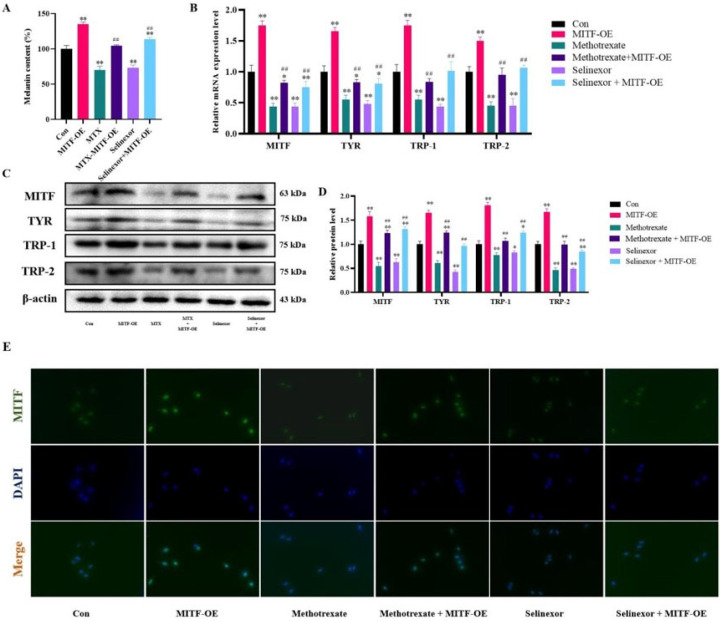
Overexpression of MITF-M reverses the inhibitory effects of methotrexate and selinexor on melanogenesis in MNT-1 cells. Cells were transfected with MITF-M overexpression plasmid (MITF-M-OE) or empty vector, followed by treatment with methotrexate or selinexor for 48 h. (**A**) Melanin content was measured by NaOH lysis. (**B**–**D**) mRNA (**B**) and protein (**C**,**D**) expression levels of TYR, TRP1, and TRP2 were assessed by qRT-PCR and Western blot, respectively. (**E**) Representative immunofluorescence images showing nuclear MITF localization (green) and DAPI counterstaining (blue); scale bar = 20 μm. Forced MITF-M expression significantly rescued melanin synthesis, restored downstream enzyme expression, and recovered nuclear MITF signal intensity diminished by drug treatment. Data are shown as mean ± SEM (*n* = 3). * *p* < 0.05, ** *p* < 0.01 vs. control + vector; ## *p* < 0.01 vs. drug-treated + vector.

The MITF gene produces multiple isoforms through alternative promoter usage and splicing. Among them, the MITF-M isoform is exclusively expressed in melanocytes and melanoma cells and contains a unique N-terminal domain essential for its transcriptional activity and melanocyte-specific functions [41]. We cloned the full-length human MITF-M coding sequence into the pcDNA3.1(+) vector to generate a MITF-M overexpression plasmid (MITF-M-OE). MNT-1 cells were transiently transfected with MITF-M-OE and subsequently treated with methotrexate (75 nM) or selinexor (100 nM) for 48 h. Melanin content, gene and protein expression, and MITF subcellular localization were then evaluated. Enforced MITF-M expression completely abrogated the anti-melanogenic effects of both drugs. Compared with control cells, methotrexate and selinexor reduced intracellular melanin content to 70.28% and 73.24%, respectively. In MITF-M-OE-transfected cells, melanin levels in the presence of methotrexate or selinexor were restored to 104.70% and 113.63% of control levels, respectively, indicating near-complete reversal of drug-induced inhibition and confirming functional rescue by MITF overexpression (Figure 13A).

Restoration of melanin synthesis was accompanied by recovery of the downstream melanogenic gene expression program. qRT-PCR analysis showed that methotrexate and selinexor suppressed mRNA levels of MITF, TYR, TRP1, and TRP2 to 43.79%, 55.10%, 54.84%, and 45.37% (methotrexate) and to 44.21%, 47.96%, 43.81%, and 46.89% (selinexor) of control levels. In MITF-M-OE-transfected cells exposed to the drugs, transcript levels of these genes were restored to 82.07%, 82.99%, 83.95%, and 94.87X% (methotrexate) and to 75.16%, 80.95%, 101.34%, and 106.39% (selinexor) of control levels (Figure 13B). Thus, exogenous MITF-M fully drives the expression of its canonical targets even under drug treatment.

Western blot analysis corroborated these findings at the protein level. In vector-transfected cells, methotrexate and selinexor reduced MITF, TYR, TRP1, and TRP2 protein abundance, whereas MITF-M overexpression restored their levels (Figure 13C,D); densitometric quantification confirmed the reversal of drug-induced suppression. Notably, exogenous MITF-M protein resisted drug-induced degradation, likely because supraphysiological expression achieved by transient transfection exceeded the degradative capacity of the AKT/ERK-proteasome machinery.

Immunofluorescence staining and confocal microscopy further validated functional rescue and assessed MITF subcellular localization. In control cells, MITF exhibited prominent nuclear distribution, consistent with its transcriptional role (Figure 13E). In MITF-M-OE-transfected cells, nuclear MITF signal was robustly restored irrespective of drug treatment. These imaging data provide direct visual evidence that exogenous MITF-M efficiently translocates to the nucleus and functionally replaces the endogenous MITF pool depleted by drug exposure.

The genetic rescue strategy employed herein represents a powerful and widely accepted approach for establishing causal relationships. Similar rescue experiments have validated MITF as a central regulator of melanogenesis. For example, Chu et al. demonstrated that MITF overexpression reversed the anti-melanogenic effects of 1,2,3,4,6-pentagalloylglucose in B16F10 cells [42], and Hwang et al. showed that MITF knockdown abolished the melanogenic effects of ginsenoside Re [38]. In the present study, complete rescue of melanin synthesis by MITF-M overexpression in the presence of methotrexate and selinexor provides equally compelling genetic evidence that MITF is the principal downstream effector of these drugs.

The ability of MITF-M overexpression to overcome drug-induced inhibition also carries mechanistic implications. It demonstrates that upstream signaling events targeted by methotrexate and selinexor, including CREB phosphorylation, β-catenin stabilization, and AKT/ERK activation, converge on MITF as a critical regulatory bottleneck. Bypassing these upstream signals via direct provision of exogenous MITF-M renders cells refractory to drug treatment, confirming that the drugs do not irreversibly damage the core melanogenic machinery but instead deplete the essential transcription factor required for its expression. This finding aligns with the multi-pathway mechanism elucidated earlier and reinforces MITF as the “Achilles’ heel” of the melanogenic program.

In summary, genetic rescue experiments provide definitive evidence that MITF is the indispensable mediator of the anti-melanogenic effects of methotrexate and selinexor. Enforced expression of the melanocyte-specific MITF-M isoform completely abrogates drug-induced inhibition of melanin synthesis, restores mRNA, and protein expression of downstream melanogenic enzymes, and replenishes the nuclear MITF pool. This genetic complementation unequivocally establishes MITF as the principal functional target and validates the mechanistic model proposed in this study: methotrexate and selinexor suppress melanogenesis by orchestrating a coordinated, multi-pronged attack on MITF at both the transcriptional and post-translational levels.

Addressing the mechanistic coherence of multi-pathway regulation. A potential concern is that methotrexate and selinexor, initially identified by docking-based screening against human tyrosinase, also modulate seemingly unrelated upstream signaling cascades (cAMP/PKA/CREB, Wnt/β-catenin, AKT/ERK). This observation, however, is not contradictory; it reflects the well-established principle of drug polypharmacology—the ability of a single small molecule to interact with multiple protein targets and modulate distinct nodes within a biological network. In melanogenesis, MITF expression and stability are jointly governed by transcriptional inputs (cAMP/PKA/CREB and Wnt/β-catenin) and post-translational degradation signals (AKT/ERK). Thus, a compound that engages distinct components of this regulatory network can produce coordinated effects at multiple levels. Such multi-pathway regulation of melanogenesis by a single agent is not unprecedented. For example, decursin suppresses melanin synthesis via concurrent regulation of PKA/CREB, p38, ERK and PI3K/Akt/GSK-3β cascades, promoting MITF degradation [43]. PAK4 regulates melanogenesis through the simultaneous activation of both the CREB/MITF and β-catenin/MITF pathways [44]. HDAC inhibitors suppress MITF expression by interfering with lineage-restricted transcriptional mechanisms without directly targeting tyrosinase [45]. Collectively, these examples support that coordinated multi-pathway modulation of MITF is a viable anti-melanogenic strategy.

What distinguishes methotrexate and selinexor is the combination of three features: (i) high predicted binding affinity to tyrosinase; (ii) direct inhibition of tyrosinase enzymatic activity in cell-free assays (albeit with lower potency than kojic acid); and (iii) nanomolar efficacy in suppressing melanogenesis in intact cells, which far exceeds what could be explained by direct tyrosinase inhibition alone. This potency gap suggests that in MNT-1 cells, the anti-melanogenic activity arises primarily from MITF downregulation, while direct tyrosinase inhibition plays a secondary, contributory role. The docking-based screen served as a discovery tool; the functional mechanism in the cellular context is more complex and involves multi-pathway crosstalk. Notably, the genetic rescue experiments provide definitive evidence that MITF is the critical downstream effector node: enforced expression of MITF-M completely overrides drug-induced inhibition, restoring melanin synthesis to the control levels. Thus, the multiple signaling changes induced by methotrexate and selinexor converge on MITF as the central regulatory bottleneck, establishing a coherent mechanistic framework that reconciles the observed multi-pathway effects with the primary findings of this study.

## 4. Conclusions

**Figure 14:**
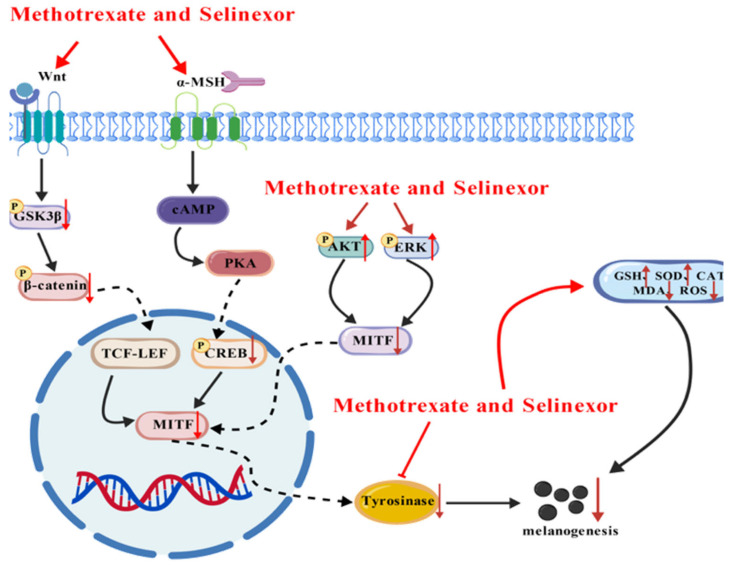
Proposed model of the multi-pathway inhibitory mechanism of methotrexate and selinexor on melanogenesis. Methotrexate and selinexor suppress melanin synthesis through convergent regulation of MITF at both the transcriptional and post-translational levels. They inhibit the cAMP/PKA/CREB and Wnt/β-catenin signaling pathways, reducing MITF transcription. Concurrently, they activate the AKT/ERK pathways, promoting ubiquitin-proteasome-mediated degradation of MITF. In parallel, both drugs enhance cellular antioxidant capacity by upregulating GSH, SOD, and CAT and reducing ROS and MDA, thereby removing oxidative stress-driven pigmentation signals. This multi-pronged attack on the melanogenic machinery leads to potent suppression of tyrosinase activity and melanin synthesis.

In this study, we successfully integrated computational drug discovery with comprehensive experimental validation to identify methotrexate and selinexor as novel, potent anti-melanogenic agents. Our findings reveal a sophisticated multi-pathway mechanism of action converging on the master transcription factor MITF. Both drugs orchestrate a coordinated attack on melanogenesis by: (i) directly or indirectly inhibiting tyrosinase catalytic function; (ii) remodeling the intracellular redox environment to reduce pro-pigmentation oxidative signals; (iii) suppressing MITF transcription by inhibiting the cAMP/PKA/CREB axis and promoting β-catenin degradation; and (iv) accelerating MITF protein clearance via activation of the AKT/ERK-proteasome pathway (Figure 14). The central role of MITF was definitively confirmed by genetic rescue experiments. Given their status as FDA-approved drugs with well-characterized safety profiles, methotrexate and selinexor warrant further investigation as potential candidates for drug repurposing in hyperpigmentation disorders. However, given their known systemic toxicities, any future clinical application would be contingent upon the successful development of optimized topical formulations (e.g., nanoemulsions, liposomes, or hydrogels) that can restrict drug activity to the epidermis while minimizing systemic exposure. Extensive preclinical safety and toxicology studies using these formulations are required before clinical translation can be considered. Future studies will focus on validating these effects in vivo using 3D skin equivalents and animal models, and on developing optimized topical formulations to enhance cutaneous bioavailability.

## Data Availability

The original contributions presented in this study are included in the article/Supplementary Materials. Further inquiries can be directed to the corresponding authors.

## Supplementary Materials

The following supporting information can be downloaded at: https://www.mdpi.com/article/10.3390/cells15121070/s1. Original Western Blotting Figure. Table S1: Information related to the antibodies used. Table S2: Primers of sequences for qRT-PCR analysis. Table S3: Information related to the MITF-M plasmid. Table S4: Information on the compounds from virtual screening and their binding energies after docking. Table S5: Candidate compound profiles and their corresponding binding affinities following molecular docking.
