# Supplementary figures and images for: Structure-Based Virtual Screening and Mechanistic Characterization of Methotrexate and Selinexor as Potent Anti-Melanogenic Agents via Multi-Pathway Suppression of MITF
Source: Cells. 2026 Jun 11;15(12):1070. doi: 10.3390/cells15121070 (PMC13296867; doi:10.3390/cells15121070)

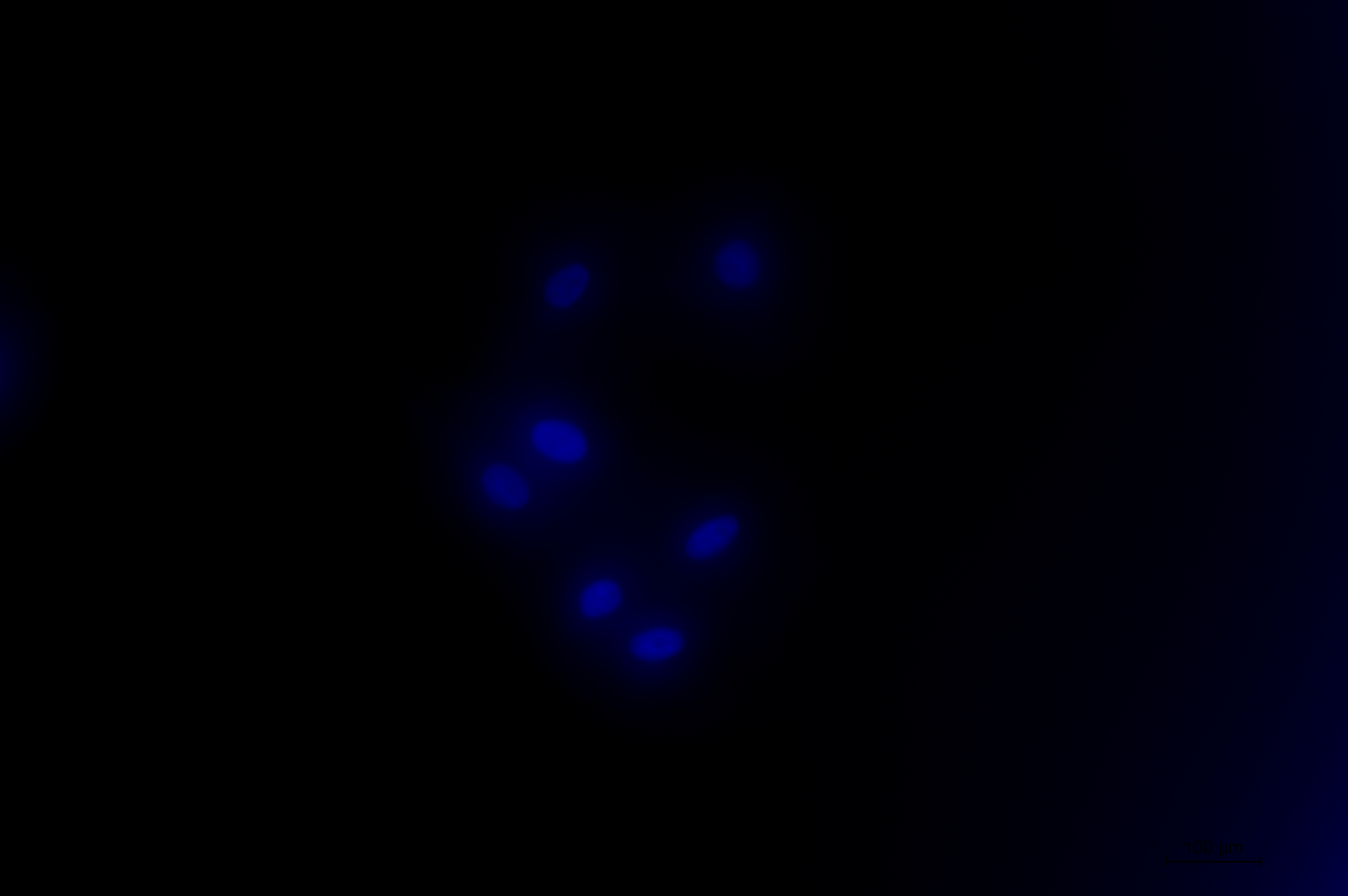

Supplement: Supplementary file 1 [file cells-15-01070-s001.zip › Supplementary File/Orginal image/Figure 14E_DAPI_Con.png]

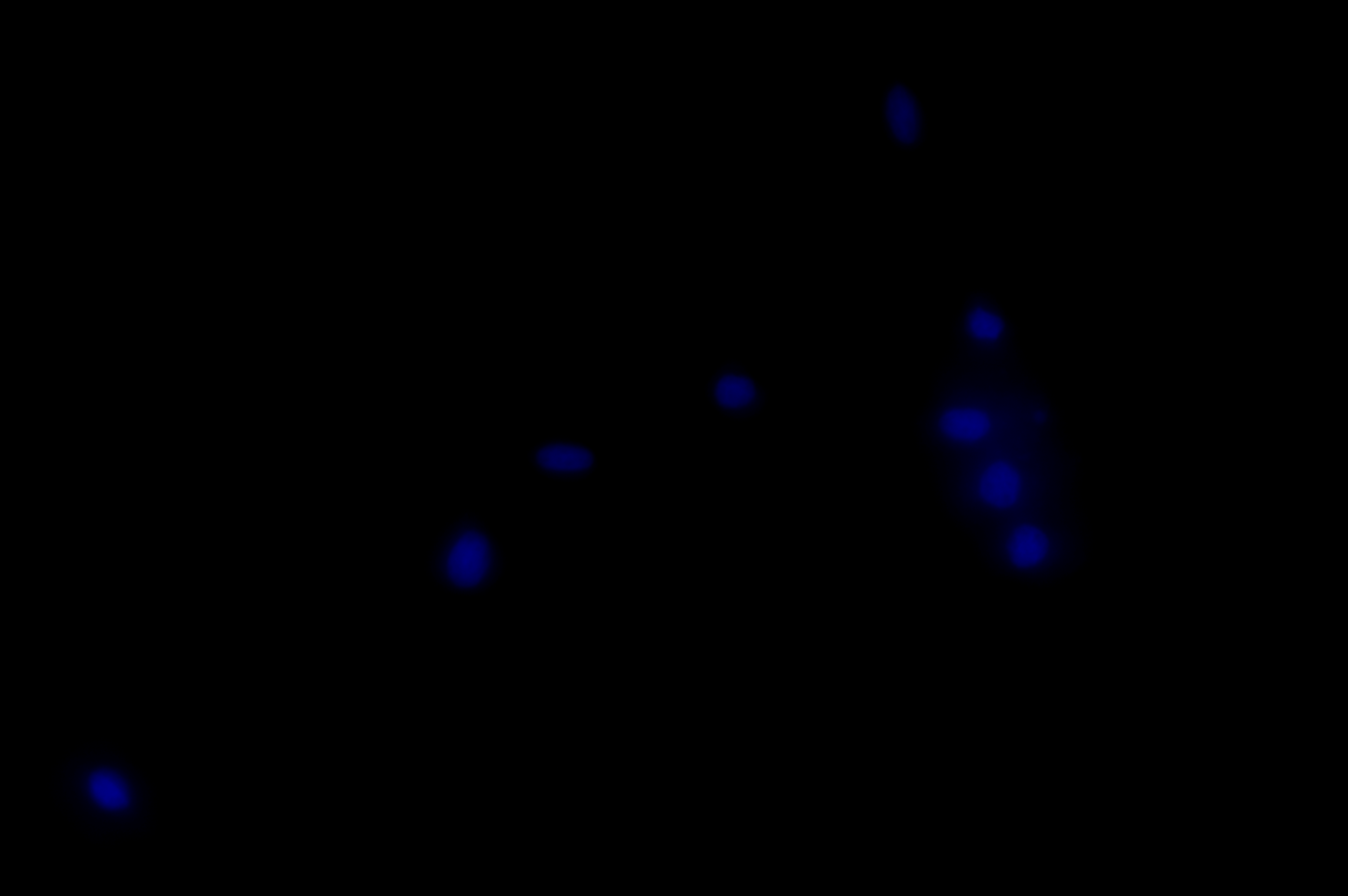

Supplement: Supplementary file 1 [file cells-15-01070-s001.zip › Supplementary File/Orginal image/Figure 14E_DAPI_Methotrexate+MITF-OE.png]

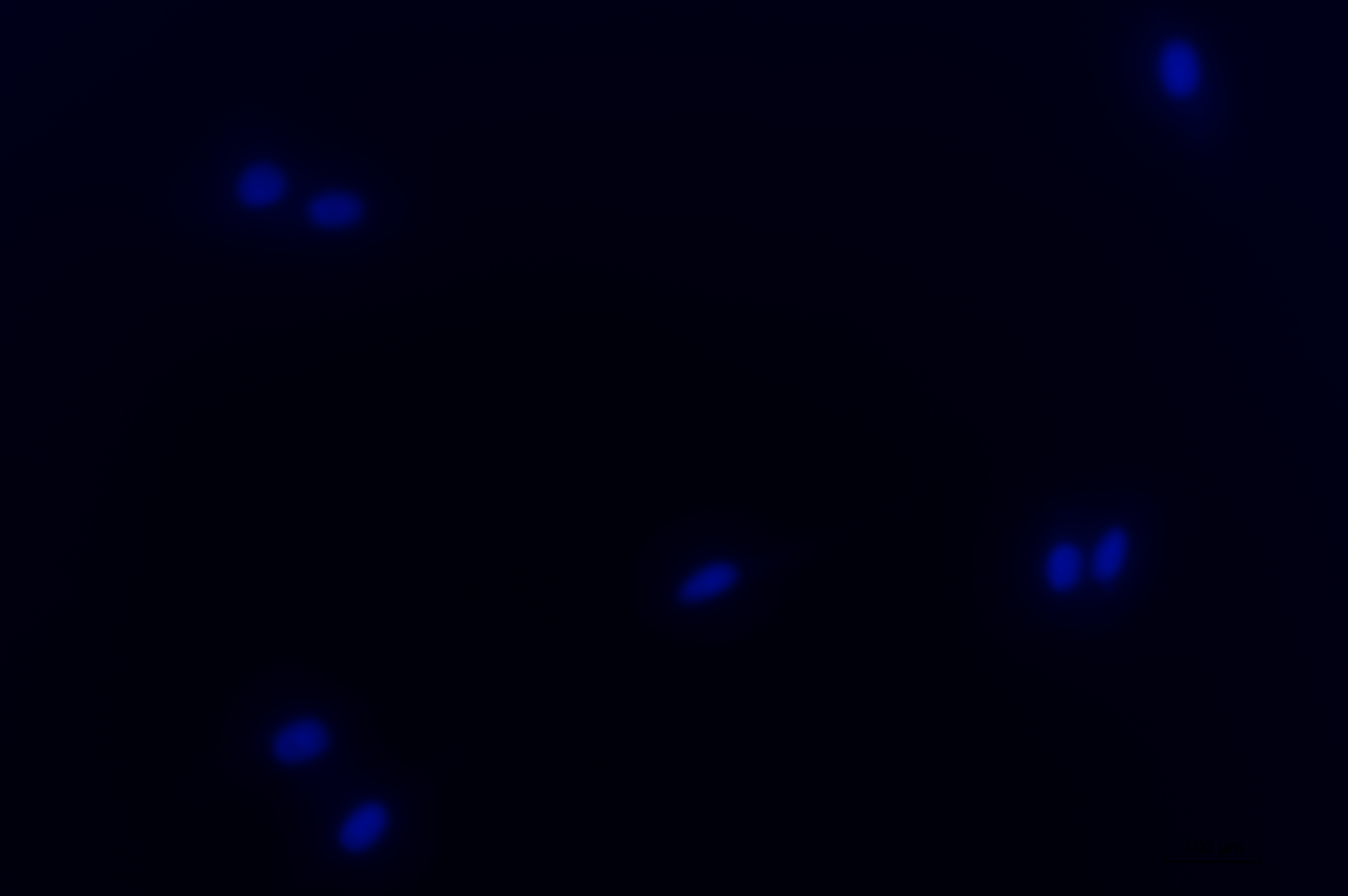

Supplement: Supplementary file 1 [file cells-15-01070-s001.zip › Supplementary File/Orginal image/Figure 14E_DAPI_Methotrexate.png]

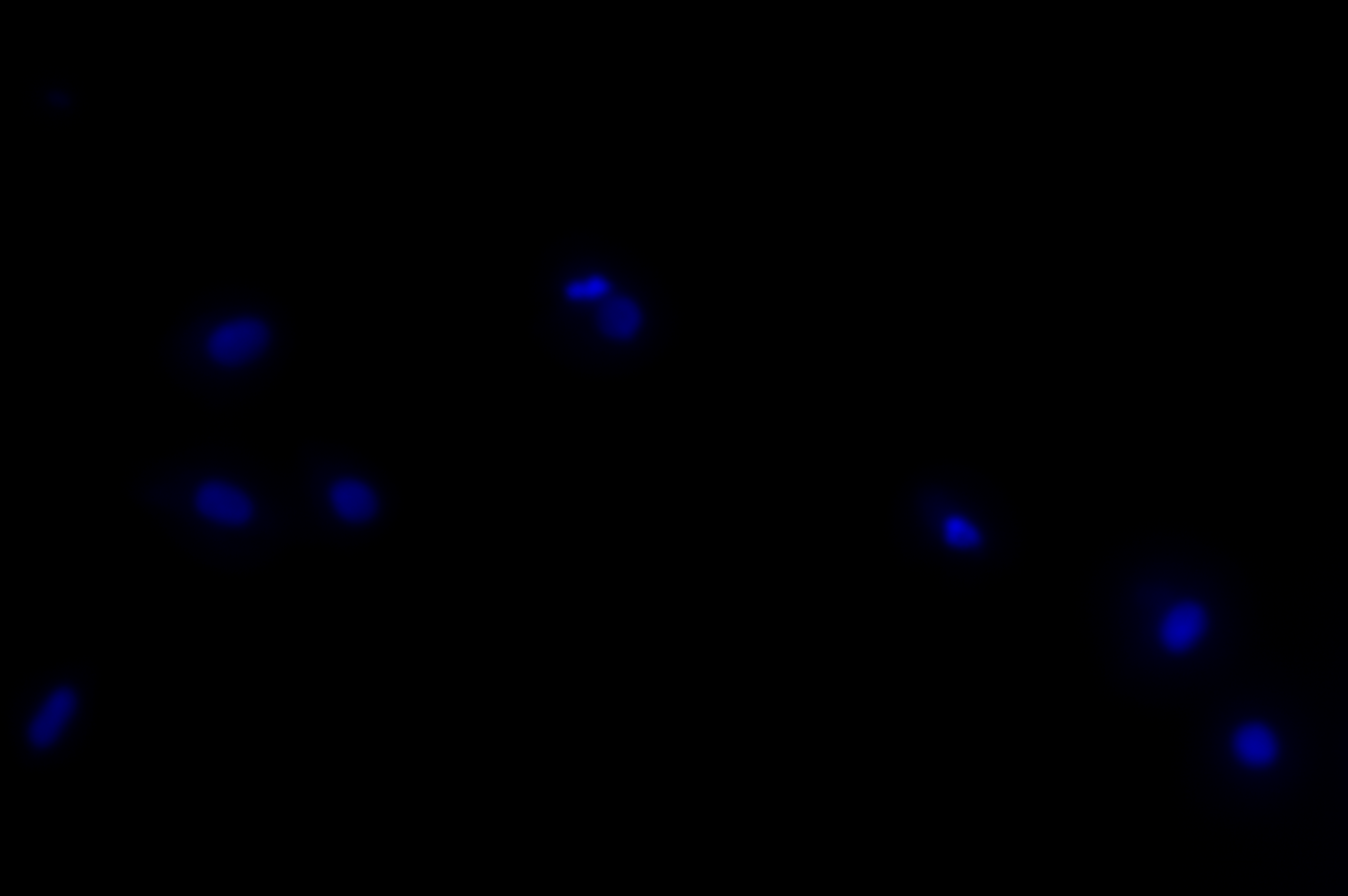

Supplement: Supplementary file 1 [file cells-15-01070-s001.zip › Supplementary File/Orginal image/Figure 14E_DAPI_MITF-OE.png]

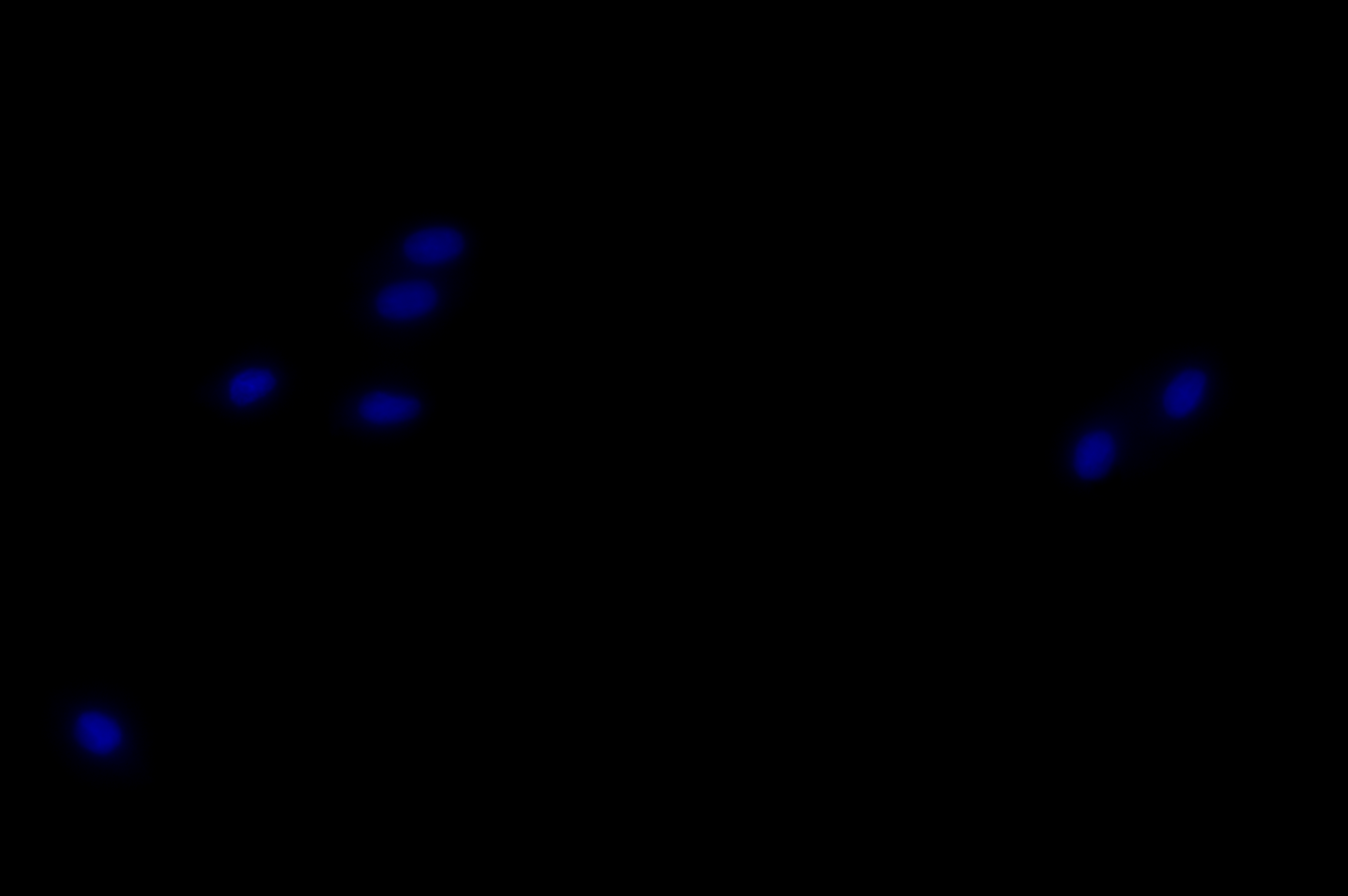

Supplement: Supplementary file 1 [file cells-15-01070-s001.zip › Supplementary File/Orginal image/Figure 14E_DAPI_Selinexor+MITF-OE.png]

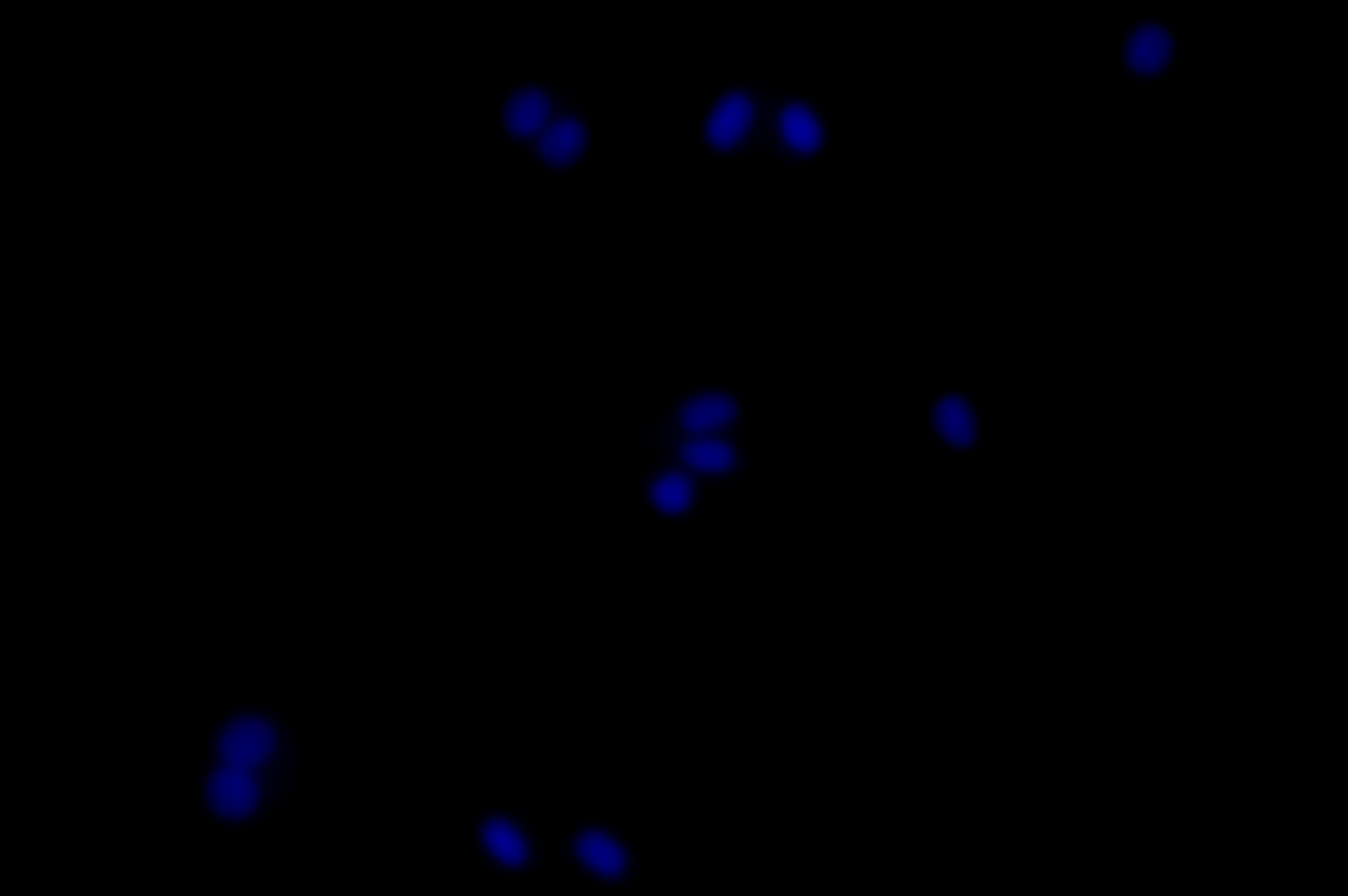

Supplement: Supplementary file 1 [file cells-15-01070-s001.zip › Supplementary File/Orginal image/Figure 14E_DAPI_Selinexor.png]

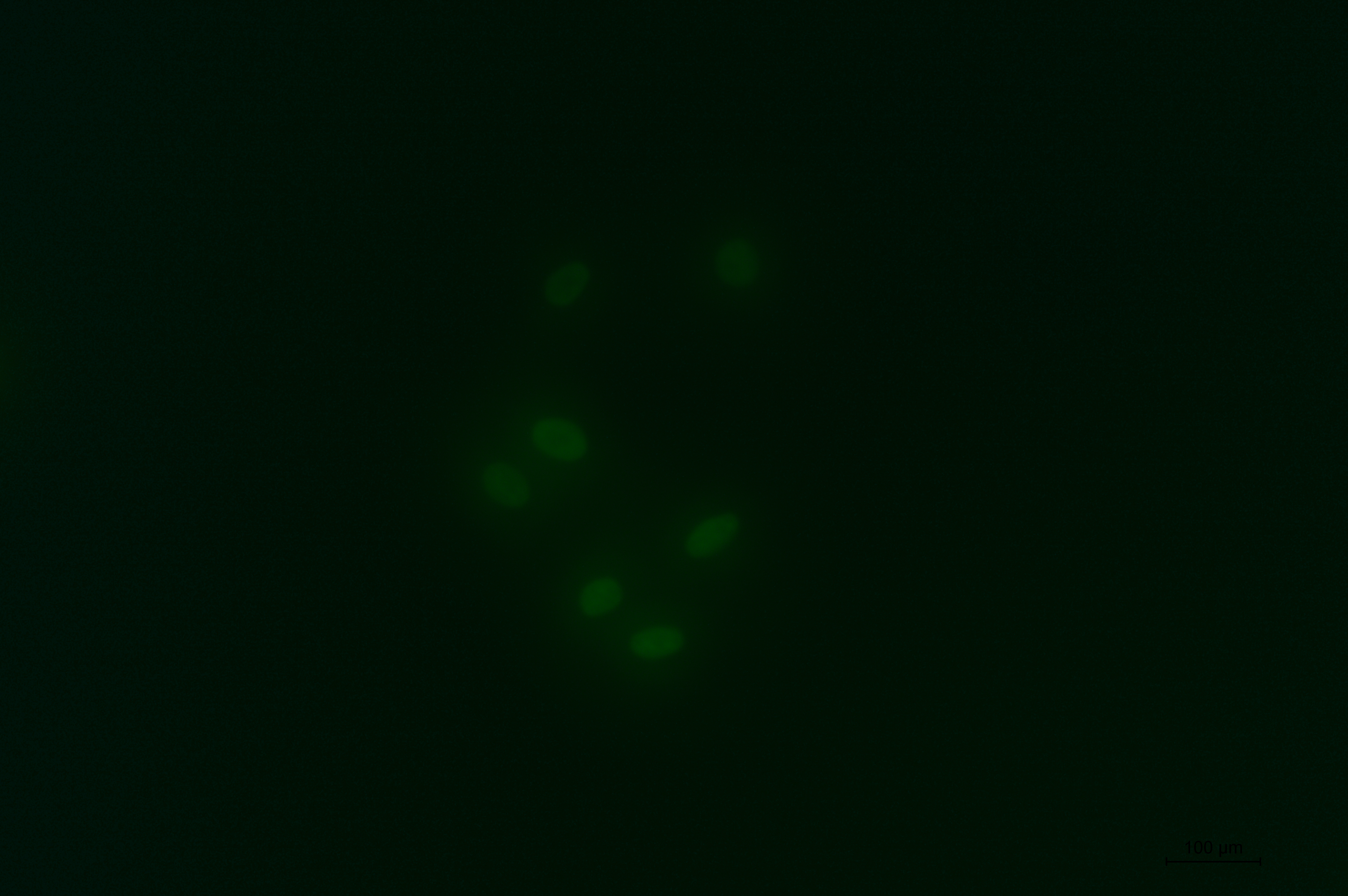

Supplement: Supplementary file 1 [file cells-15-01070-s001.zip › Supplementary File/Orginal image/Figure 14E_MITF_Con.tif]

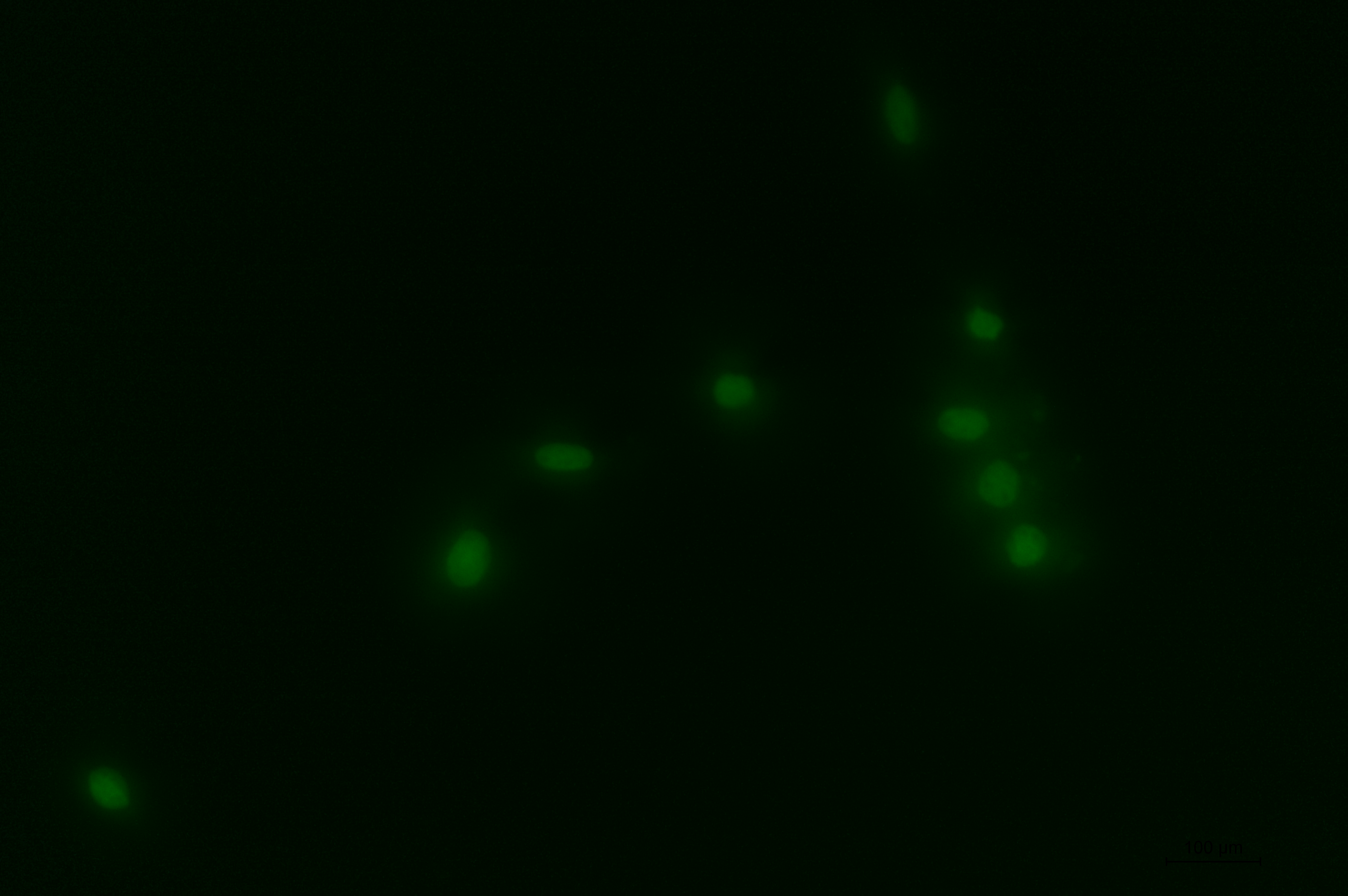

Supplement: Supplementary file 1 [file cells-15-01070-s001.zip › Supplementary File/Orginal image/Figure 14E_MITF_Methotrexate+MITF-OE.png]

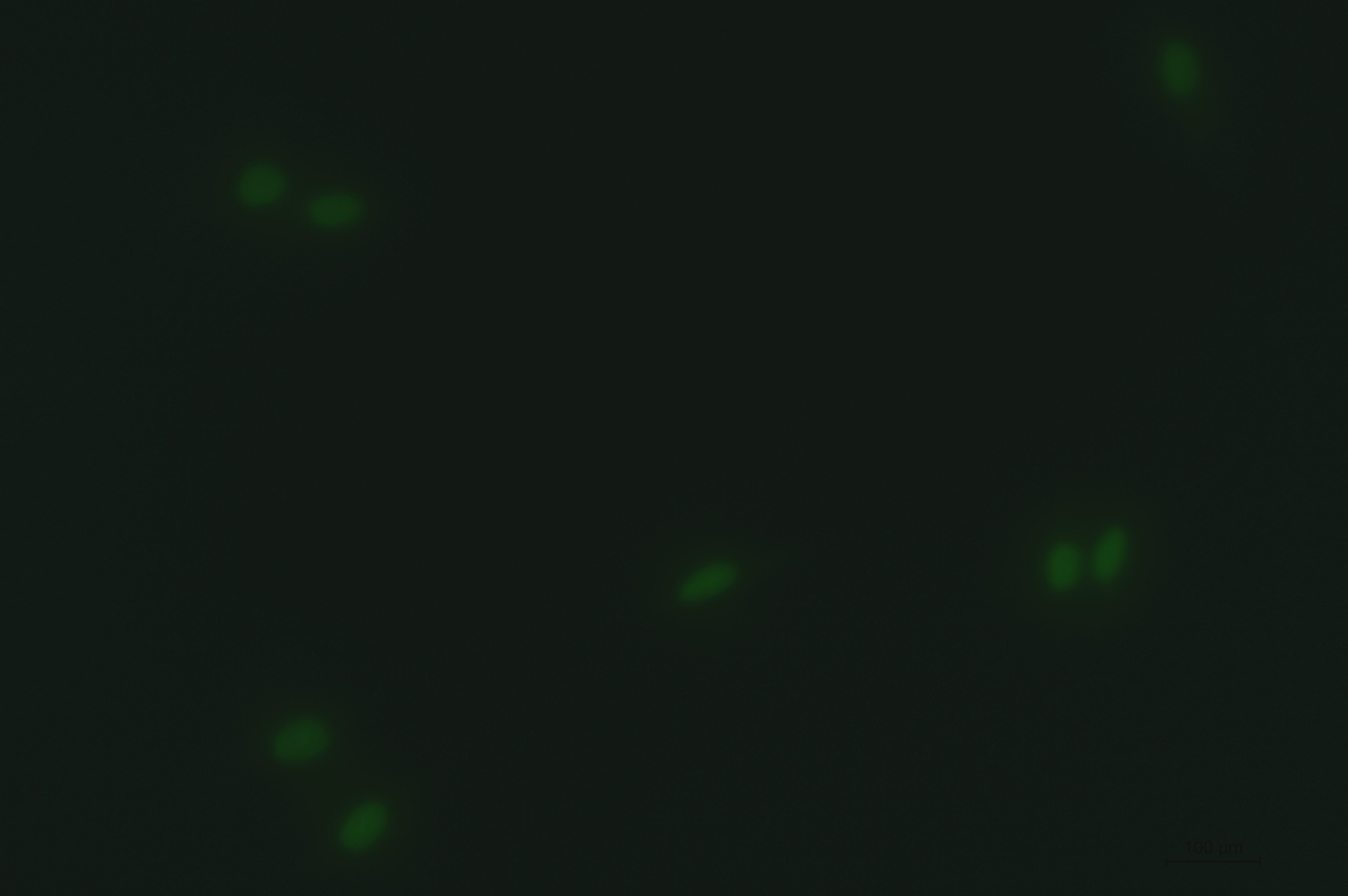

Supplement: Supplementary file 1 [file cells-15-01070-s001.zip › Supplementary File/Orginal image/Figure 14E_MITF_Methotrexate.png]

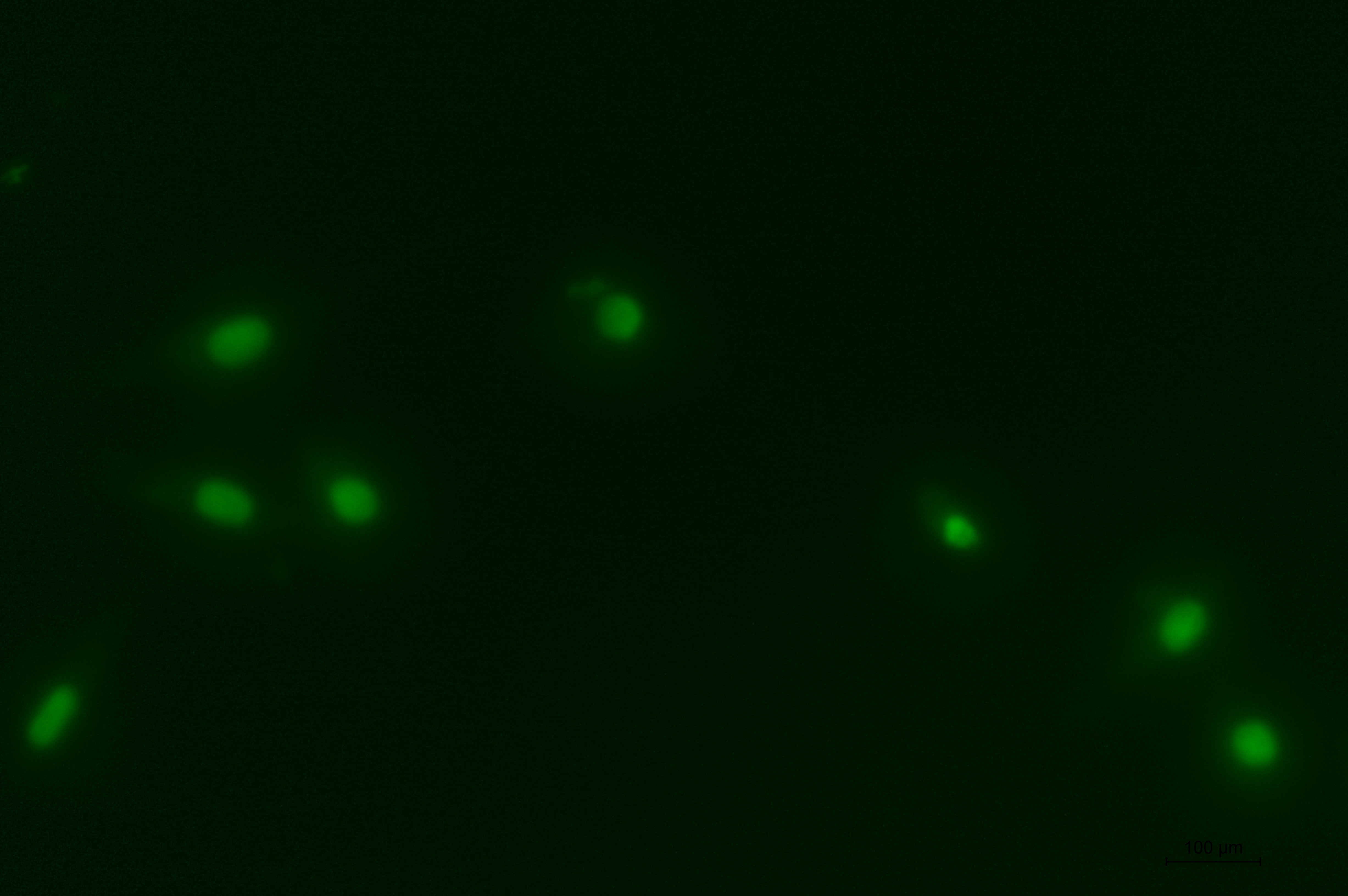

Supplement: Supplementary file 1 [file cells-15-01070-s001.zip › Supplementary File/Orginal image/Figure 14E_MITF_MITF-OE.png]

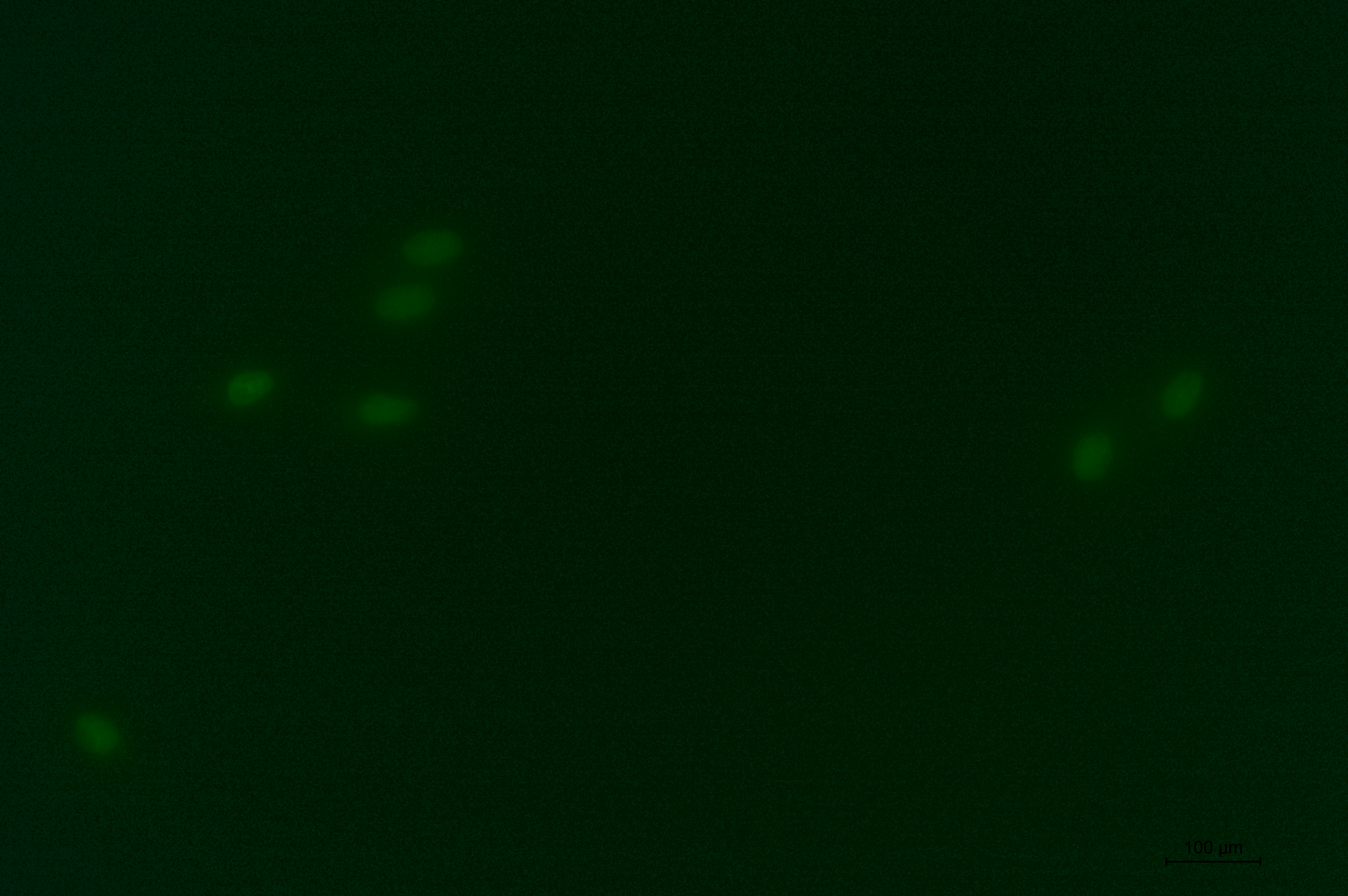

Supplement: Supplementary file 1 [file cells-15-01070-s001.zip › Supplementary File/Orginal image/Figure 14E_MITF_Selinexor+MITF-OE.png]

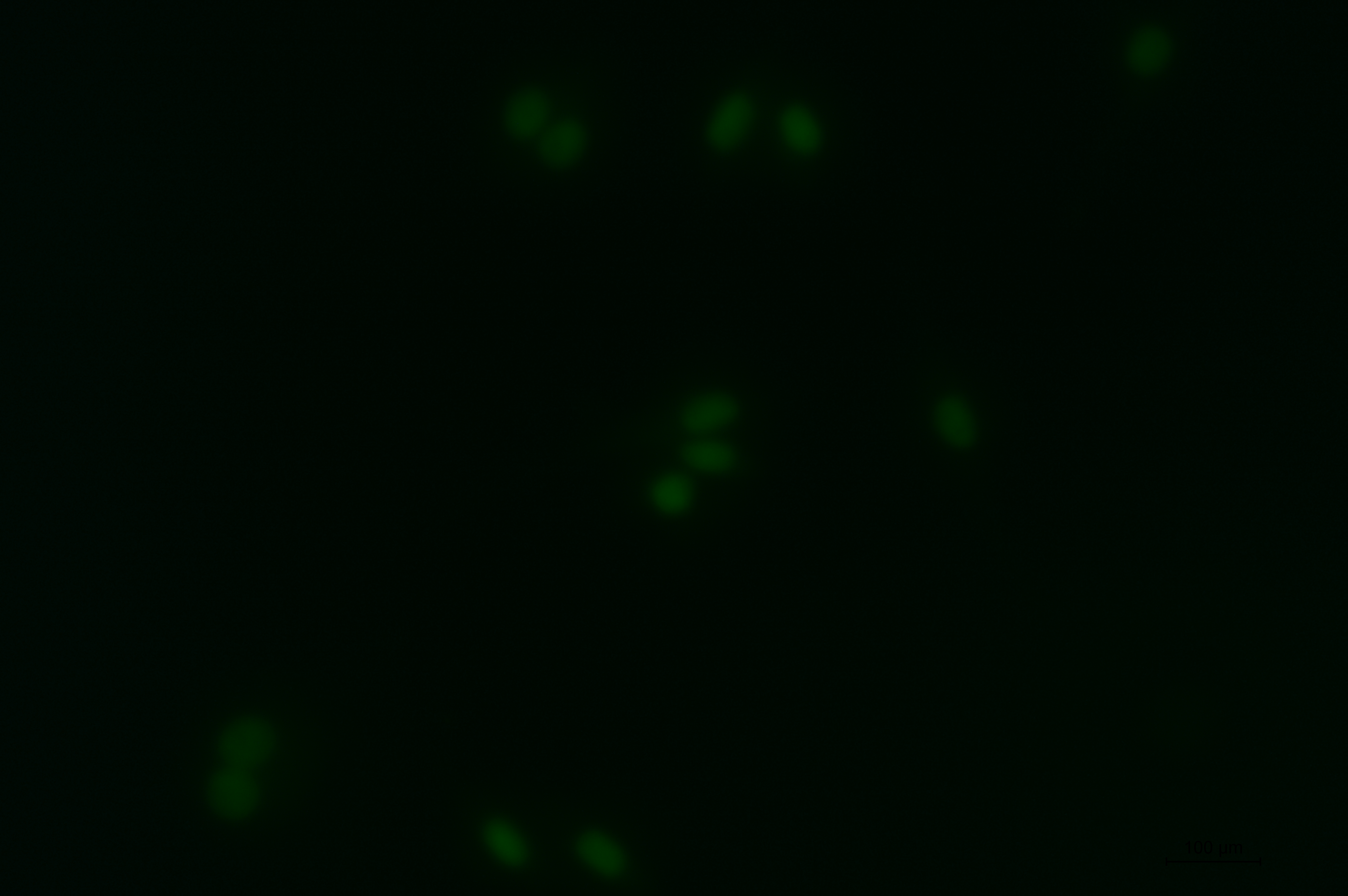

Supplement: Supplementary file 1 [file cells-15-01070-s001.zip › Supplementary File/Orginal image/Figure 14E_MITF_Selinexor.png]

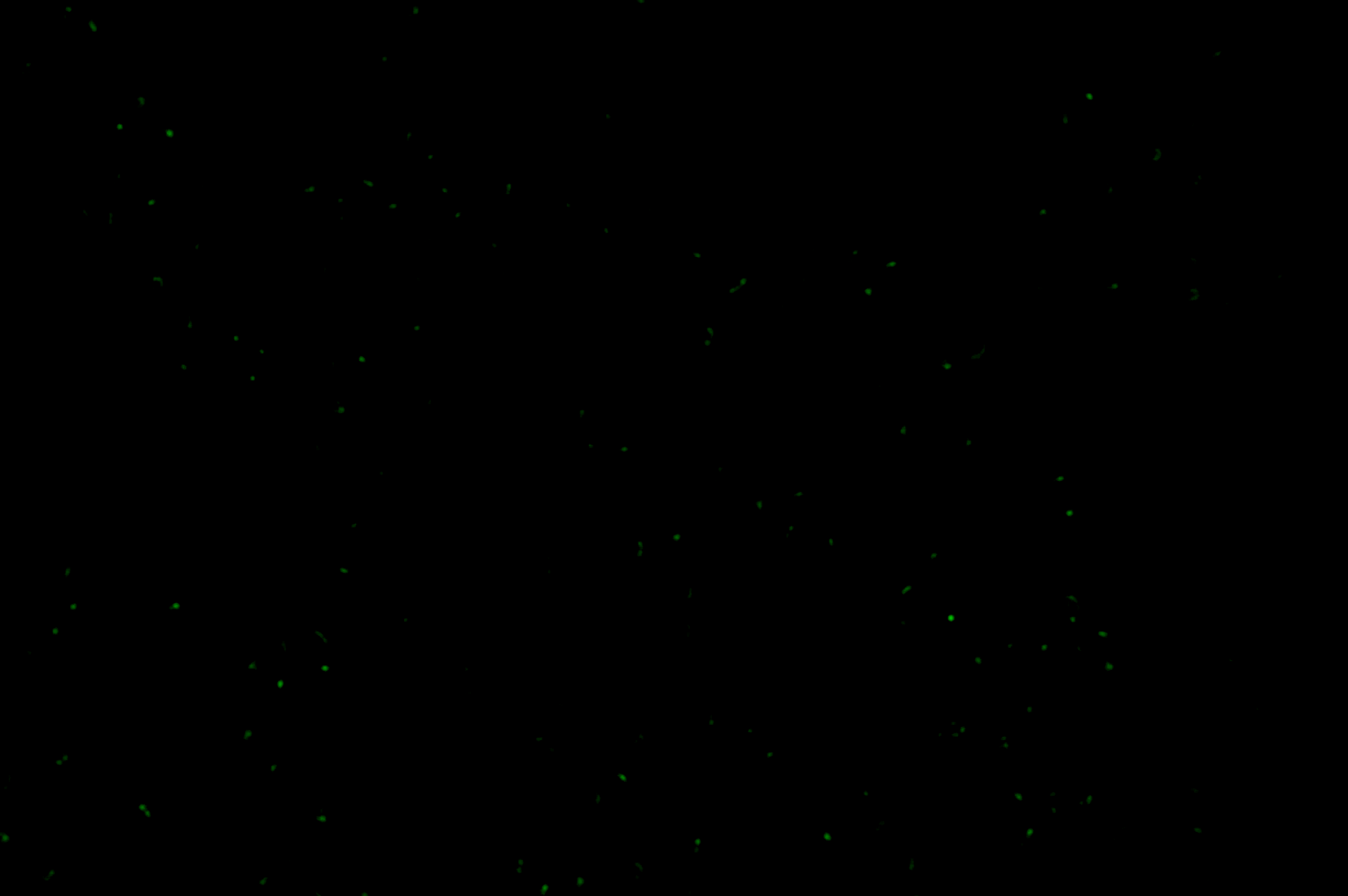

Supplement: Supplementary file 1 [file cells-15-01070-s001.zip › Supplementary File/Orginal image/Figure 8A_Control.png]

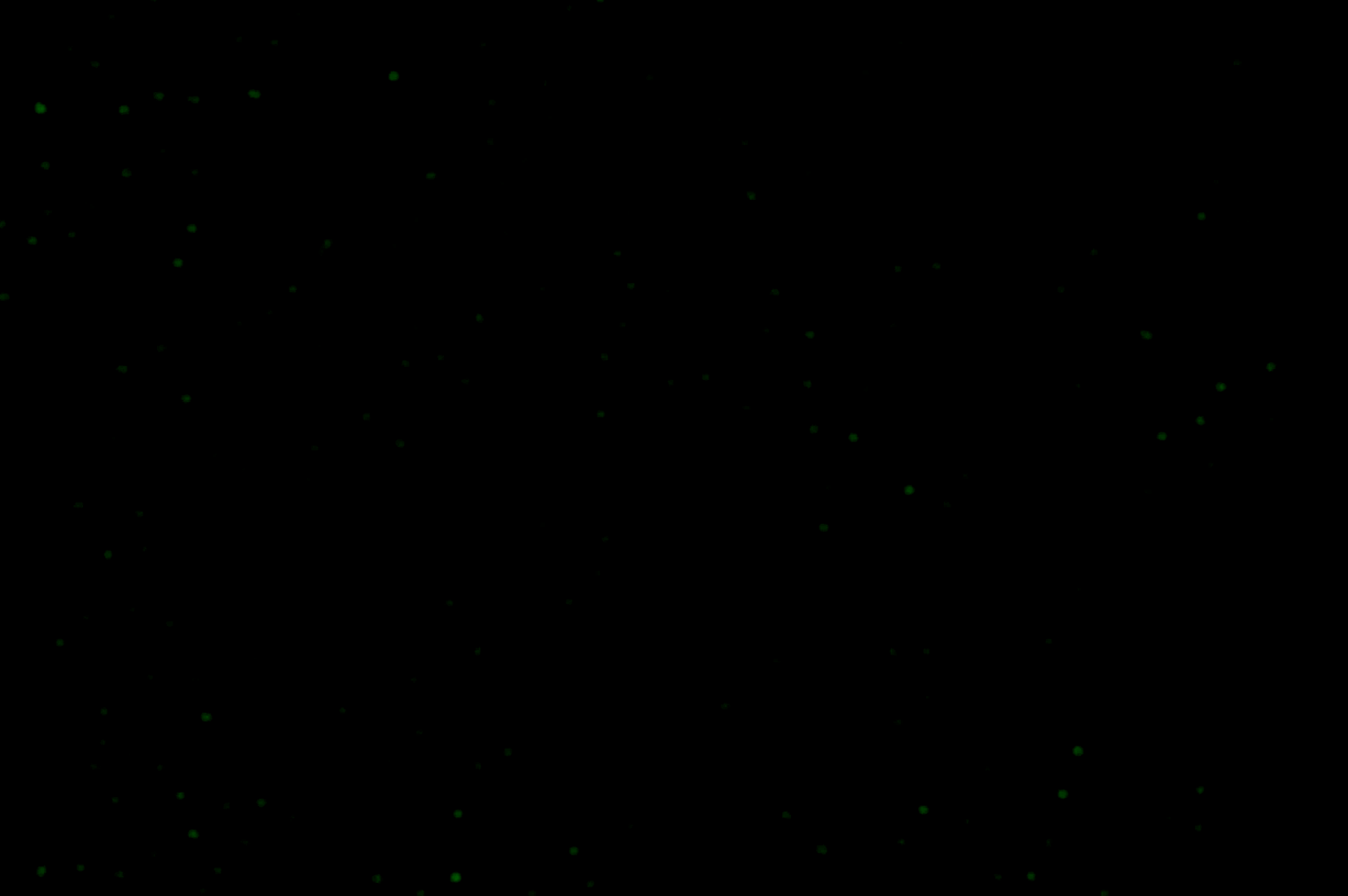

Supplement: Supplementary file 1 [file cells-15-01070-s001.zip › Supplementary File/Orginal image/Figure 8A_Methotrexate_25.png]

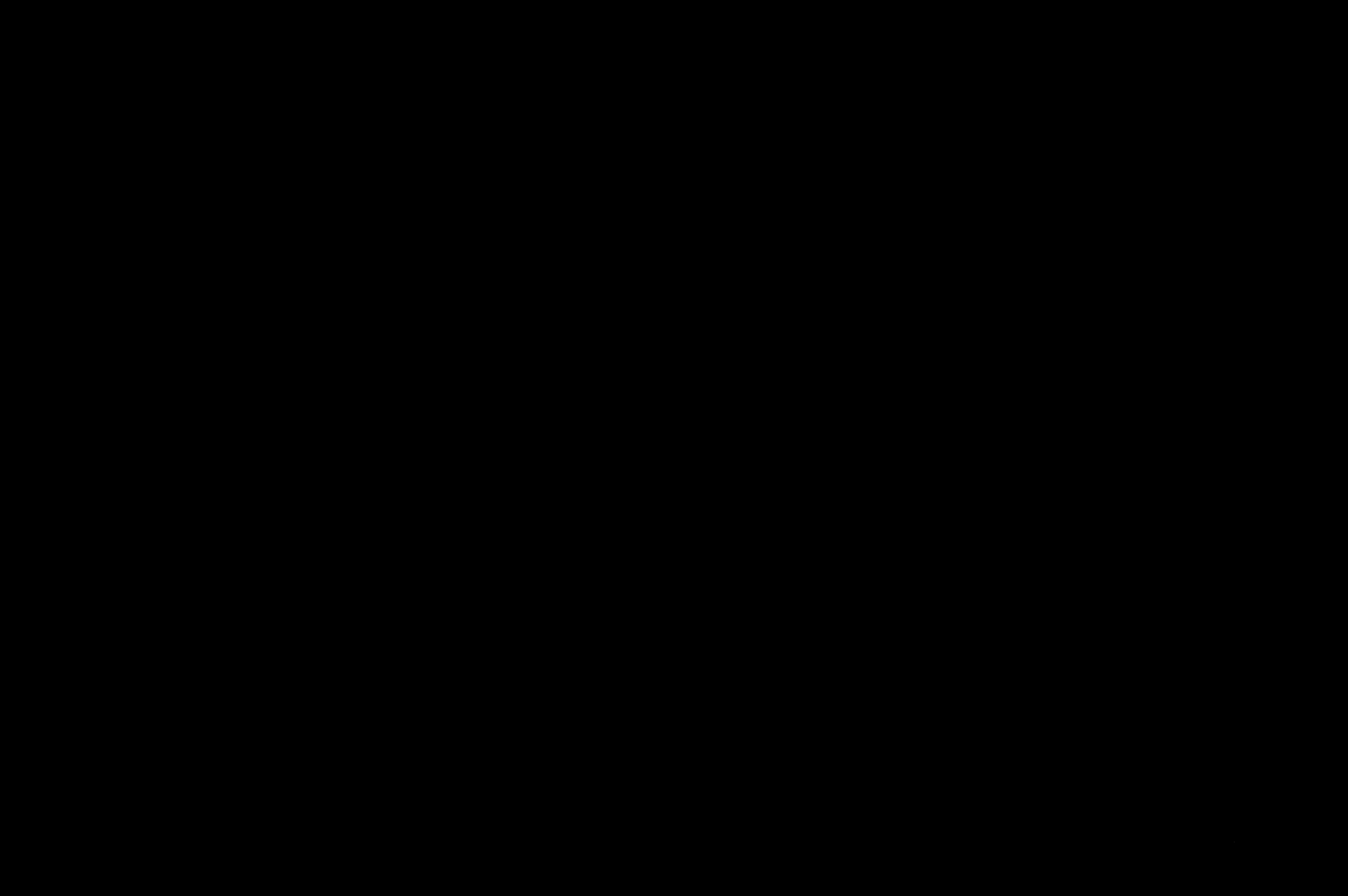

Supplement: Supplementary file 1 [file cells-15-01070-s001.zip › Supplementary File/Orginal image/Figure 8A_Methotrexate_75.jpg]

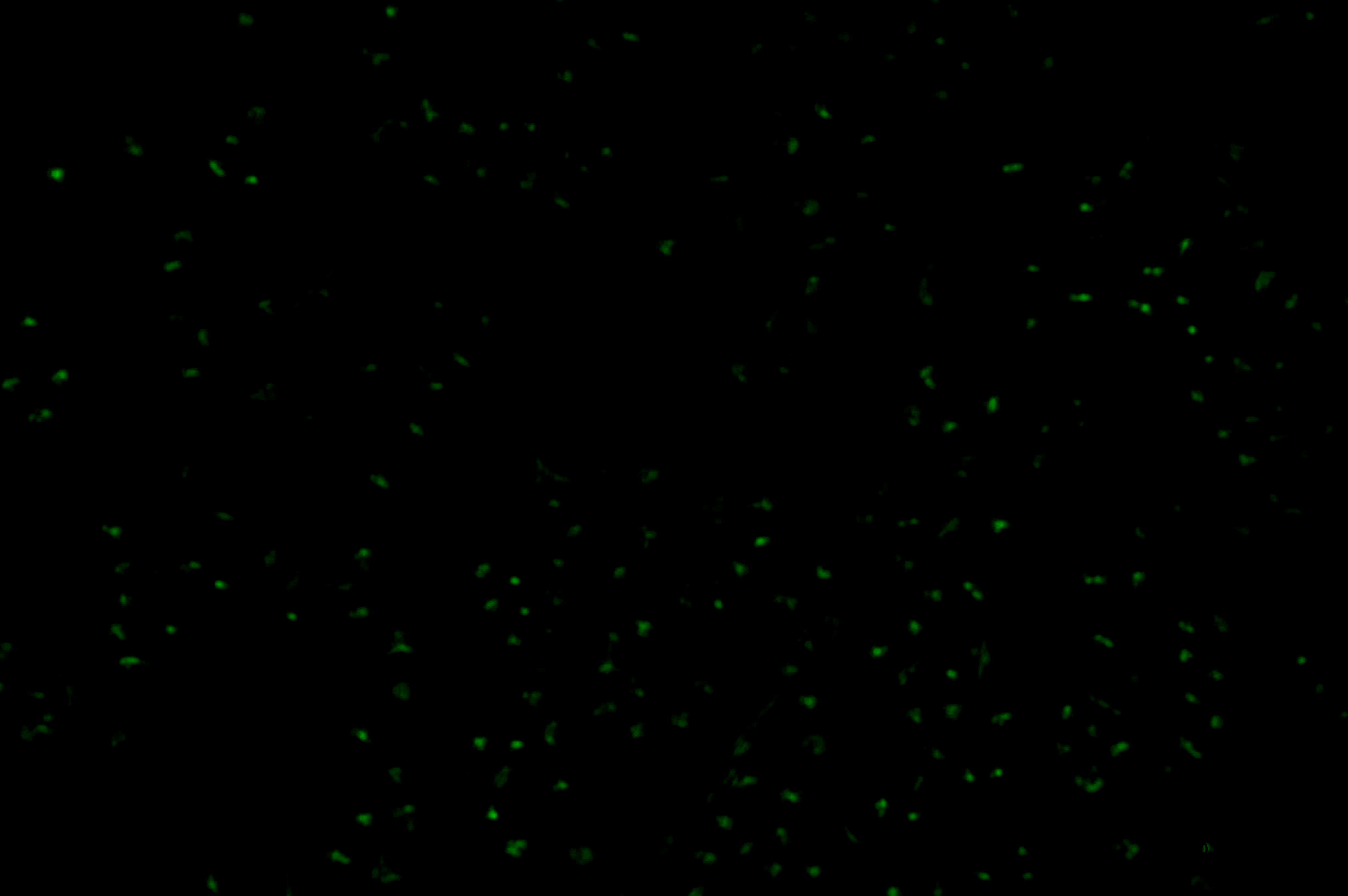

Supplement: Supplementary file 1 [file cells-15-01070-s001.zip › Supplementary File/Orginal image/Figure 8A_Positive control + Methotrexate_25.png]

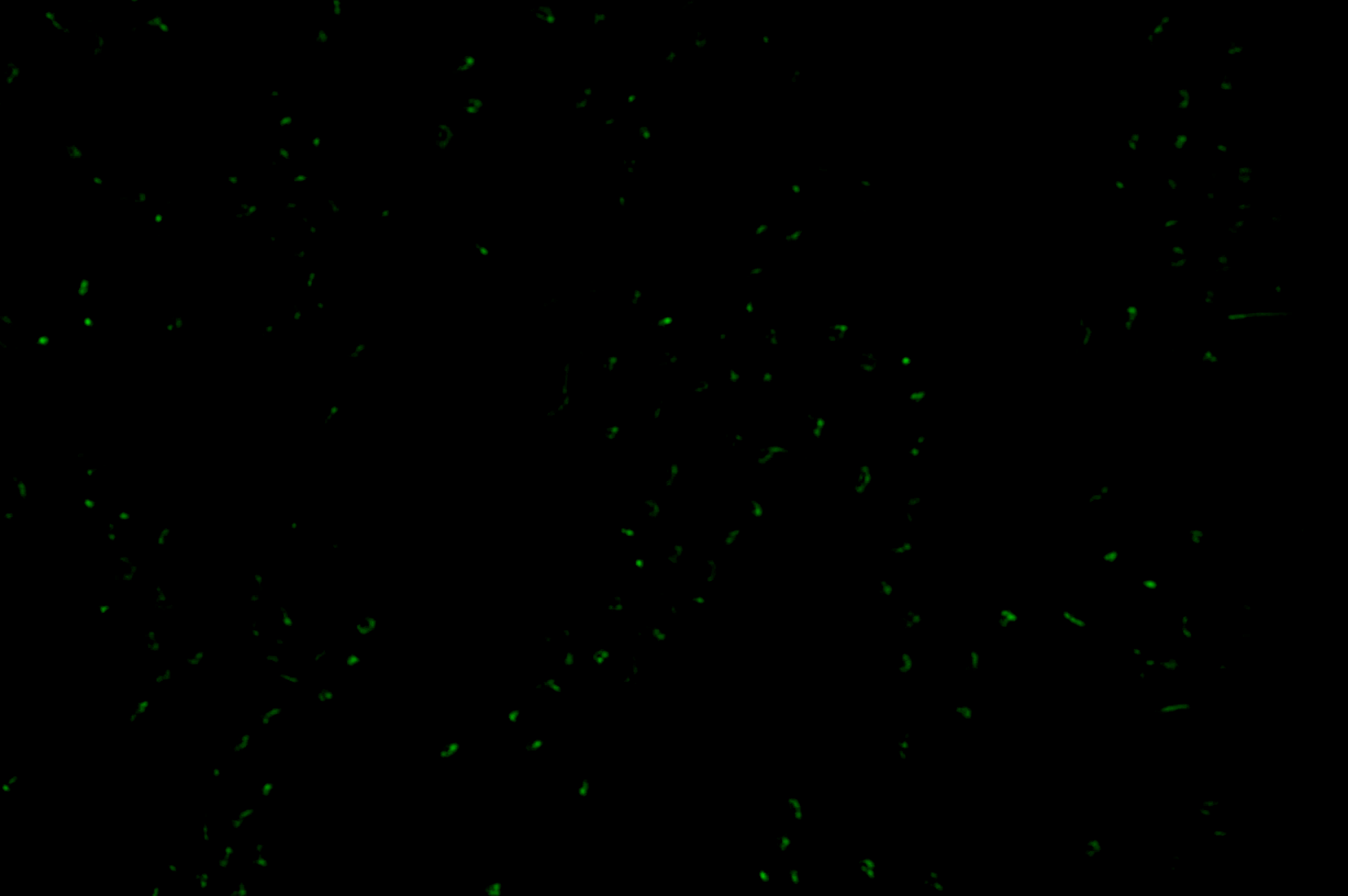

Supplement: Supplementary file 1 [file cells-15-01070-s001.zip › Supplementary File/Orginal image/Figure 8A_Positive control + Methotrexate_75.png]

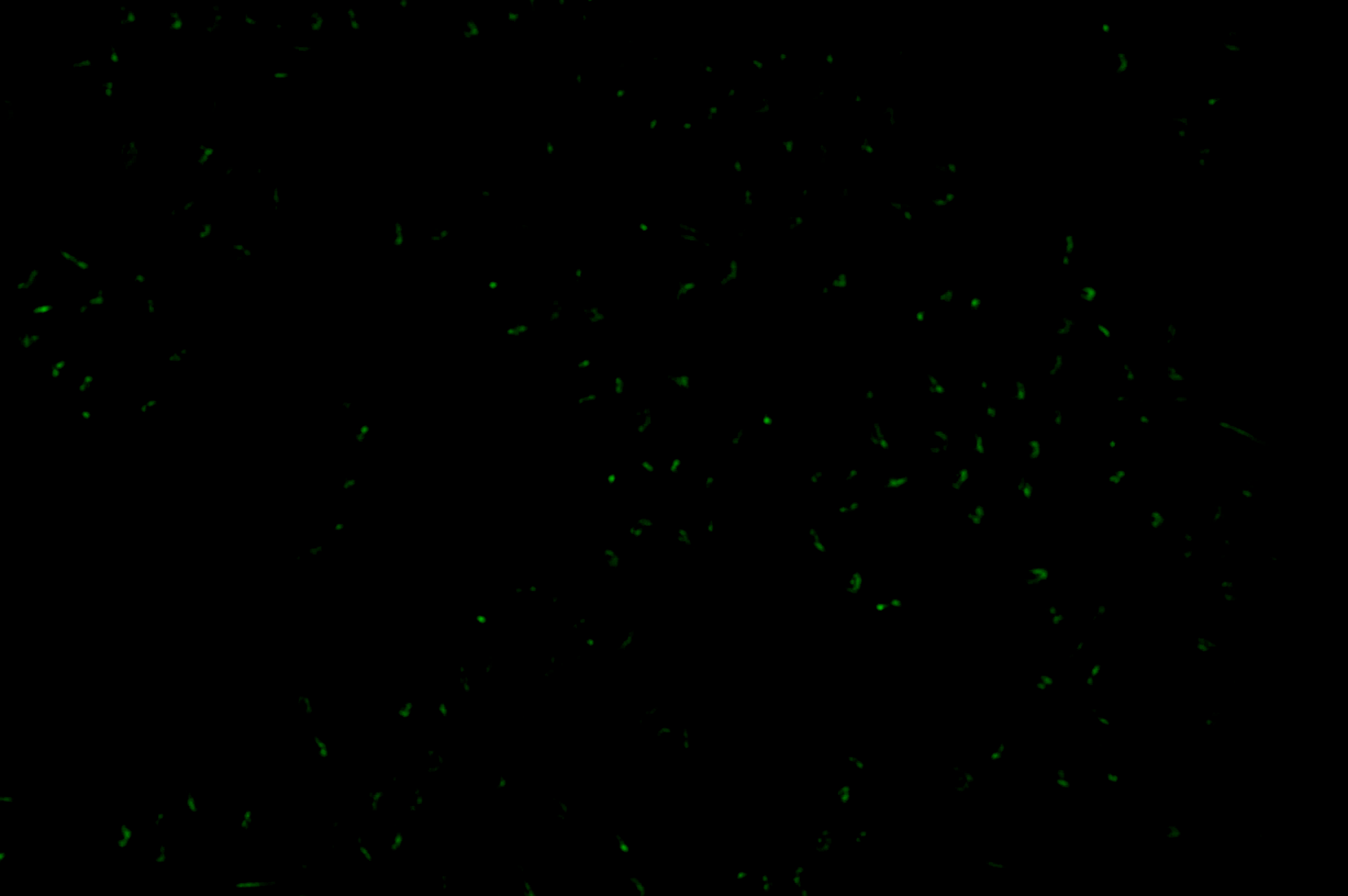

Supplement: Supplementary file 1 [file cells-15-01070-s001.zip › Supplementary File/Orginal image/Figure 8A_Positive control +Selinexor_100.png]

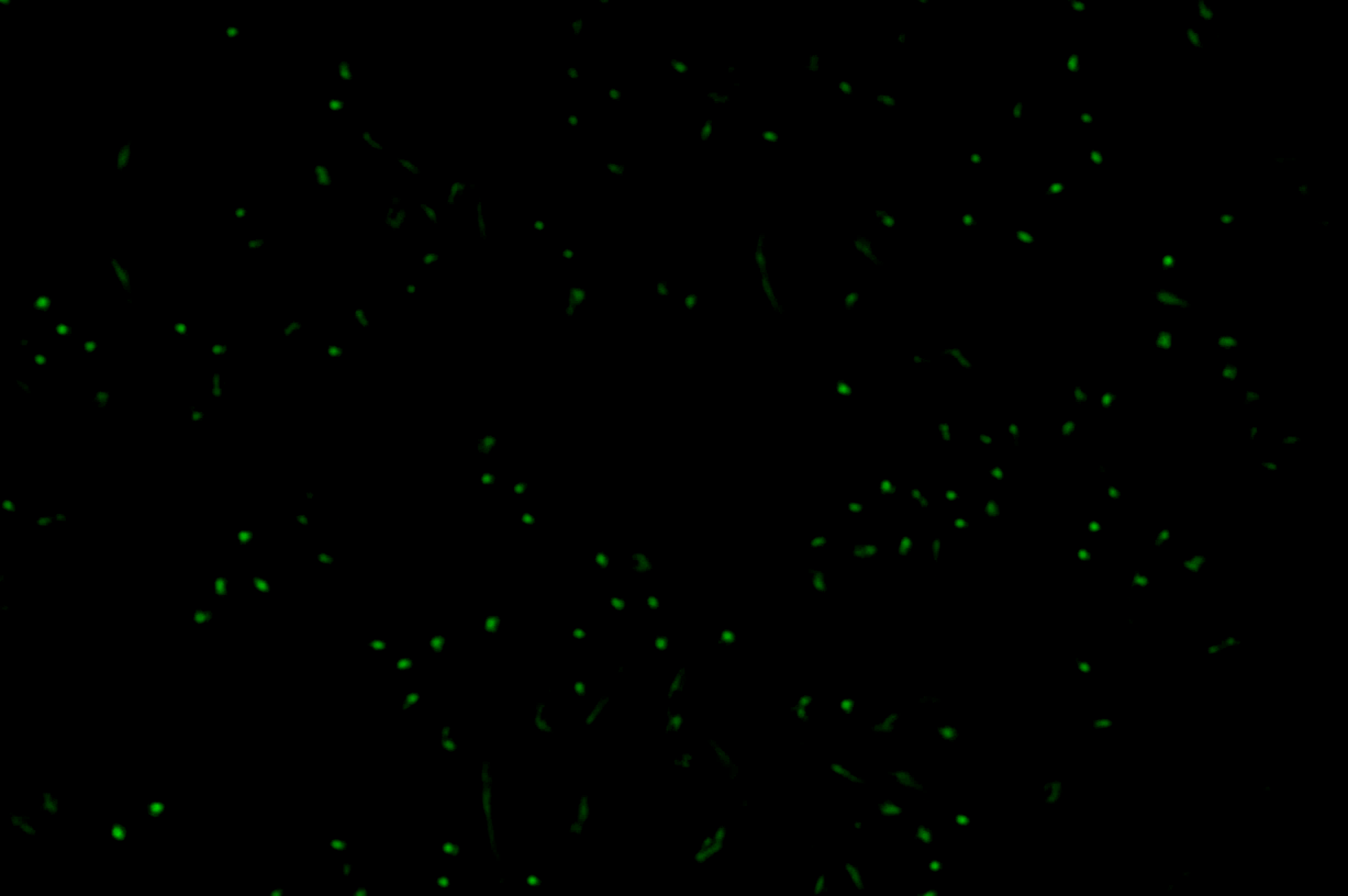

Supplement: Supplementary file 1 [file cells-15-01070-s001.zip › Supplementary File/Orginal image/Figure 8A_Positive control +Selinexor_25.png]

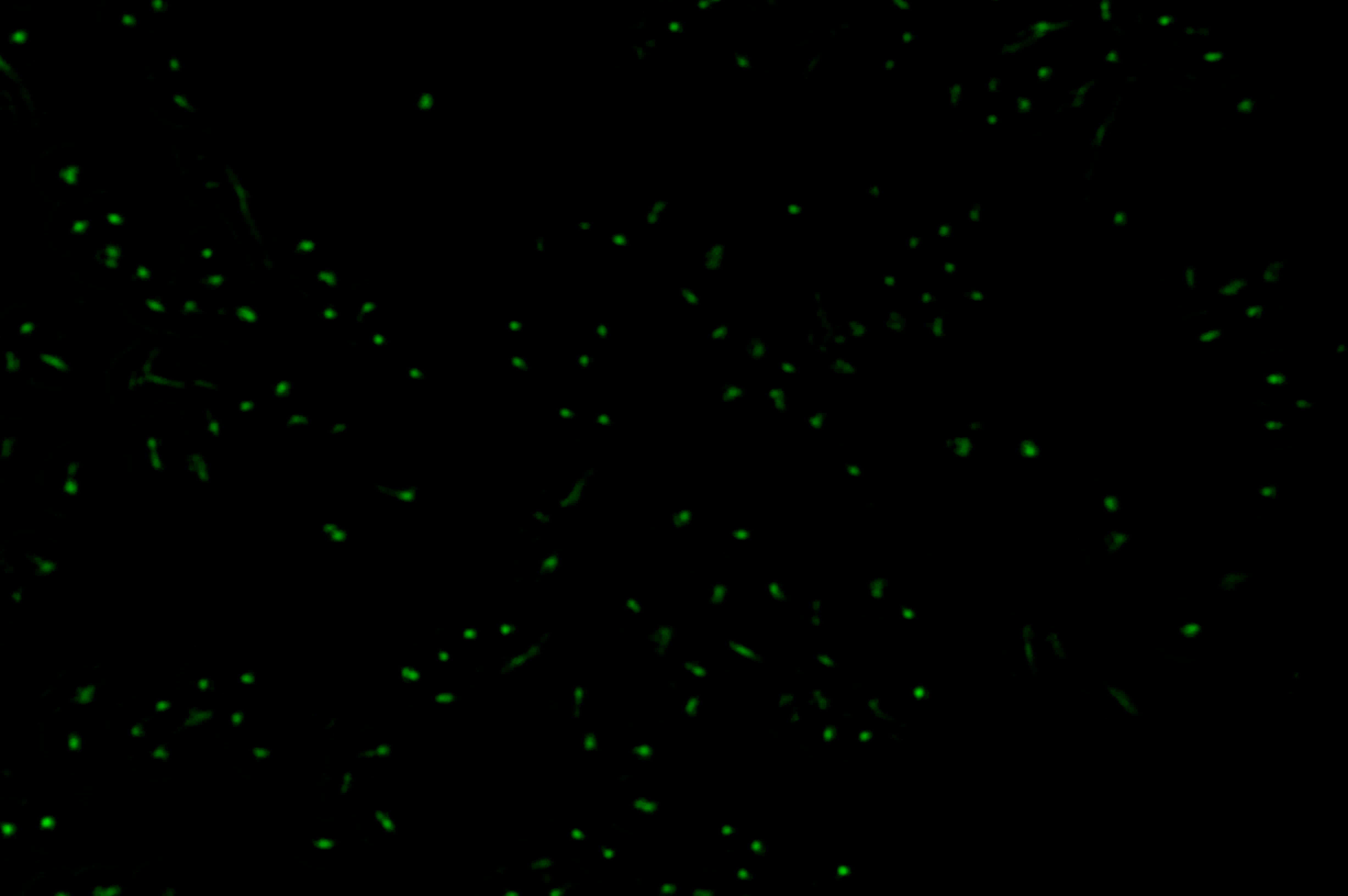

Supplement: Supplementary file 1 [file cells-15-01070-s001.zip › Supplementary File/Orginal image/Figure 8A_Positive control.png]

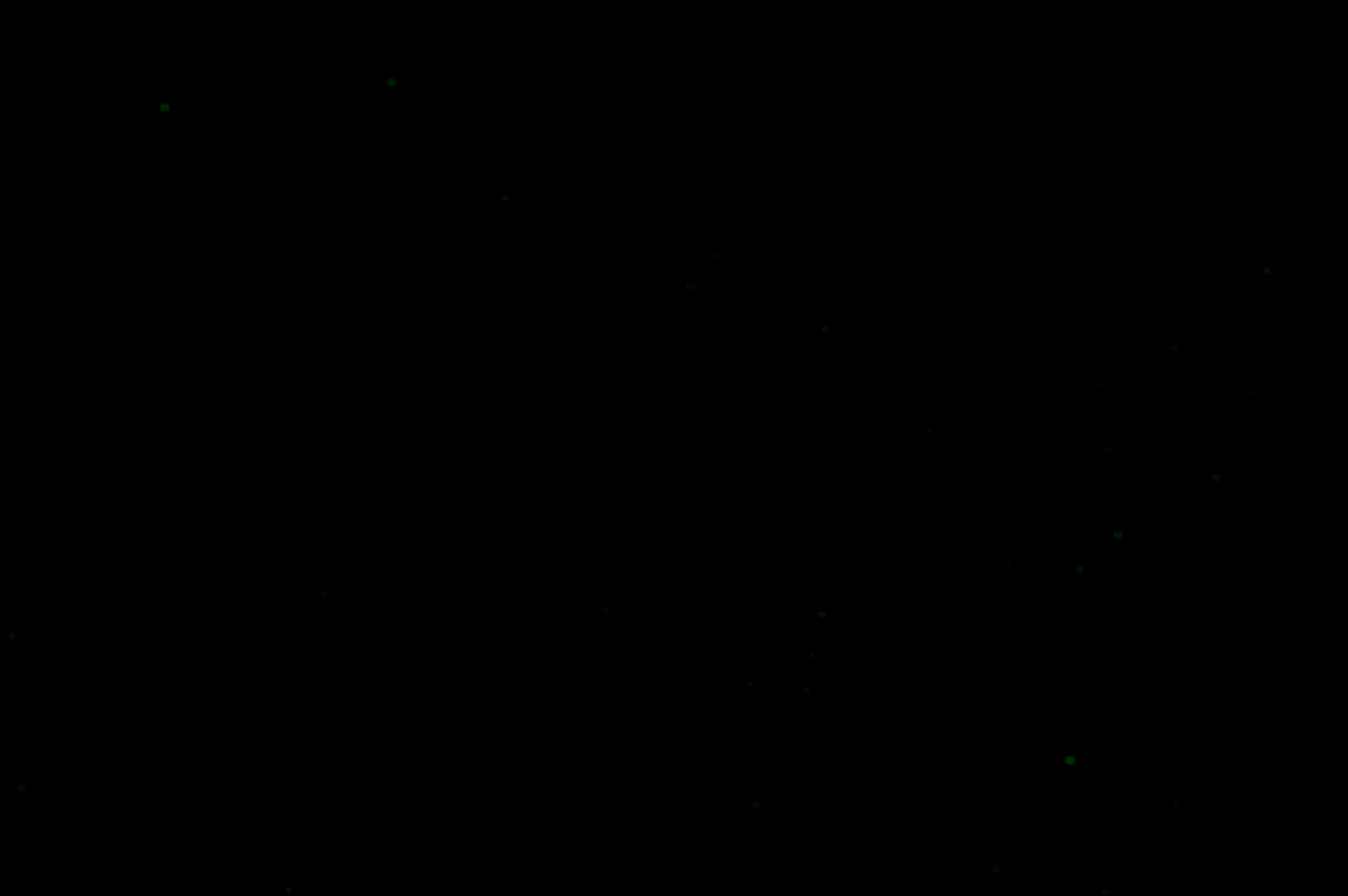

Supplement: Supplementary file 1 [file cells-15-01070-s001.zip › Supplementary File/Orginal image/Figure 8A_Selinexor_100.png]

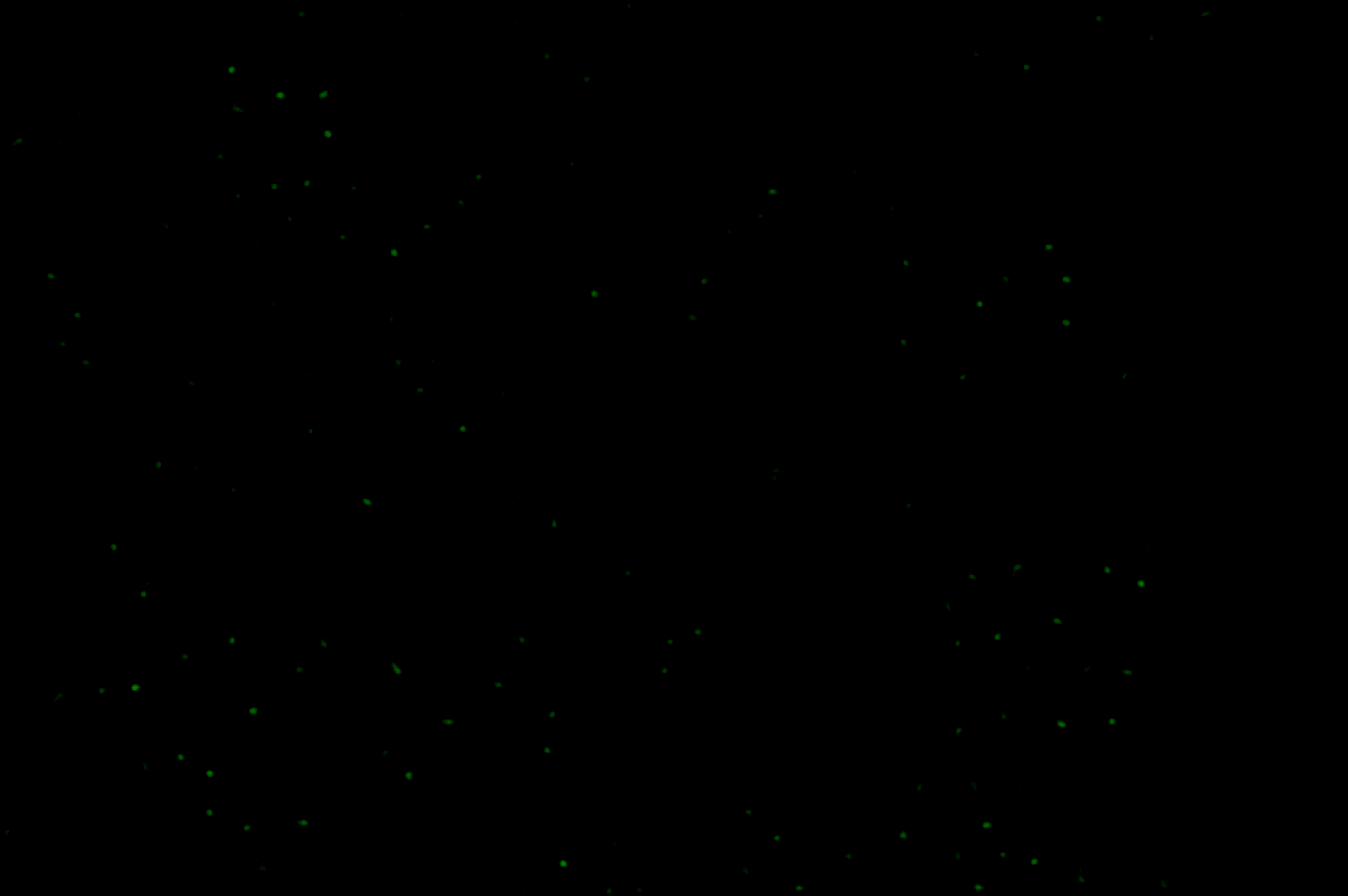

Supplement: Supplementary file 1 [file cells-15-01070-s001.zip › Supplementary File/Orginal image/Figure 8A_Selinexor_25.png]

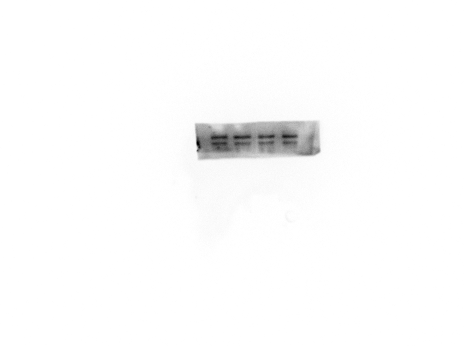

Supplement: Supplementary file 1 [file cells-15-01070-s001.zip › Supplementary File/Orginal image/Figure. 11A_MITF.png]

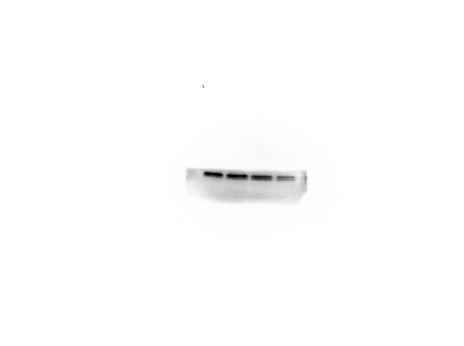

Supplement: Supplementary file 1 [file cells-15-01070-s001.zip › Supplementary File/Orginal image/Figure. 11A_TRP1.png]

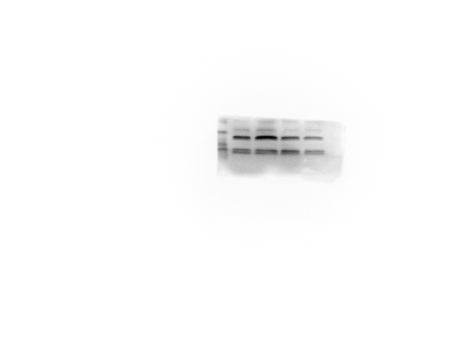

Supplement: Supplementary file 1 [file cells-15-01070-s001.zip › Supplementary File/Orginal image/Figure. 11A_TRP2.png]

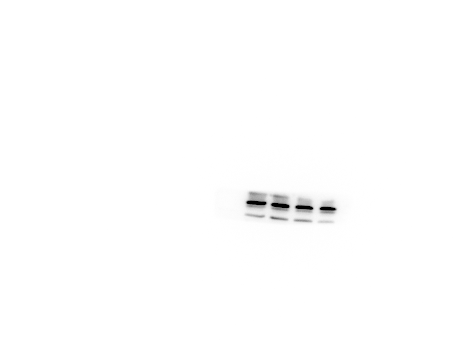

Supplement: Supplementary file 1 [file cells-15-01070-s001.zip › Supplementary File/Orginal image/Figure. 11A_TYR.png]

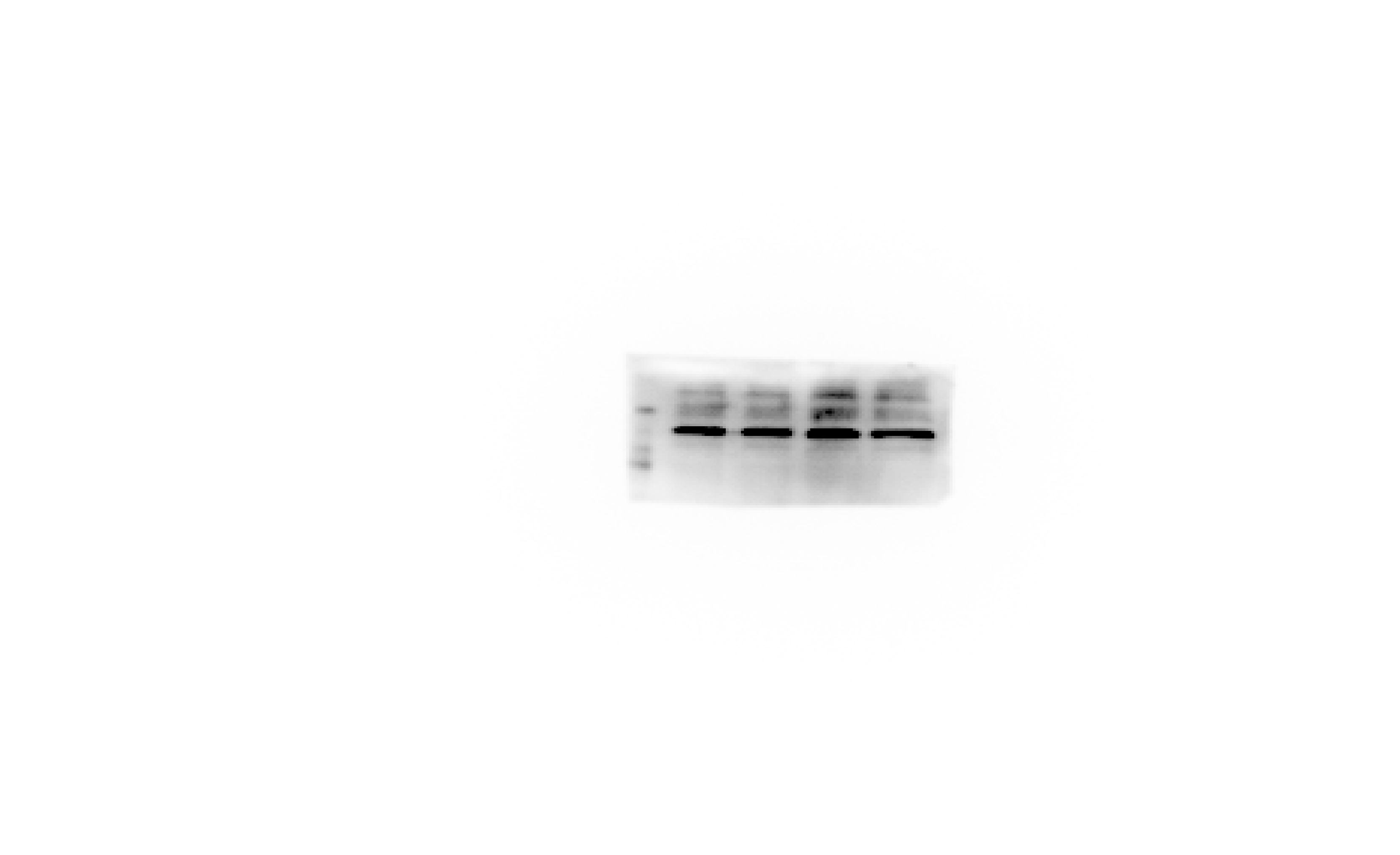

Supplement: Supplementary file 1 [file cells-15-01070-s001.zip › Supplementary File/Orginal image/Figure. 11A_β-actin.png]

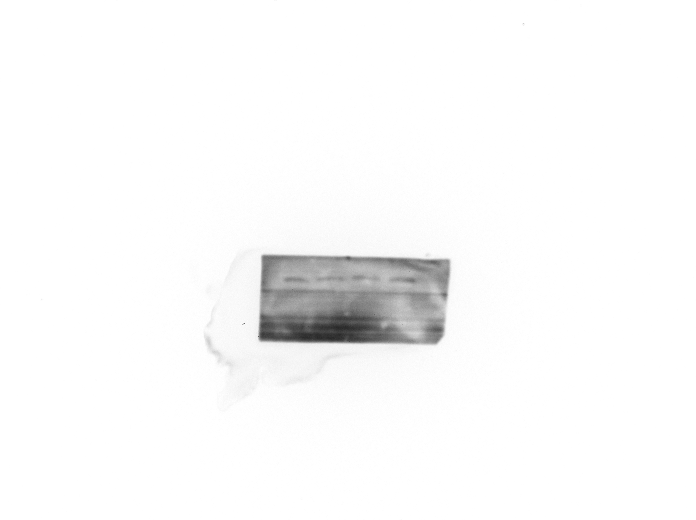

Supplement: Supplementary file 1 [file cells-15-01070-s001.zip › Supplementary File/Orginal image/Figure. 11C_MITF.png]

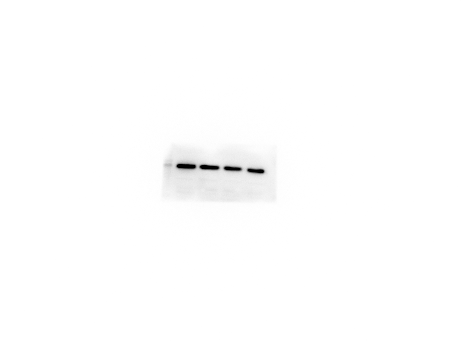

Supplement: Supplementary file 1 [file cells-15-01070-s001.zip › Supplementary File/Orginal image/Figure. 11C_TRP1.png]

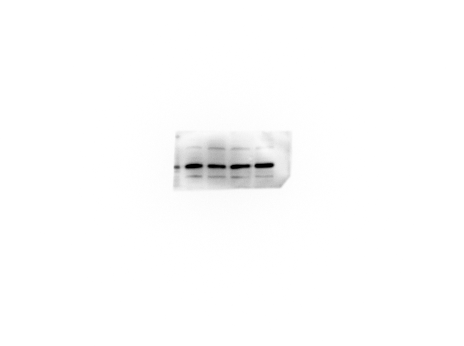

Supplement: Supplementary file 1 [file cells-15-01070-s001.zip › Supplementary File/Orginal image/Figure. 11C_TRP2.png]

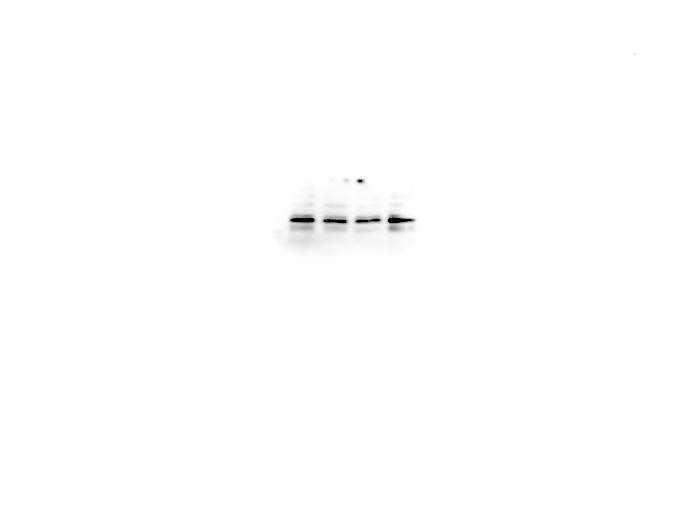

Supplement: Supplementary file 1 [file cells-15-01070-s001.zip › Supplementary File/Orginal image/Figure. 11C_TYR.png]

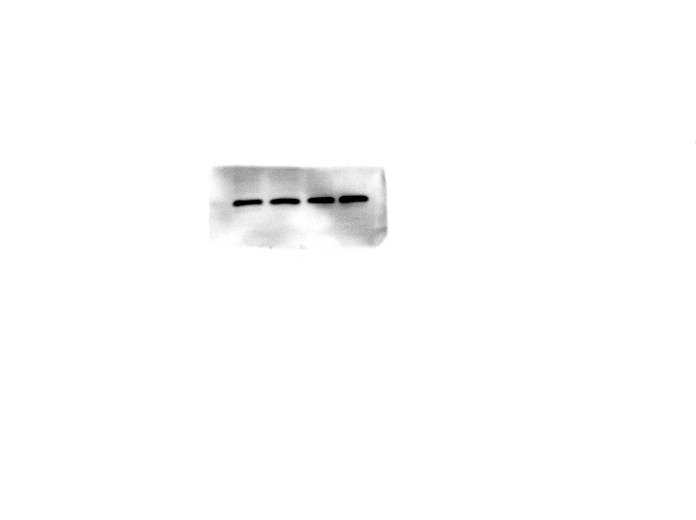

Supplement: Supplementary file 1 [file cells-15-01070-s001.zip › Supplementary File/Orginal image/Figure. 11C_β-actin.png]

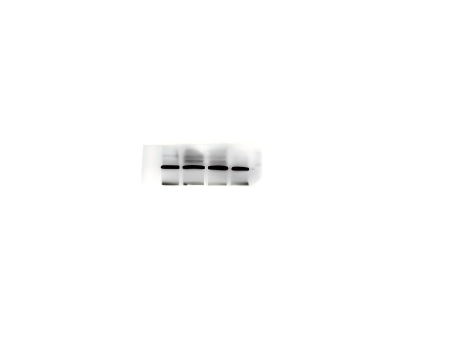

Supplement: Supplementary file 1 [file cells-15-01070-s001.zip › Supplementary File/Orginal image/Figure. 12A_CREB.png]

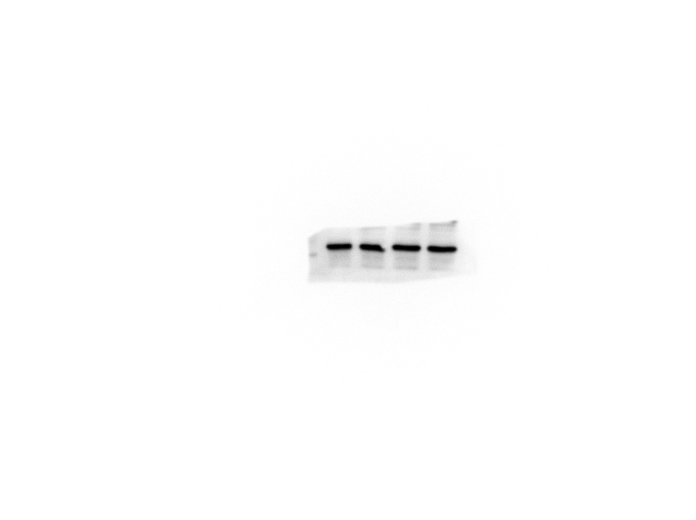

Supplement: Supplementary file 1 [file cells-15-01070-s001.zip › Supplementary File/Orginal image/Figure. 12A_GSK-3β.png]

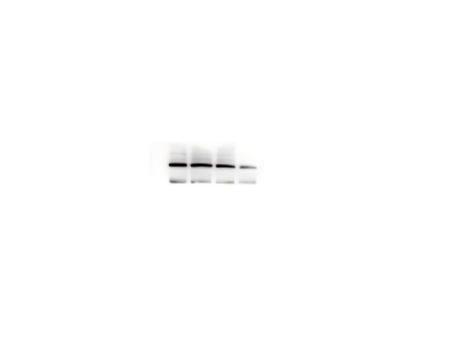

Supplement: Supplementary file 1 [file cells-15-01070-s001.zip › Supplementary File/Orginal image/Figure. 12A_p-CREB.png]

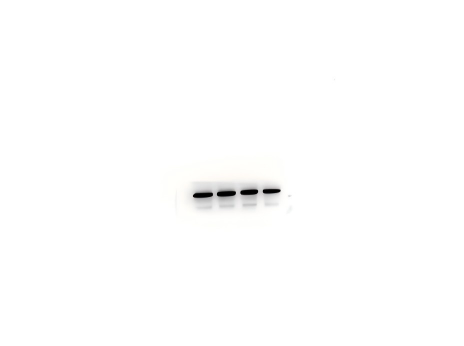

Supplement: Supplementary file 1 [file cells-15-01070-s001.zip › Supplementary File/Orginal image/Figure. 12A_p-GSK-3β.png]

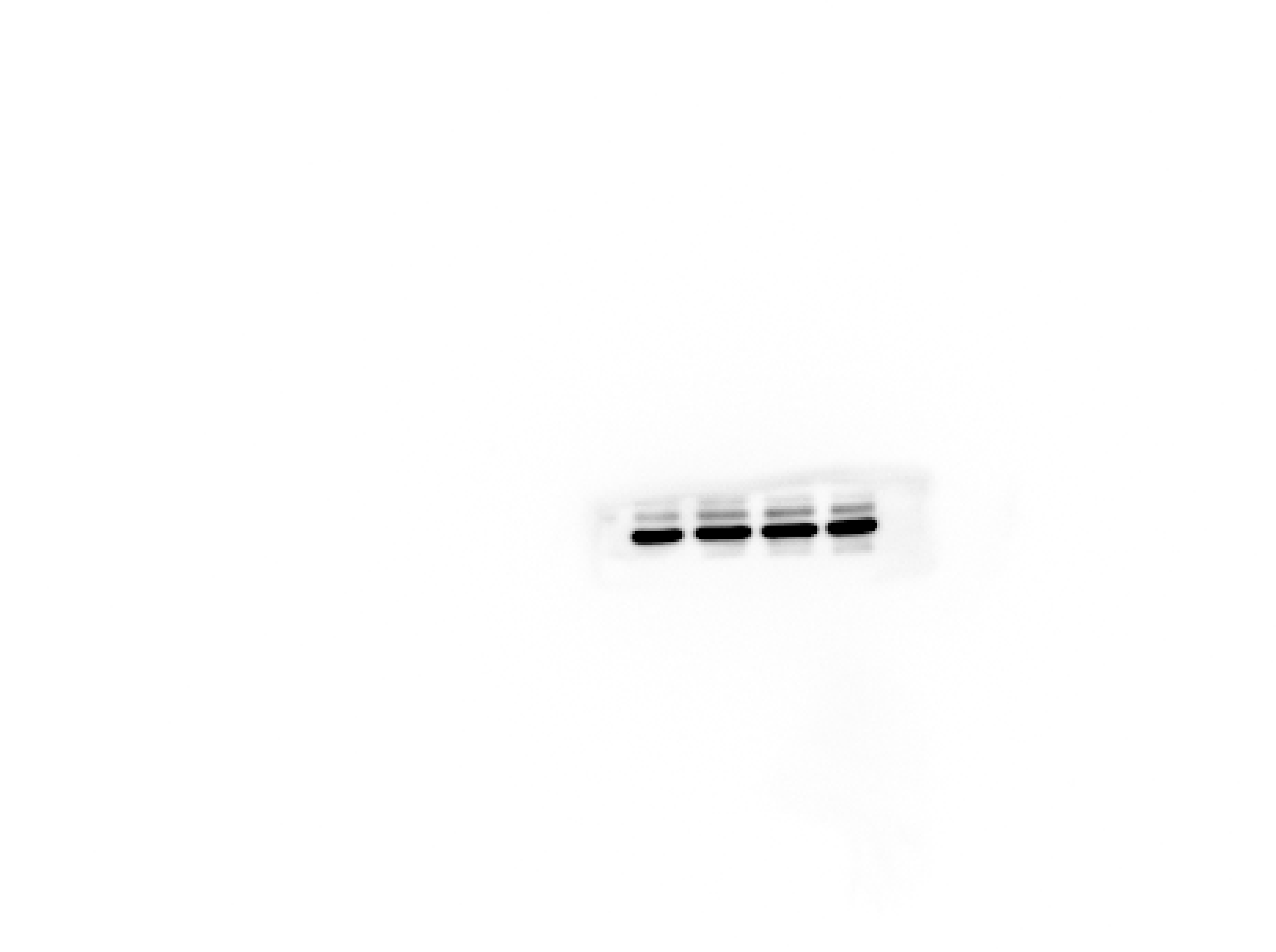

Supplement: Supplementary file 1 [file cells-15-01070-s001.zip › Supplementary File/Orginal image/Figure. 12A_β-actin.png]

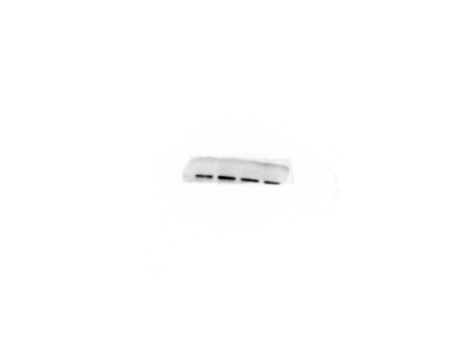

Supplement: Supplementary file 1 [file cells-15-01070-s001.zip › Supplementary File/Orginal image/Figure. 12A_β-catenin.png]

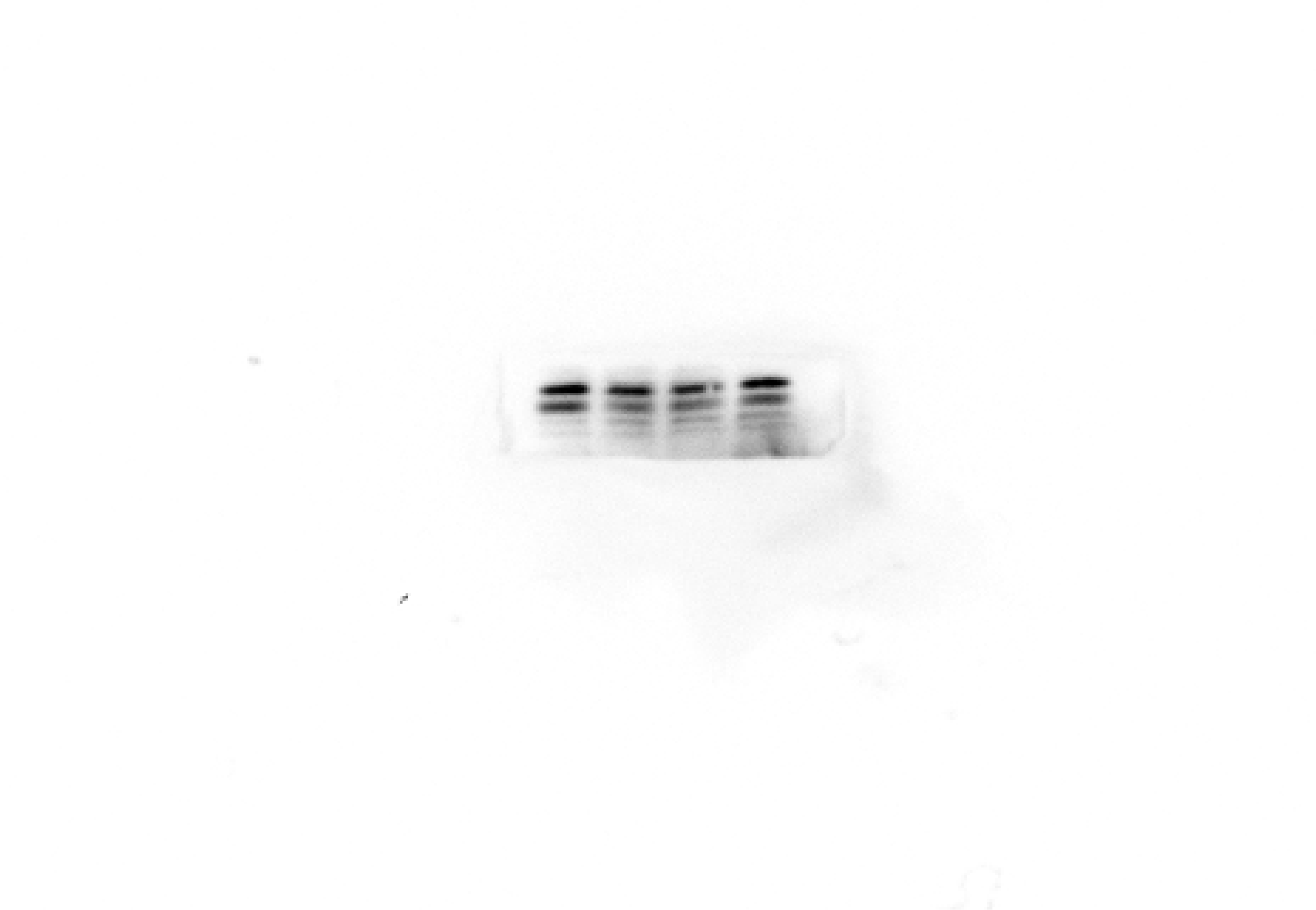

Supplement: Supplementary file 1 [file cells-15-01070-s001.zip › Supplementary File/Orginal image/Figure. 12C_CREB.png]

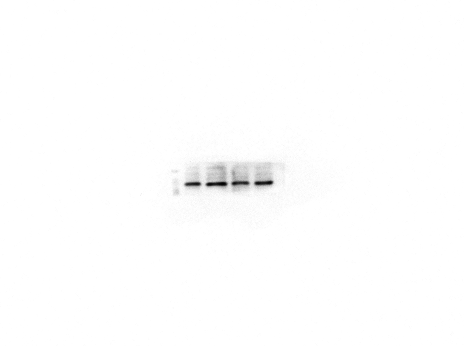

Supplement: Supplementary file 1 [file cells-15-01070-s001.zip › Supplementary File/Orginal image/Figure. 12C_GSK-3β.png]

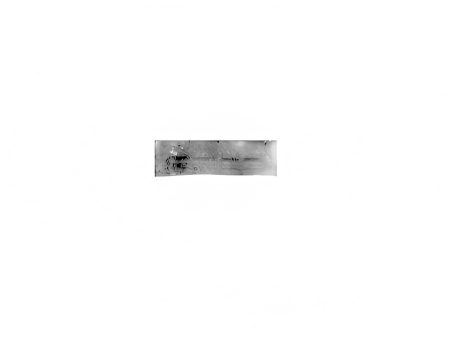

Supplement: Supplementary file 1 [file cells-15-01070-s001.zip › Supplementary File/Orginal image/Figure. 12C_p-CREB.png]

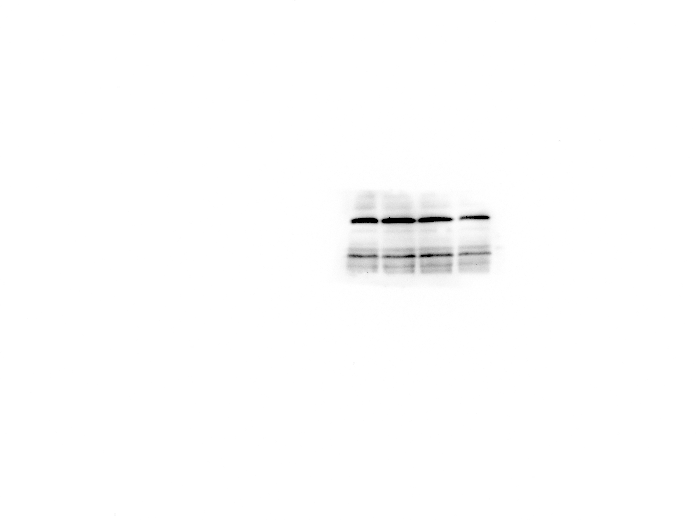

Supplement: Supplementary file 1 [file cells-15-01070-s001.zip › Supplementary File/Orginal image/Figure. 12C_p-GSK-3β.png]

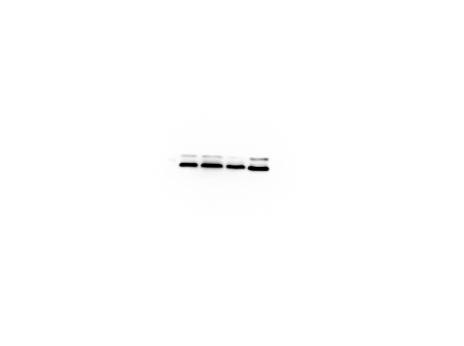

Supplement: Supplementary file 1 [file cells-15-01070-s001.zip › Supplementary File/Orginal image/Figure. 12C_β-actin.png]

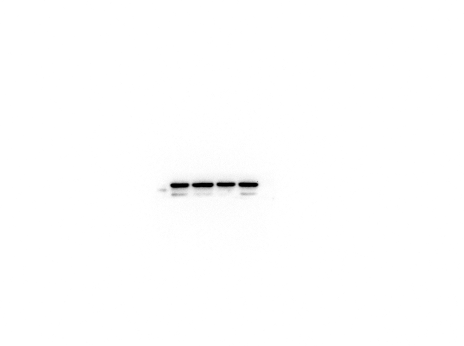

Supplement: Supplementary file 1 [file cells-15-01070-s001.zip › Supplementary File/Orginal image/Figure. 12C_β-catenin.png]

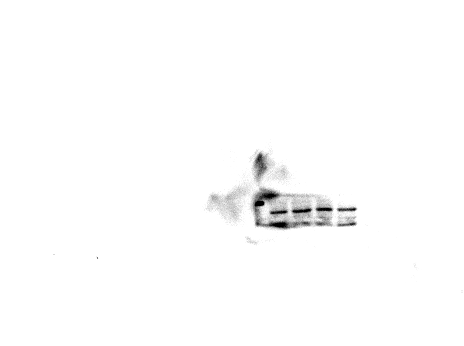

Supplement: Supplementary file 1 [file cells-15-01070-s001.zip › Supplementary File/Orginal image/Figure. 13A_AKT.png]

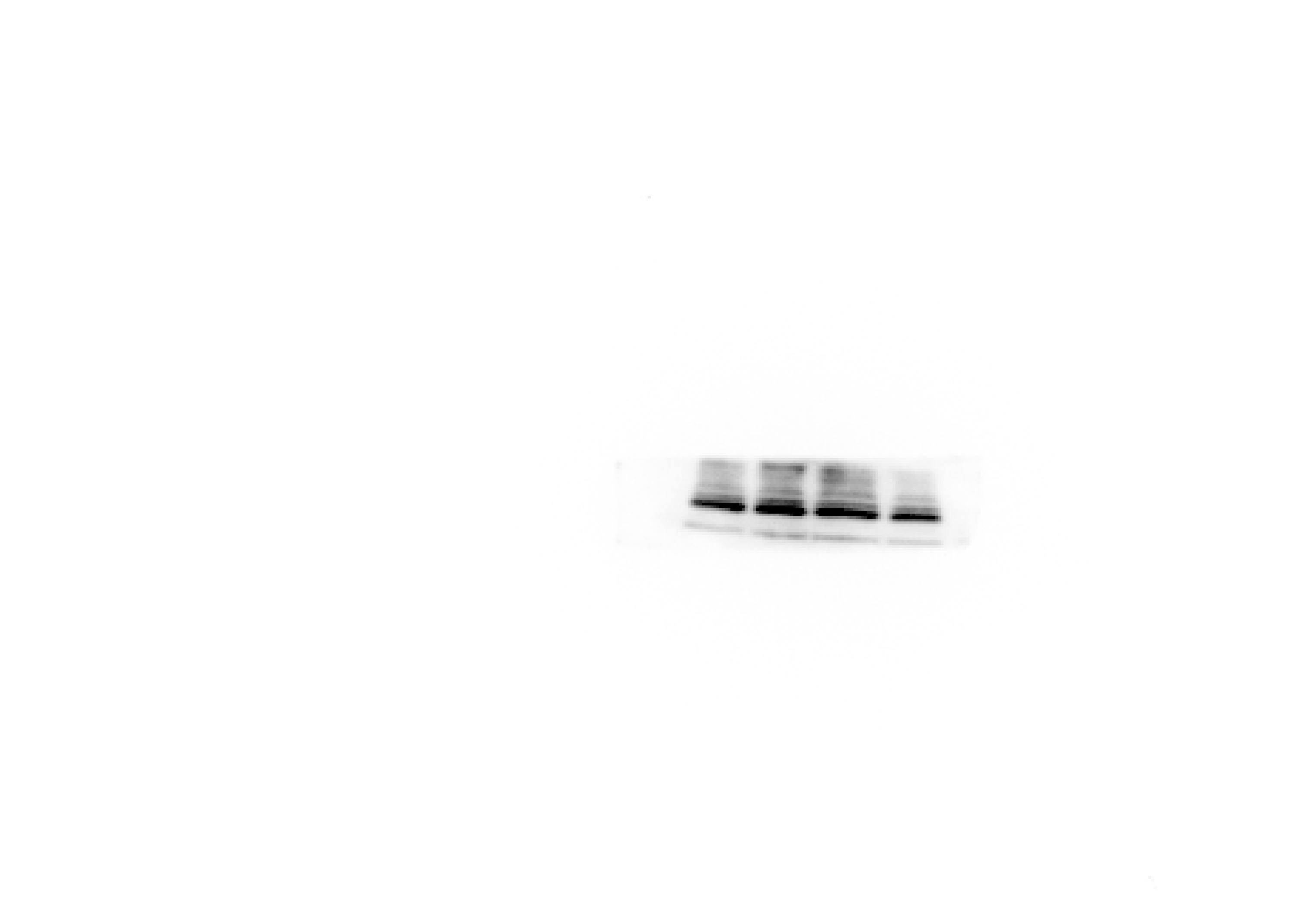

Supplement: Supplementary file 1 [file cells-15-01070-s001.zip › Supplementary File/Orginal image/Figure. 13A_ERK.png]

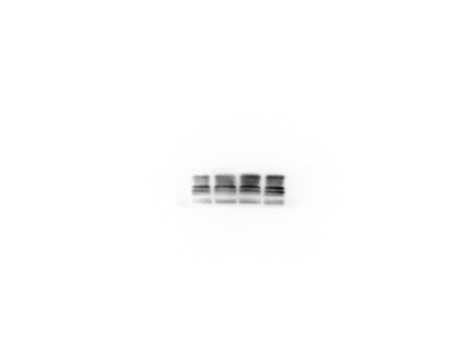

Supplement: Supplementary file 1 [file cells-15-01070-s001.zip › Supplementary File/Orginal image/Figure. 13A_p-AKT.png]

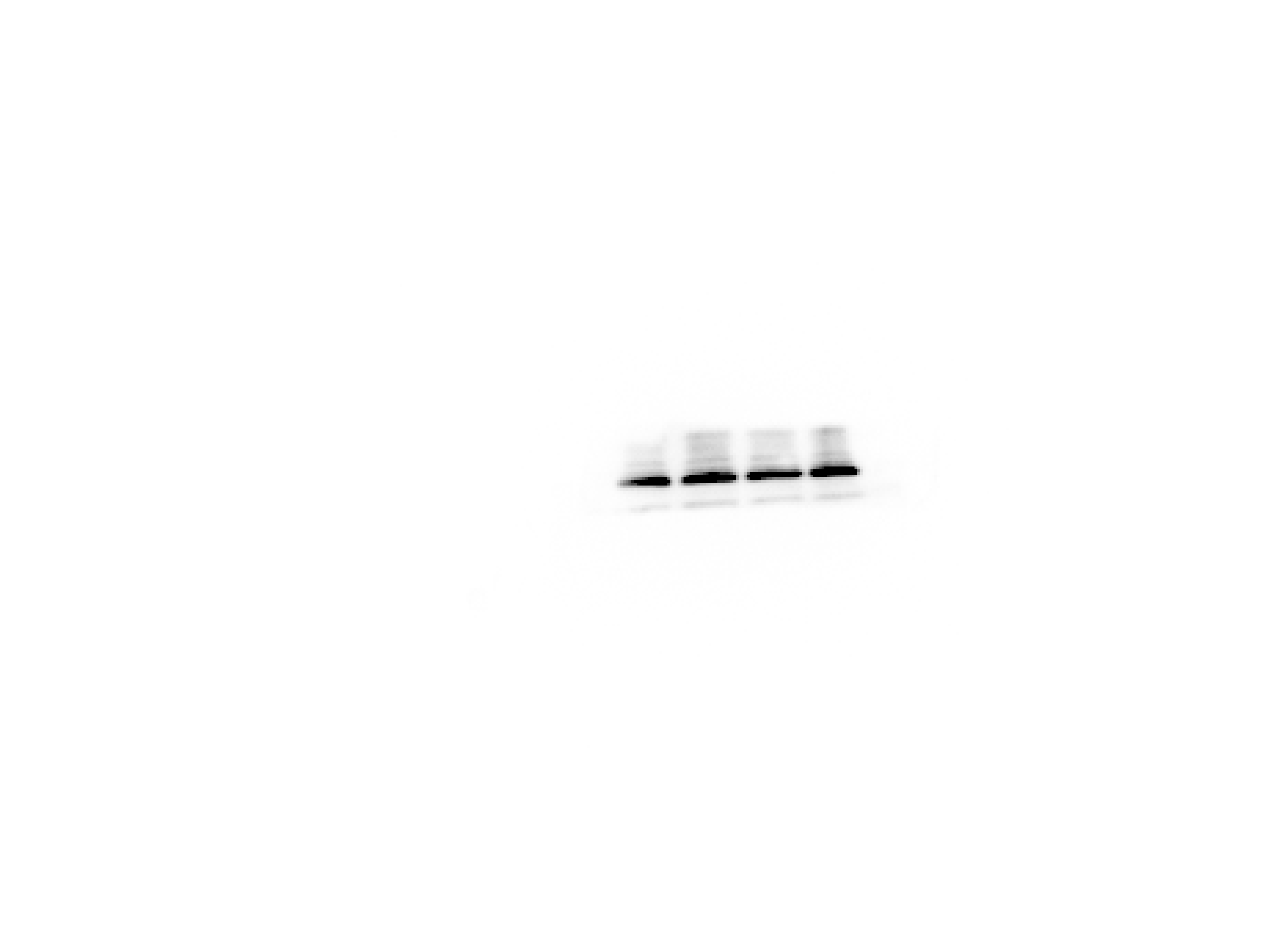

Supplement: Supplementary file 1 [file cells-15-01070-s001.zip › Supplementary File/Orginal image/Figure. 13A_p-ERK.png]

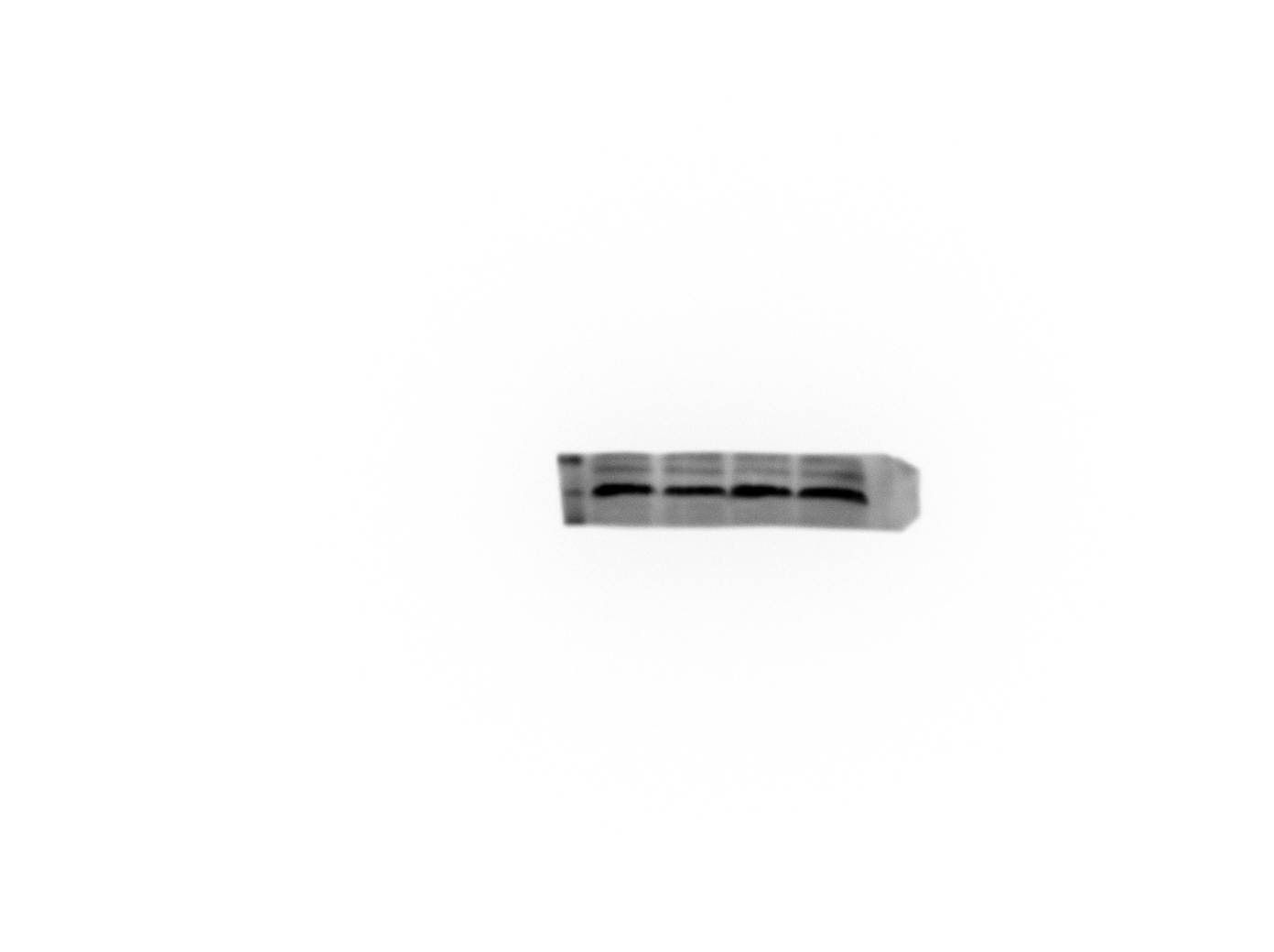

Supplement: Supplementary file 1 [file cells-15-01070-s001.zip › Supplementary File/Orginal image/Figure. 13A_β-actin.png]

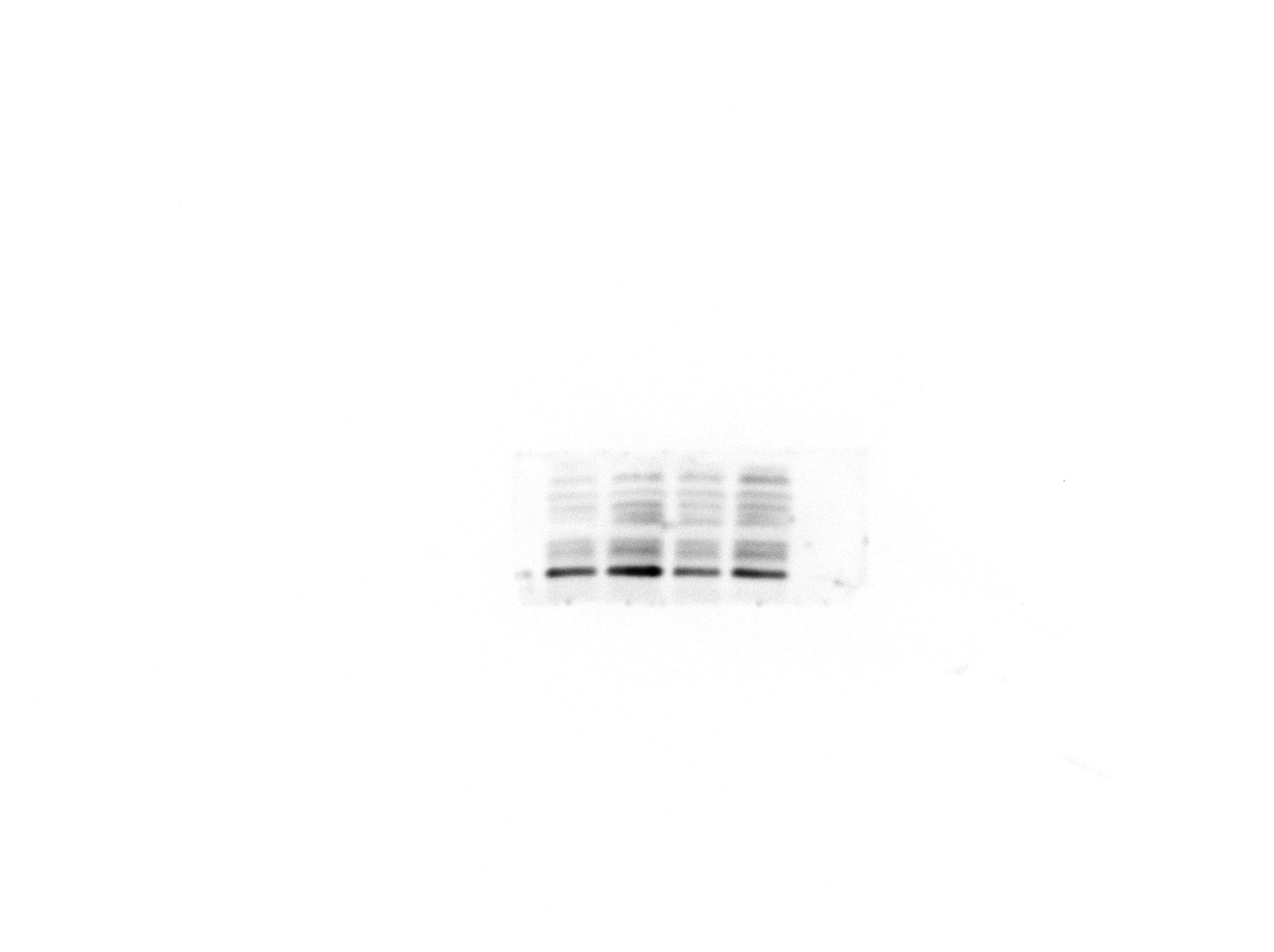

Supplement: Supplementary file 1 [file cells-15-01070-s001.zip › Supplementary File/Orginal image/Figure. 13C_AKT.png]

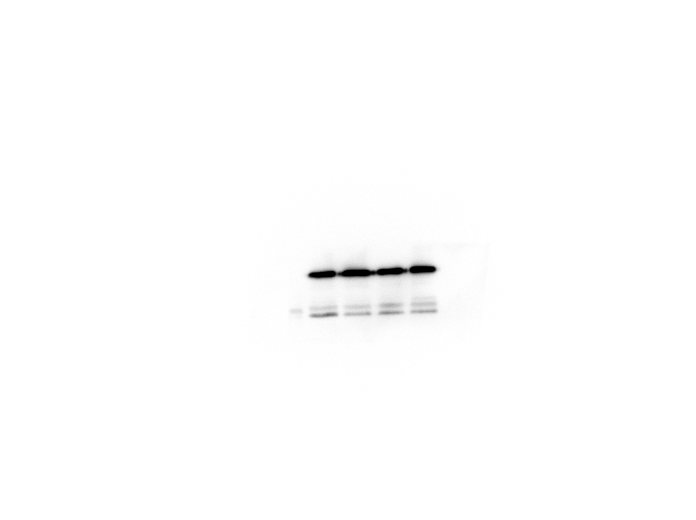

Supplement: Supplementary file 1 [file cells-15-01070-s001.zip › Supplementary File/Orginal image/Figure. 13C_ERK.png]

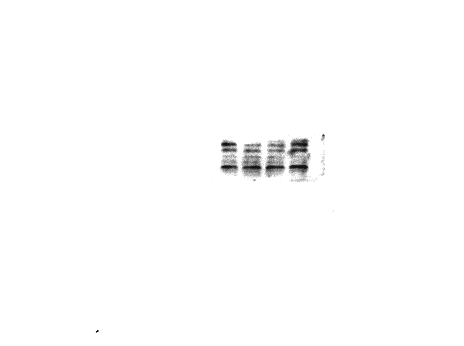

Supplement: Supplementary file 1 [file cells-15-01070-s001.zip › Supplementary File/Orginal image/Figure. 13C_p-AKT.png]

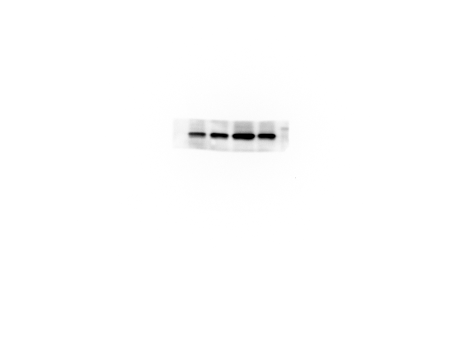

Supplement: Supplementary file 1 [file cells-15-01070-s001.zip › Supplementary File/Orginal image/Figure. 13C_p-ERK.png]

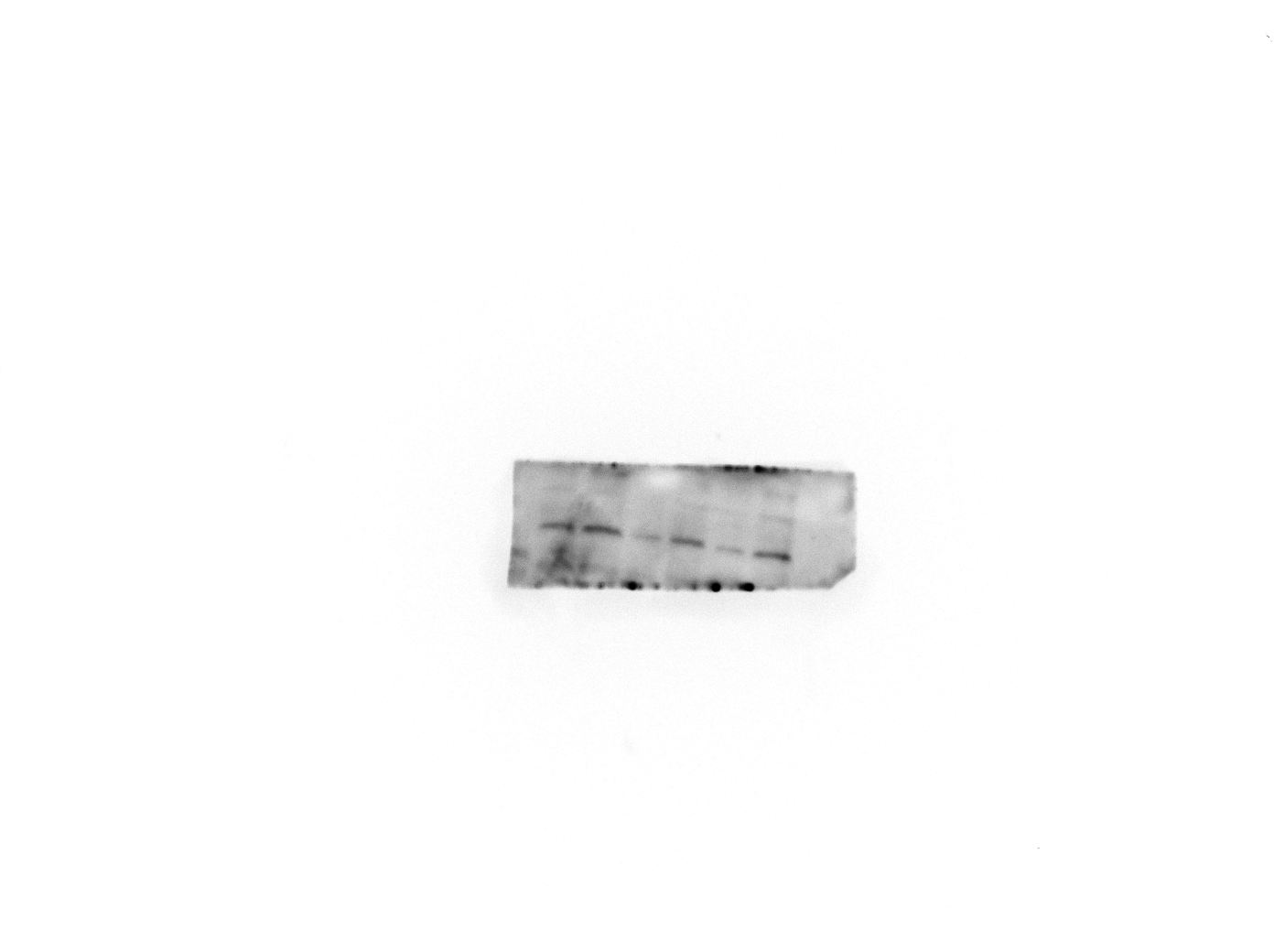

Supplement: Supplementary file 1 [file cells-15-01070-s001.zip › Supplementary File/Orginal image/Figure. 14C_MITF.png]

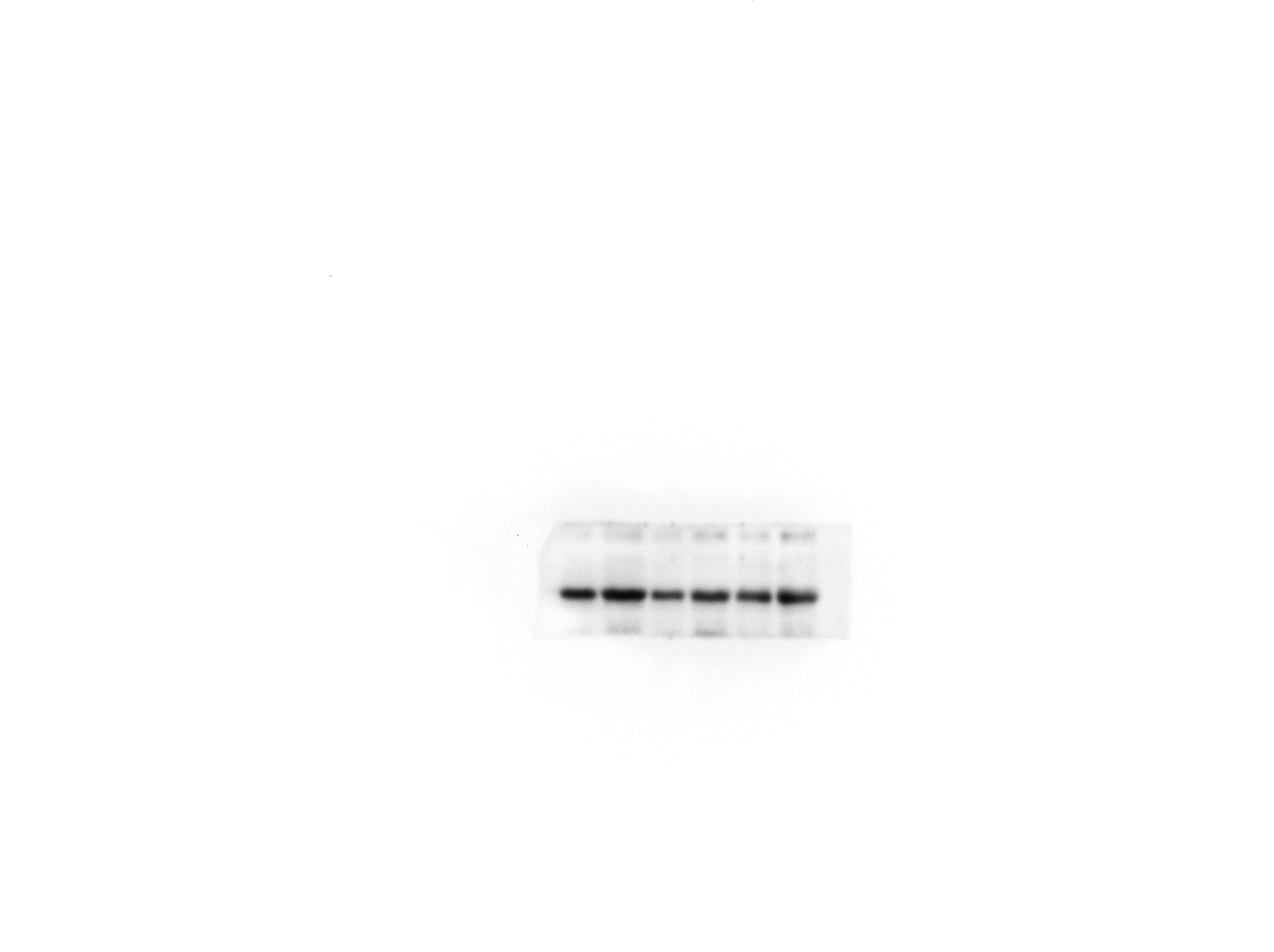

Supplement: Supplementary file 1 [file cells-15-01070-s001.zip › Supplementary File/Orginal image/Figure. 14C_TRP1.png]

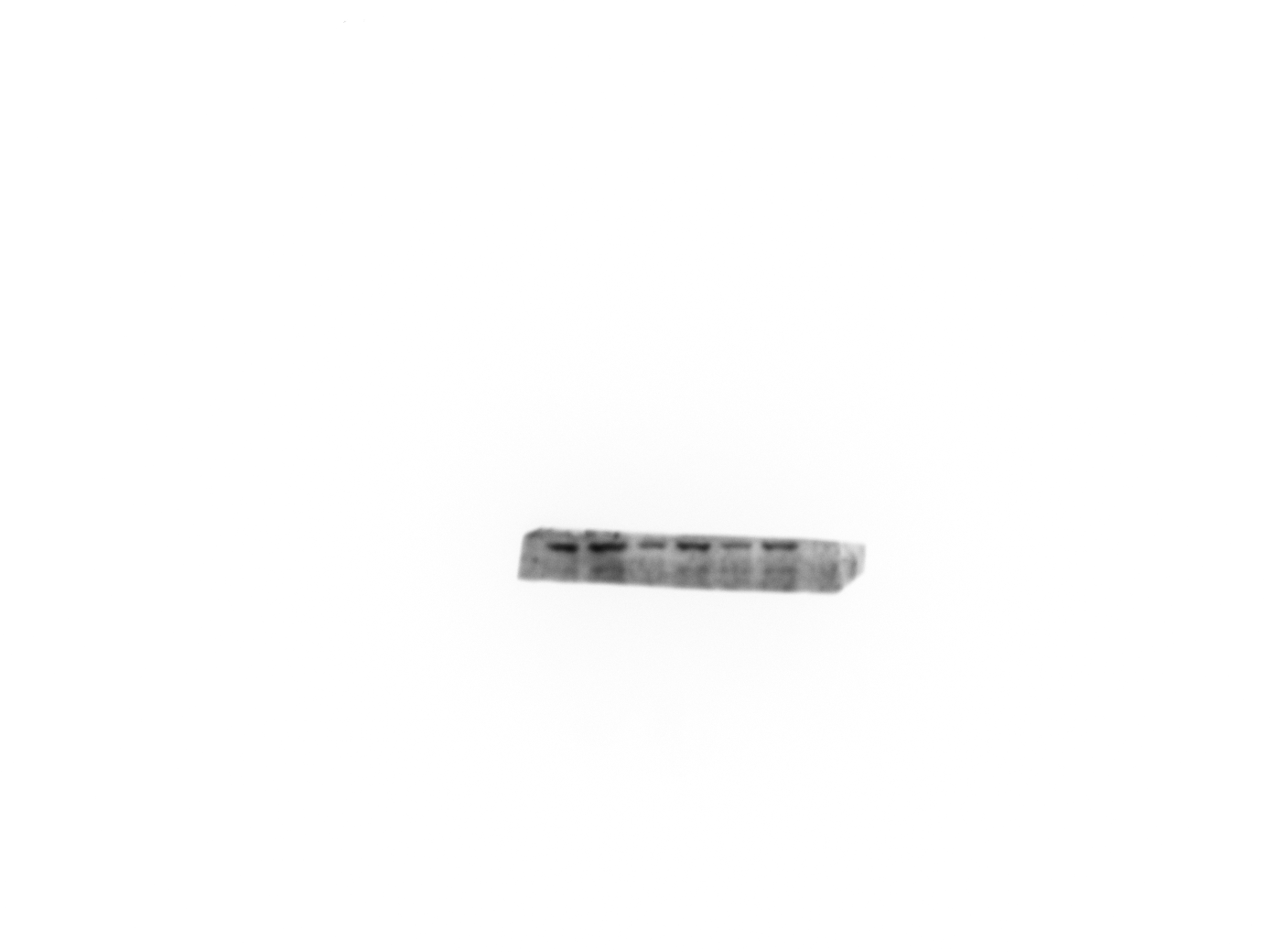

Supplement: Supplementary file 1 [file cells-15-01070-s001.zip › Supplementary File/Orginal image/Figure. 14C_TRP2.png]

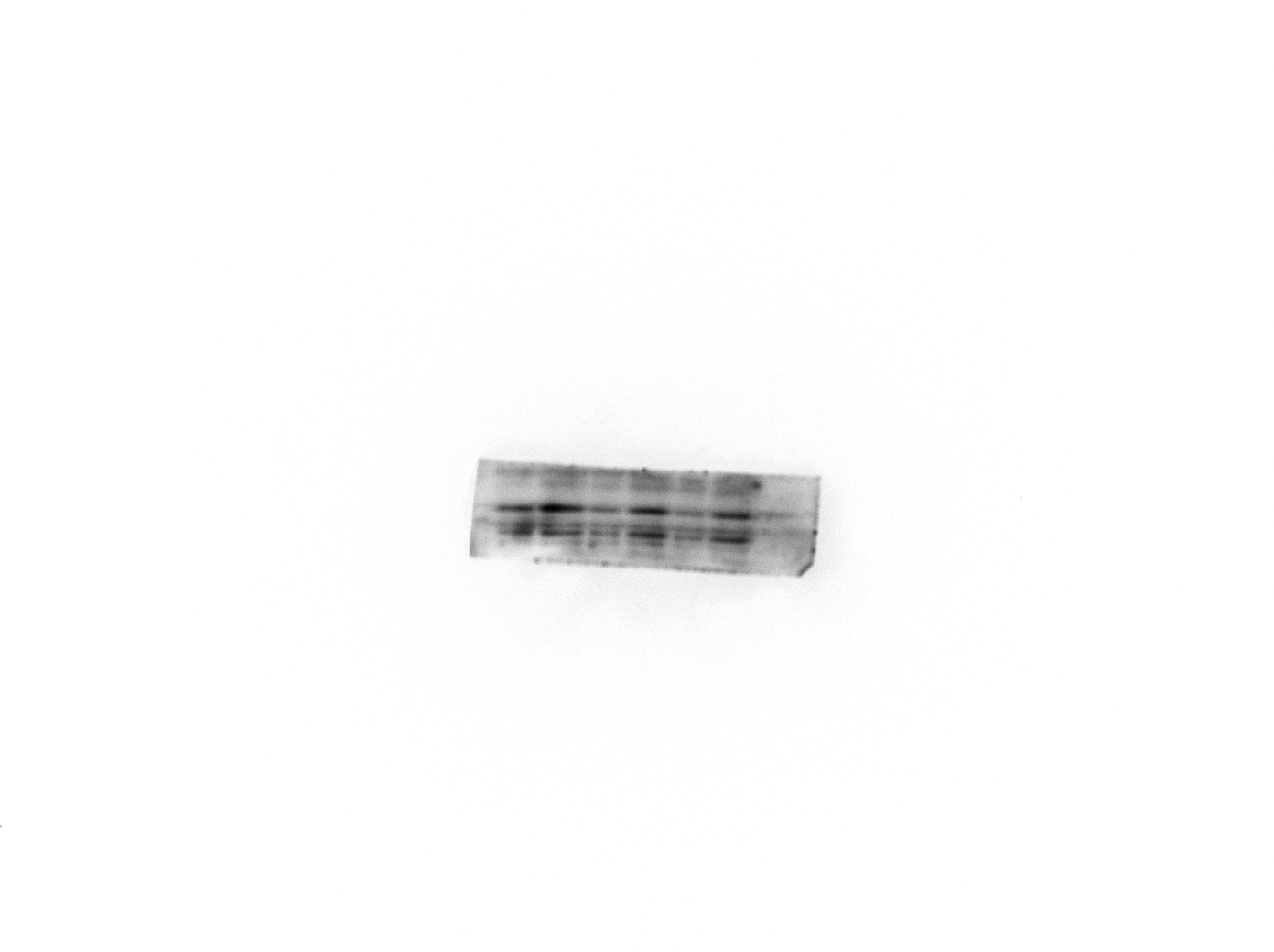

Supplement: Supplementary file 1 [file cells-15-01070-s001.zip › Supplementary File/Orginal image/Figure. 14C_TYR.png]

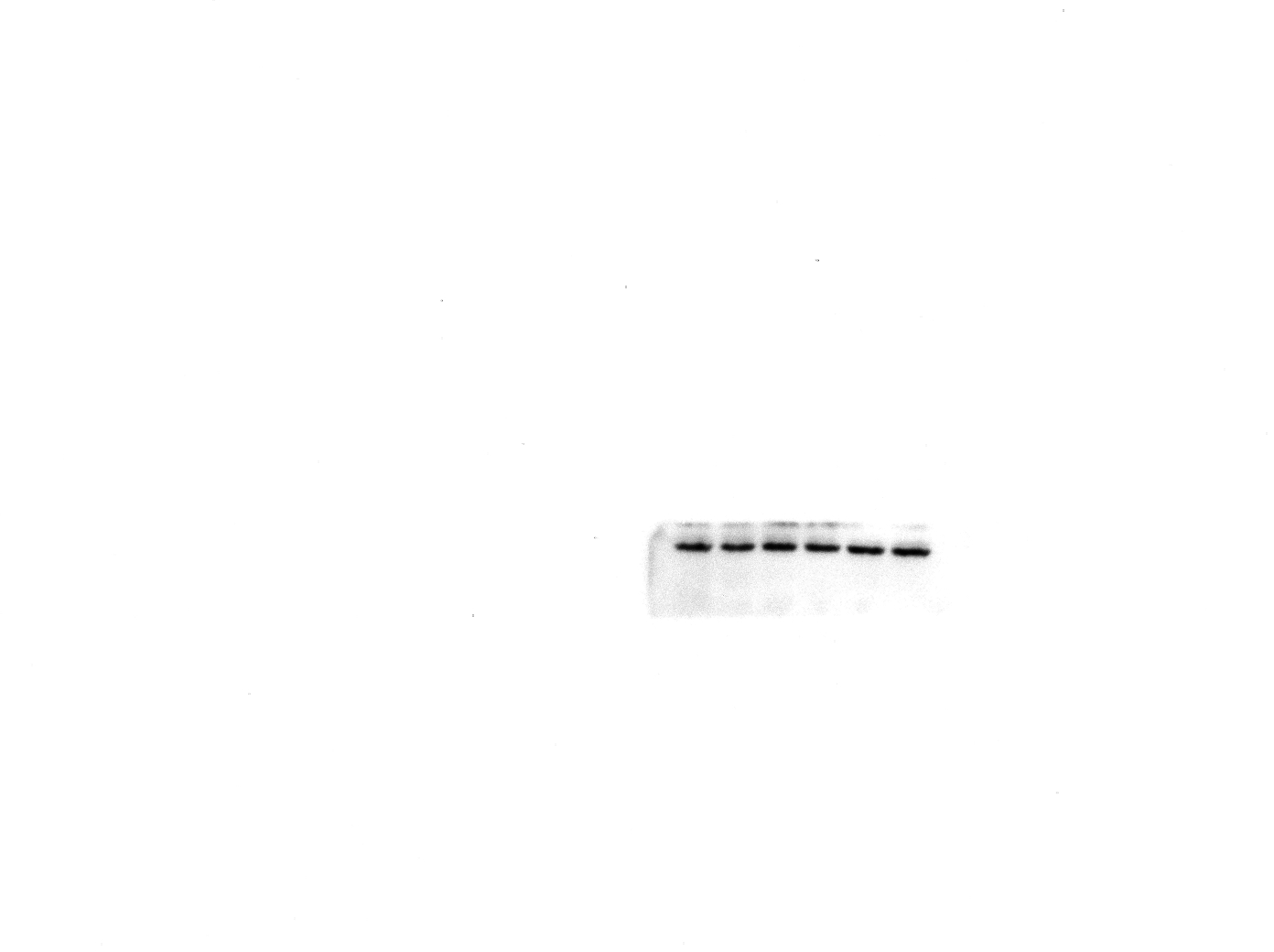

Supplement: Supplementary file 1 [file cells-15-01070-s001.zip › Supplementary File/Orginal image/Figure. 14C_β-actin.png]
